# Supplementary material for: HDAC6 deacetylates TRIM56 to negatively regulate cGAS-STING-mediated type I interferon responses
Source: EMBO Rep. 2025 Jan 2;26(3):720–47. doi: 10.1038/s44319-024-00358-5 (PMC11811133; doi:10.1038/s44319-024-00358-5)
Supplement: Supplementary file 1 — Appendix [file 44319_2024_358_MOESM1_ESM.pdf]

HDAC6 Deacetylates TRIM56 to Negatively Regulate cGAS-mediated type I Interferon Response

Appendix

Appendix Figure S1 2
Appendix Figure S2 3
Appendix Figure S3 4
Appendix Figure S4 5
Appendix Figure S5 6
Appendix Figure S6 7
Appendix Table S1 8
Appendix Table S2 8

Appendix Figure S1

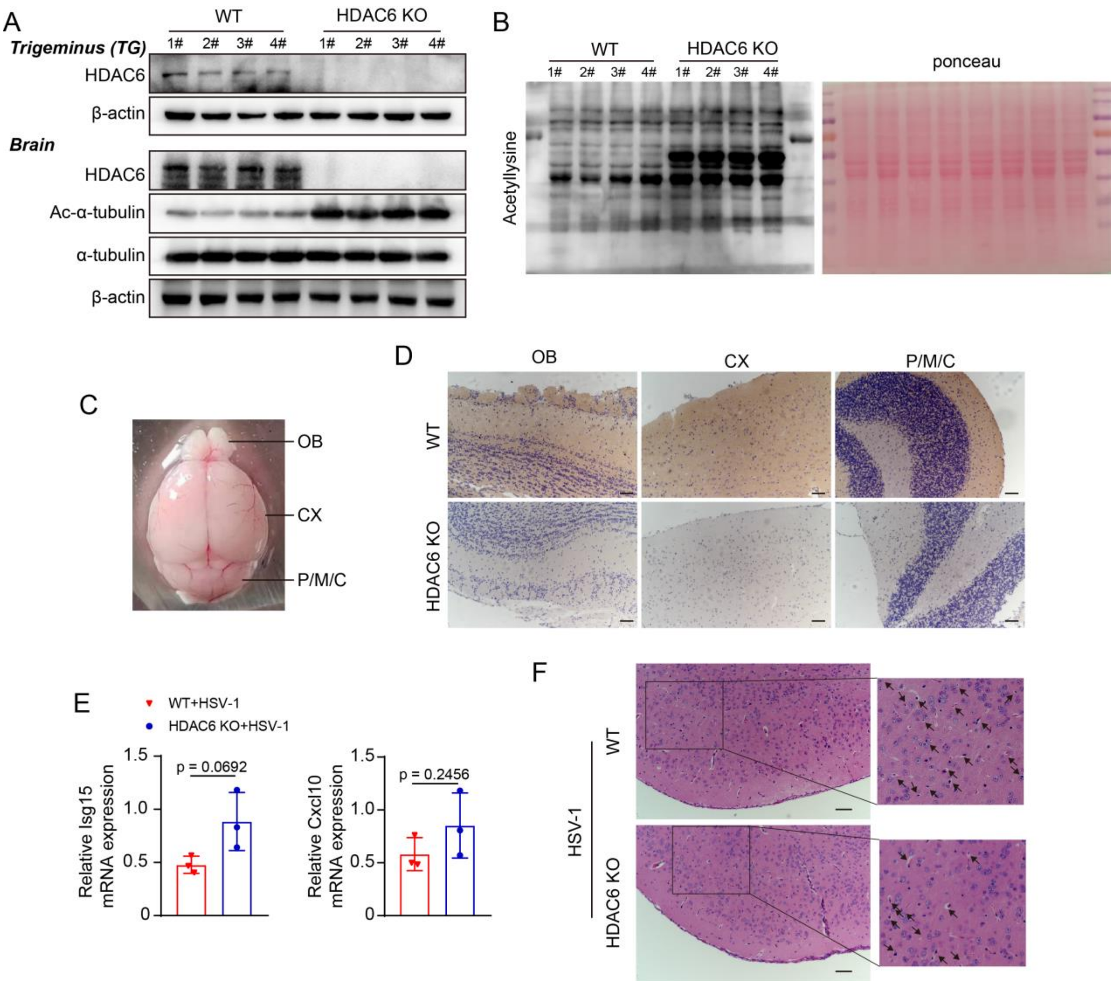

Appendix Figure S1 HDAC6 KO mice are resistant to HSV-1 infection. **A,** Western blot assay confirms the deficient expression of HDAC6 in the trigeminus (TG) and brain tissue of HDAC6 KO or wild type (WT) mice (n=4 mice per group). **B,** Western blot was used to assess the level of acetyl-lysine in brain tissues from HDAC6 KO or WT mice (n=4 mice per group). **C,** Distribution of OB (olfactory bulb), CX (cerebral cortex) and P/M/C (pons, medulla, and cerebellum) in brain tissue. **D,** Immunohistochemistry analysis of the HDAC6 expression in OB, CX and P/M/C tissues (n=4 mice per group). **E,** RT-qPCR analysis of *Isg15* and *Cxcl10* expression in HDAC6 KO or WT mice. Data were analyzed using the unpaired t-test, which are shown as mean ± SD (n = 3 mice per group). **F,** Representative images of H&E staining of cerebral cortex tissues from HSE mice (n=3). The black arrow points to cytoplasmic vacuolization. Scale bar, 100 µm.

Appendix Figure S2

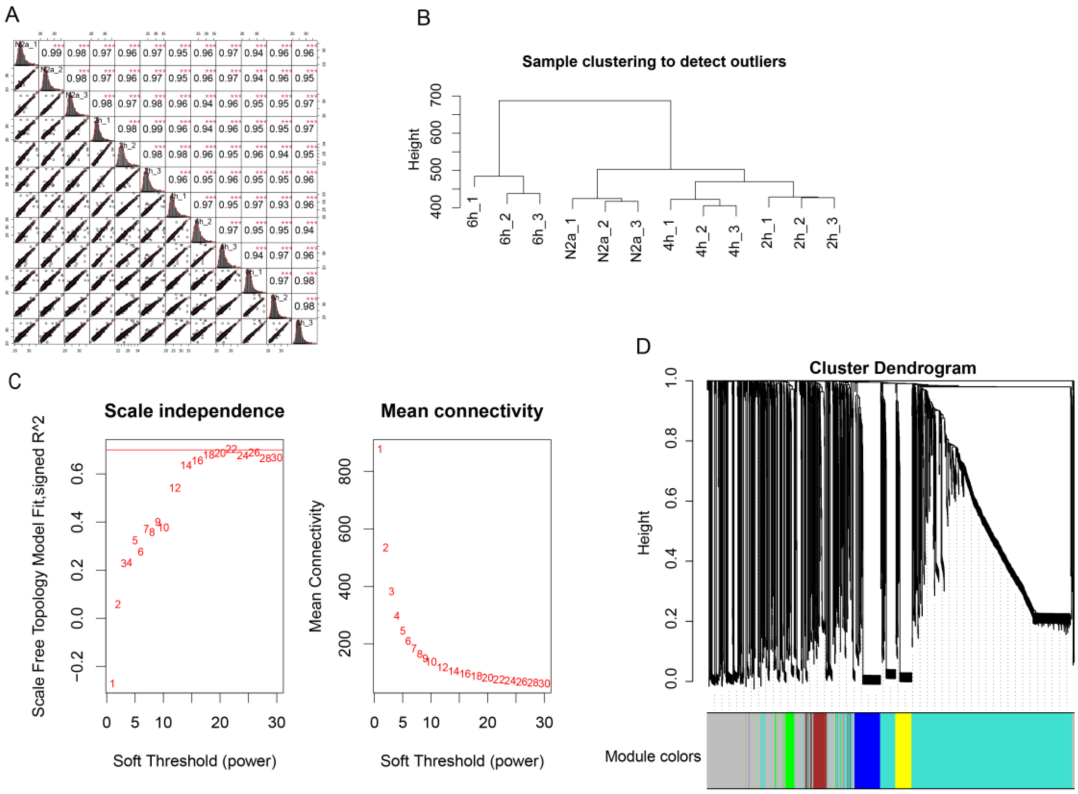

Appendix Figure S2 Weighted gene co-expression network (WGCNA) was used to analyze acetylation sites. **A,** Acetylome correlation scatter plots and histograms of data distribution between samples. **B,** Sample clustering to detect outliers. **C,** Determination of soft-thresholding power in weighted gene co-expression network analysis (WGCNA). The left panel shows the scale-free fit index (y-axis) as a function of soft threshold power (x-axis), and the right panel shows network connectivity in the case of different soft thresholds. A soft threshold of $\beta = 22$ was used to define the adjacency matrix. **D,** Hierarchical clustering tree of module genes drawn differently based on topological overlap, with different modules represented in different colors.

Appendix Figure S3

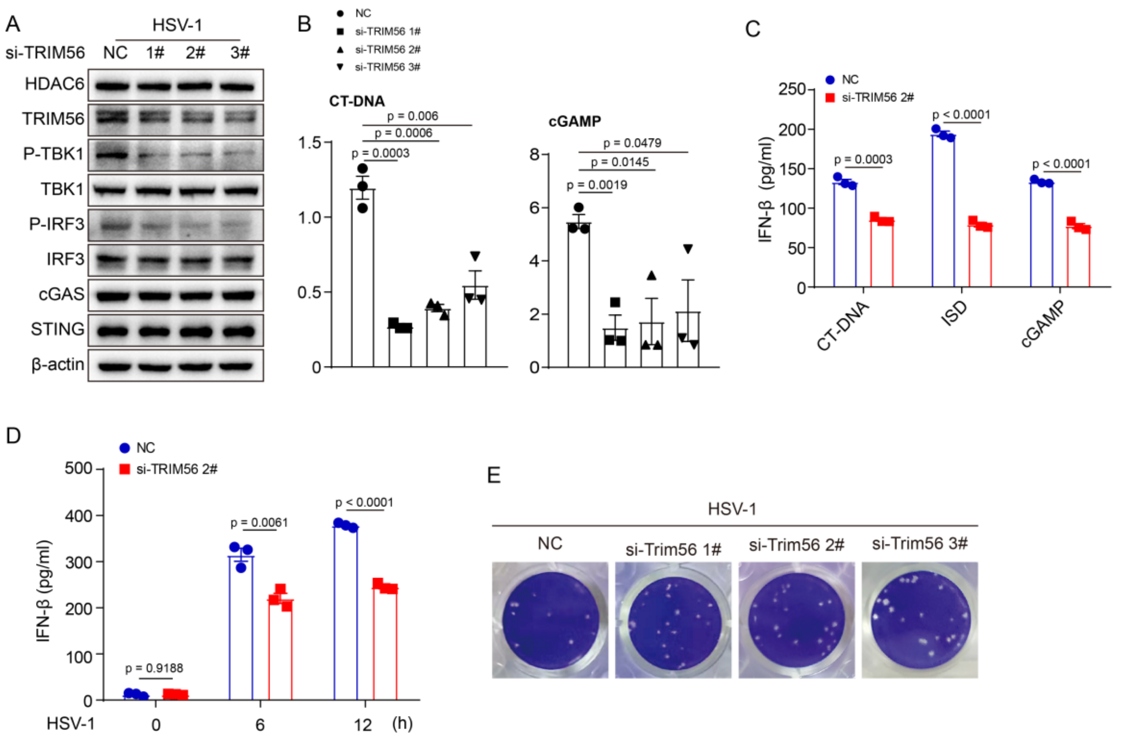

Appendix Figure S3 TRIM56 enhances the cGAS-STING signaling pathway for antiviral activity. **A,** Western blot analysis of the cGAS-STING signaling pathway in N2a cells transfected with TRIM56 siRNAs followed by infection with HSV-1 (MOI = 3) for 12 h. **B,** RT-qPCR assay detection of the *Ifnb1* mRNA expression in TRIM56 siRNAs-transfected cells treated with CT-DNA (2 μg/ml) or cGAMP (2 μg/ml) for 6 h. Data were analyzed using the unpaired t-test, which are shown as mean ± SD (n = 3 biological replicates). **C-D,** ELISA assay of IFN-β production in the culture supernatant of N2a cells stimulated with CT-DNA, ISD or 3'-3'-cGAMP (2 μg/ml) for 6 h (C) or infected with HSV-1 (MOI = 3) for the indicated time (D). Data were analyzed using the unpaired t-test, which are shown as mean ± SD (n = 3 biological replicates). **E,** Viral plaque assays of N2a cells transfected with TRIM56 siRNAs followed by infection with HSV-1 (MOI = 3) for 12 h.

Appendix Figure S4

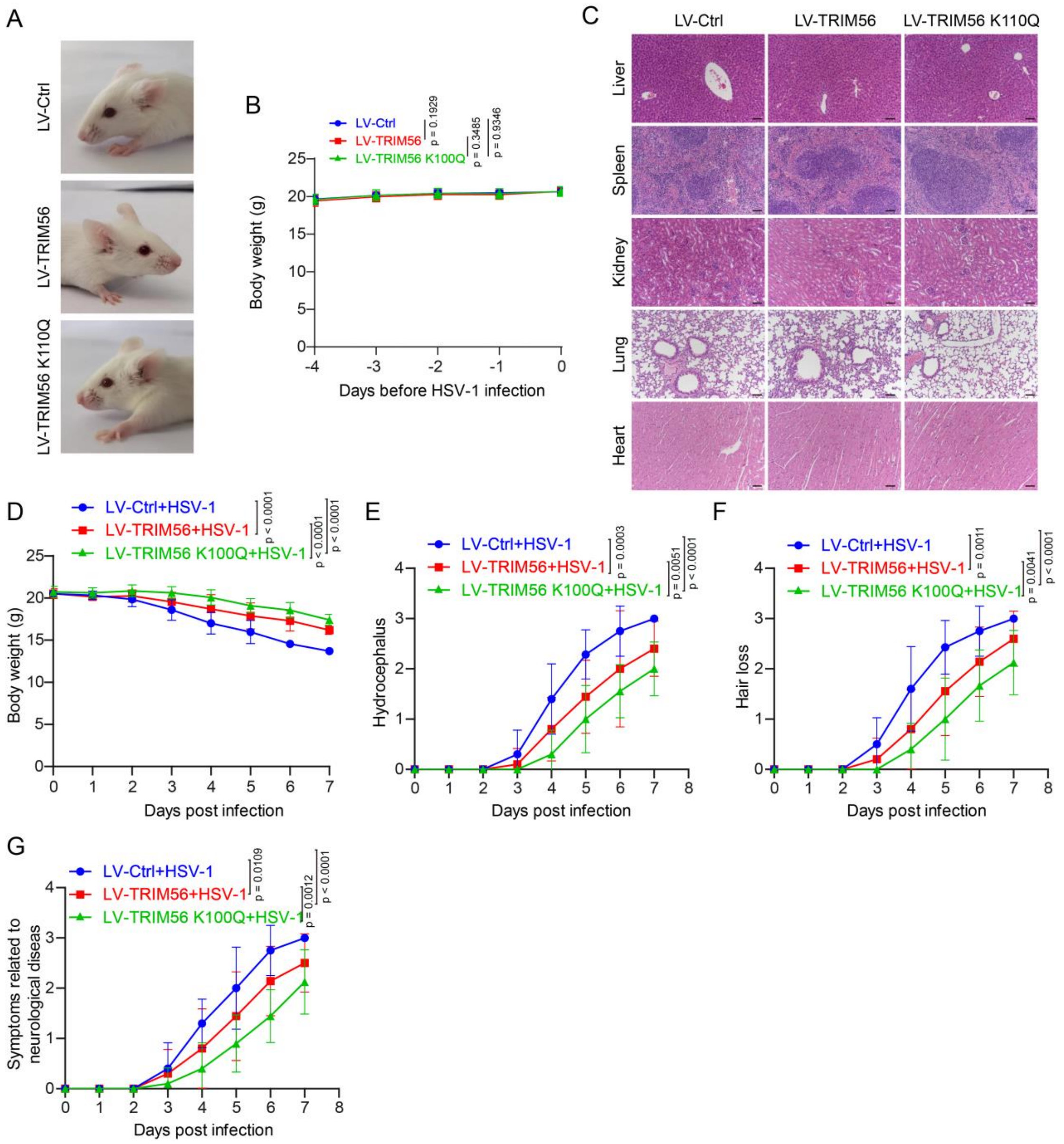

Appendix Figure S4 Characterization of mice overexpressing TRIM56. **A,** Photographic records of the eyes and brain symptoms from lentivirus-infected mice on the day 4 (n=5 mice per group). **B,** Body weight of lentivirus-infected mice was recorded daily before HSV-1 infection (n=16 mice per group). **C,** Representative H&E staining images of brain tissues from lentivirus-infected mice. Scale bar, 100 μm. **D-G**, Body weight (D) and HSE symptoms (E-G) were daily recorded. Data were analyzed using Two-way ANOVA (D-G), which are shown as mean ± SD (n = 10 mice per group).

Appendix Figure S5

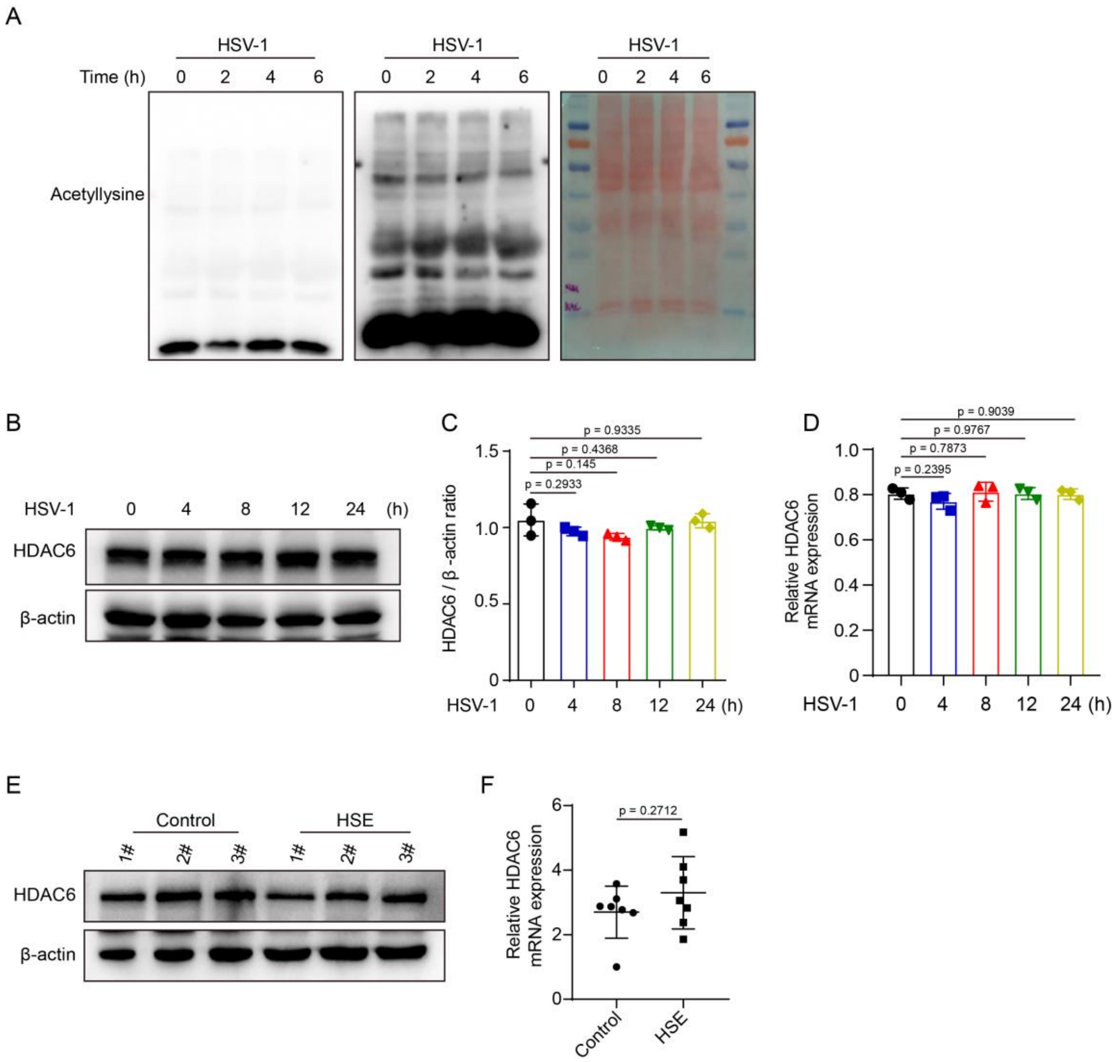

Appendix Figure S5 HSV-1 infection does not affect the expression of HDAC6 *in vivo* and *in vitro*. **A,** Western blot analysis of acetyl-lysine protein in N2a cells infected with HSV-1 (MOI = 10) for the indicated time (shorter and longer exposure). Ponceau staining on the right. **B-D,** N2a cells were infected with HSV-1 (MOI=1) for the indicated times, and western blot and RT-qPCR assay was performed to detect the expression of HDAC6. Data are shown as mean ± SD (n=3 biological replicates), unpaired t-test. **E,** Western blot analysis of HDAC6 protein levels in brain tissues. **F,** RT-qPCR analysis of the HDAC6 mRNA expression. Data were analyzed using the unpaired t-test, which are shown as mean ± SD (n = 7 mice per group).

Appendix Figure S6

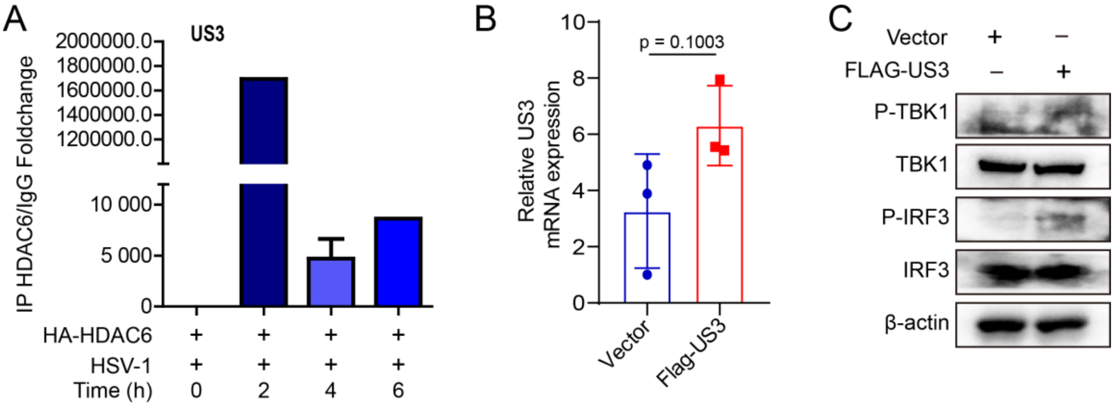

Appendix Figure S6 US3 interacts with HDAC6. **A,** The interaction strength of US3-HDAC6 at different time points post-HSV-1 (MOI = 10) infection. **B,** The mRNA expression of US3 in N2a cells transfected with plasmid Flag-US3. Data were analyzed using the unpaired t-test, which are shown as mean ± SD (n = 3 biological replicates). **C,** Western blot analysis of TBK-IRF3 signaling in N2a cells transfected with FLAG-US3 for 72 h.

Appendix Table S1

Energy Component	Average		Std. Dev.		Std. Err. of Mean	
	WT	K110A	WT	K110A	WT	K110A
VDWAALS	-85.59	-67.42	9.95	5.15	1.95	1.01
EEL	-900.11	-424.04	33.40	55.66	6.55	10.92
EGB	946.67	469.81	33.41	53.72	6.55	10.54
ESURF	-11.35	-8.12	1.13	0.79	0.22	0.16
DELTA G gas	-985.70	-491.46	35.72	55.74	7.01	10.93
DELTA G solv	935.32	461.69	33.21	53.36	6.51	10.46
DELTA TOTAL	-50.38	-29.76	8.27	5.47	1.62	1.07

Appendix Table S1 Different binding free energies values in kcal/mol for screened proteins predicted by MM-GBSA. VDWAALS (van der Waals energy), EEL (electrostatic energy), EGB (MMGBSA polar solvation energy), ESURF (MMGBSA non-polar solvation energy), DELTA G gas (net gas phase energy), DELTA G solv (net solvation energy), DELTA TOTAL (net system energy).

Appendix Table S2

Energy Component	Average		Std. Dev.		Std. Err. of Mean	
	WT	K110T	WT	K110T	WT	K110T
VDWAALS	-78.79	-115.77	10.95	11.15	2.15	2.19
EEL	-108.54	-155.64	91.21	121.48	17.89	23.82
EGB	167.66	246.44	94.20	117.39	18.47	23.02
ESURF	-11.30	-16.87	1.42	2.10	0.28	0.41
DELTA G gas	-187.33	-271.41	98.34	128.29	19.29	25.16
DELTA G solv	156.35	229.57	93.06	115.56	18.25	22.66
DELTA TOTAL	-30.98	-41.83	10.15	14.66	1.99	2.87

Appendix Table S2 Different binding free energies values in kcal/mol for screened proteins predicted by MM-GBSA. VDWAALS (van der Waals energy), EEL (electrostatic energy), EGB (MMGBSA polar solvation energy), ESURF (MMGBSA non-polar solvation energy), DELTA G gas (net gas phase energy), DELTA G solv (net solvation energy), DELTA TOTAL (net system energy).
