# Supplementary material for: HDAC6 deacetylates TRIM56 to negatively regulate cGAS-STING-mediated type I interferon responses
Source: EMBO Rep. 2025 Jan 2;26(3):720–47. doi: 10.1038/s44319-024-00358-5 (PMC11811133; doi:10.1038/s44319-024-00358-5)
Supplement: Supplementary file 6 — Source data Fig. 1 [file 44319_2024_358_MOESM6_ESM.zip › Source data Figure 1/Figure 1A-D,F,H.docx]

**Source Figure 1A HSV-1**


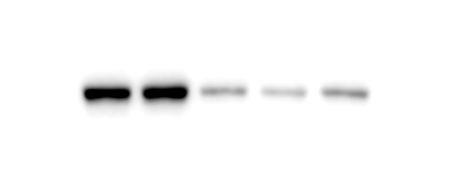

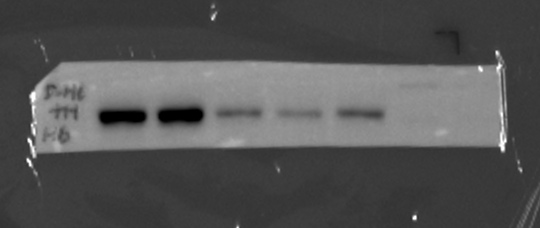


**130**

**170**

HDAC6

ICP0


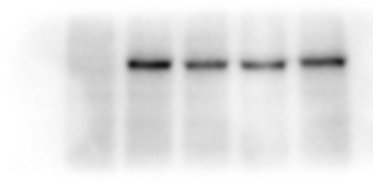

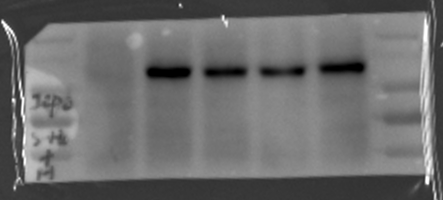


**55**

**100**

**70**

**130**

**170**

P-TBK1




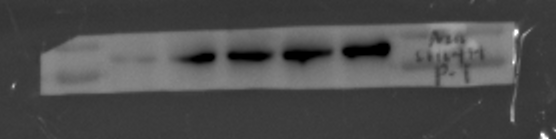


**70**

**100**


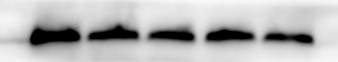

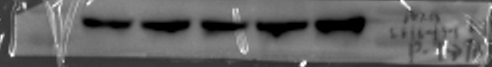


**70**

**100**

TBK1

P-IRF3


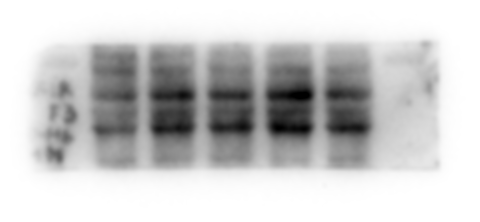

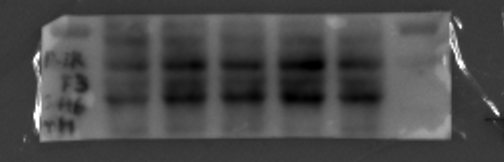


**40**

**55**

**70**




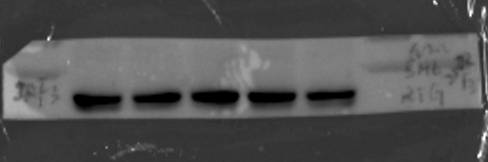


**40**

**55**

**70**

IRF3


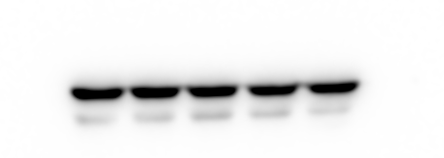

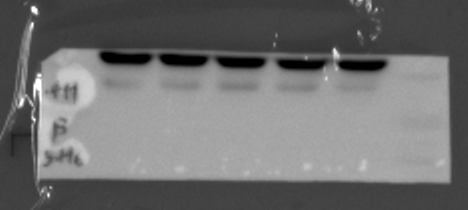


**25**

**40**

**35**

β-actin

**Source Figure 1B CT-DNA**


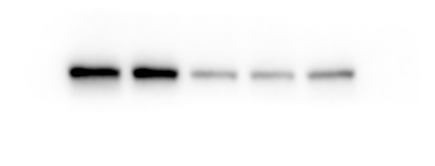

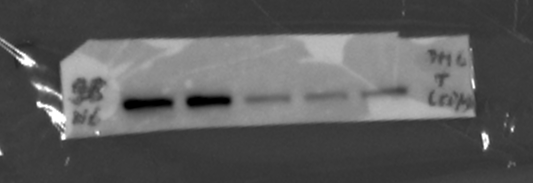


**170**

**130**

HDAC6




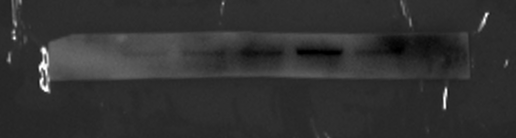


**70**

**100**

P-TBK1

TBK1


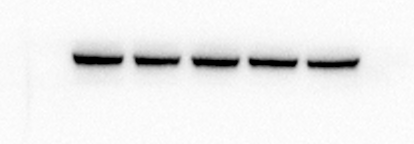

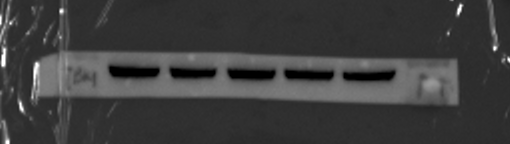


**100**

**70**


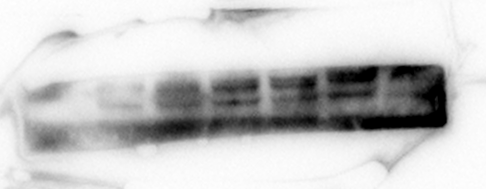

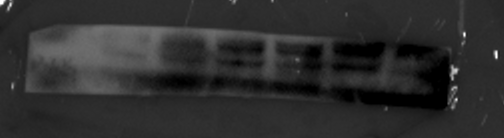


**40**

**55**

P-IRF3


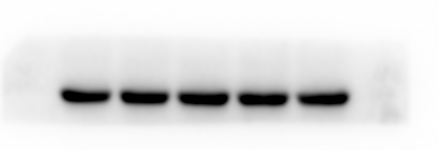

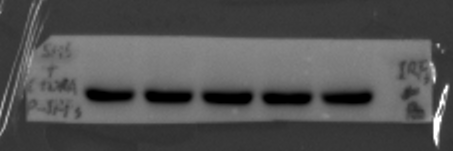


**40**

**55**

IRF3


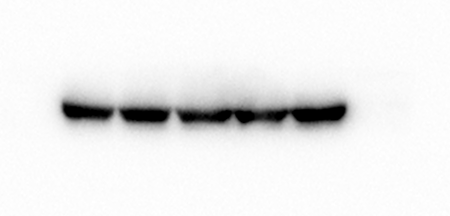

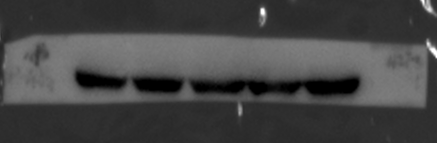


**40**

**55**

β-actin

**Source Figure 1C ISD**


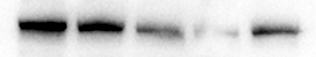

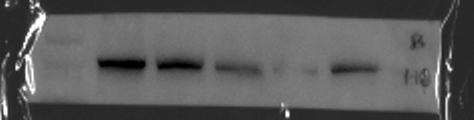


P-TBK1

HDAC6

**100**

**130**

**170**


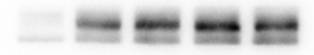

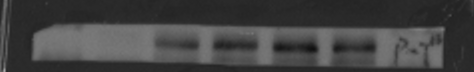


TBK1

**70**

**100**


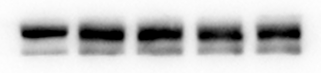

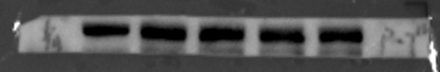


**70**

**100**


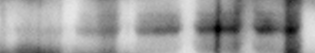

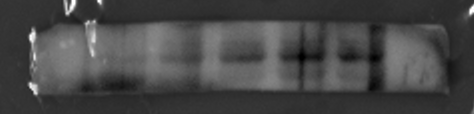


**55**

**40**

**55**

P-IRF3


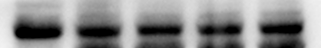

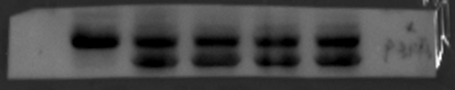


**40**

IRF3


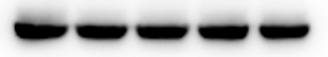

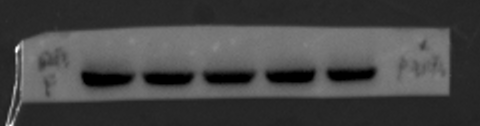


**40**

**55**

β-actin

**Source Figure 1D**




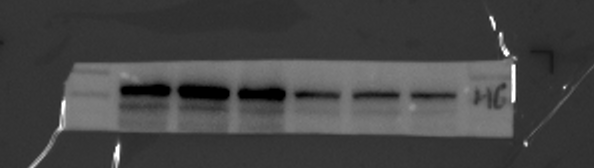


**170**

**130**

HDAC6




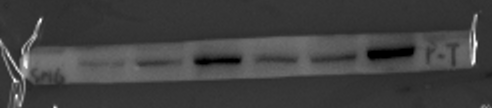


**70**

**100**

P-TBK1




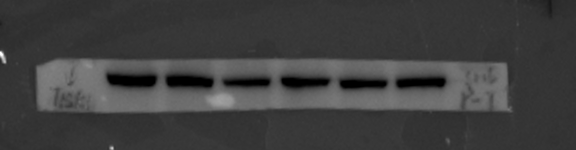


P-IRF3

**70**

**100**

TBK1


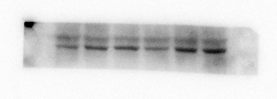

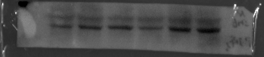


**55**

**40**


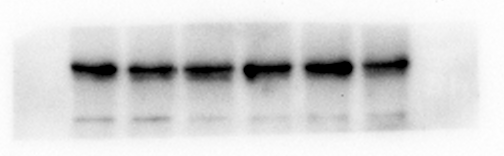

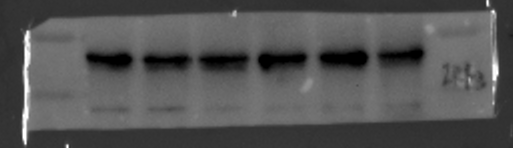


**55**

**40**

IRF3


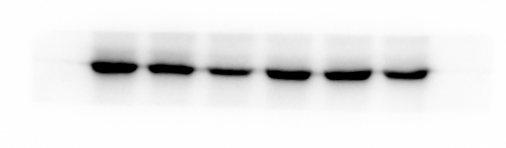

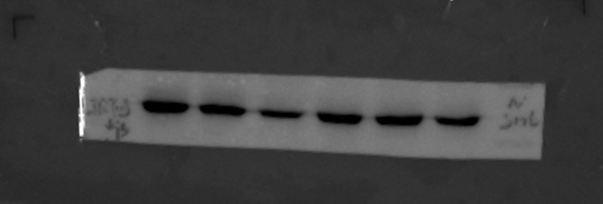


**55**

**40**

β-actin

**Source Figure 1F**


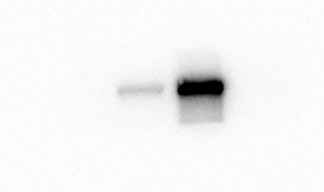

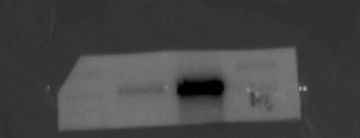


**130**

**170**

HDAC6


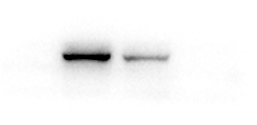

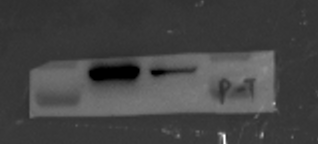


**70**

**100**

P-TBK1


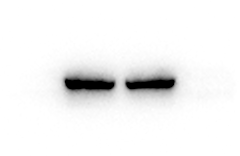

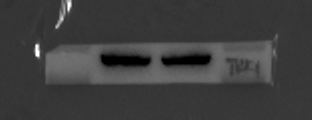


**70**

**100**

TBK1


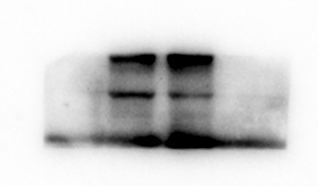

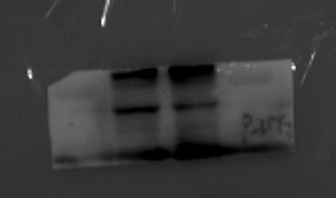


**40**

**55**

P-IRF3


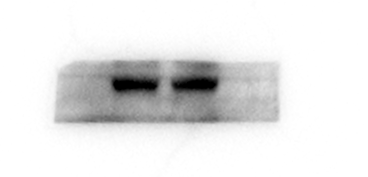

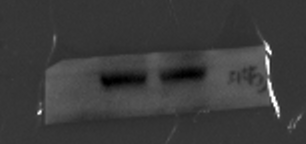


**40**

**55**

IRF3


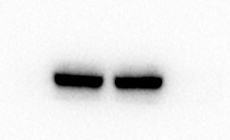

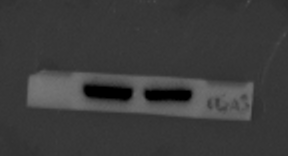


**55**

**70**

cGAS


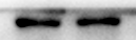

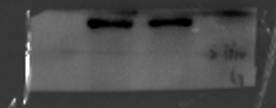


STING


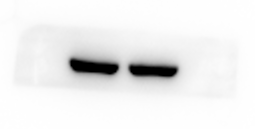

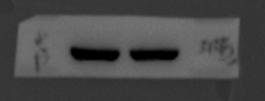


**40**

**55**

β-actin

**Source Figure 1H**


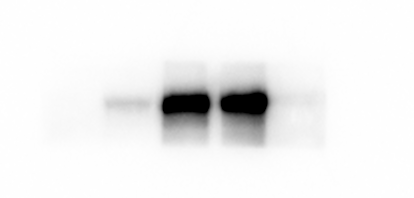

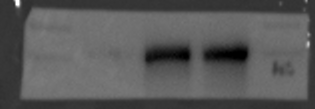


**170**

**100**

**130**

HDAC6


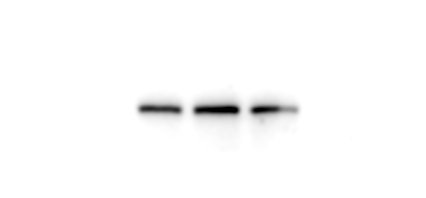

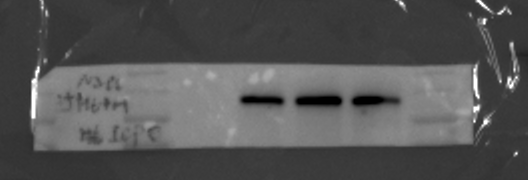


**170**

**100**

**130**

ICP0


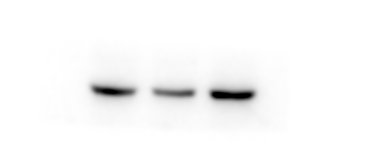

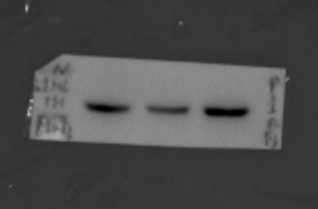


P-TBK1

**100**

**70**


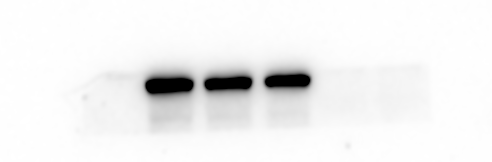

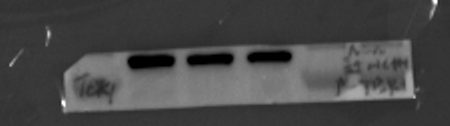


**100**

**70**

TBK1


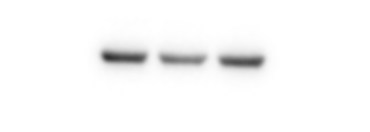

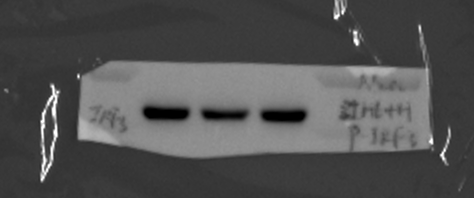


**40**

**55**

P-IRF3


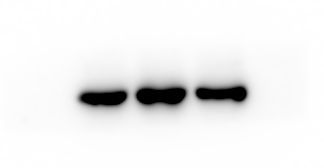

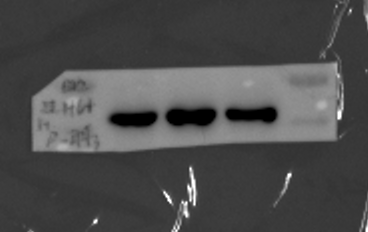


**40**

**55**

IRF3


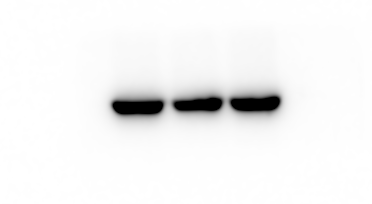

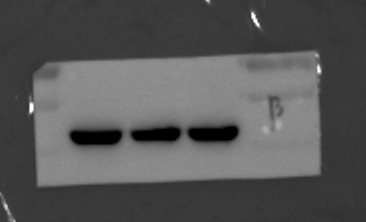


**70**

**55**

**40**

β-actin
