# Supplementary figures and images for: HDAC6 deacetylates TRIM56 to negatively regulate cGAS-STING-mediated type I interferon responses
Source: EMBO Rep. 2025 Jan 2;26(3):720–47. doi: 10.1038/s44319-024-00358-5 (PMC11811133; doi:10.1038/s44319-024-00358-5)

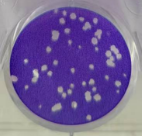

Supplement: Supplementary file 7 — Source data Fig. 2 [file 44319_2024_358_MOESM7_ESM.zip › Source data Figure 2/Figure 2A/NC+HSV-1 12h.png]

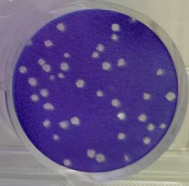

Supplement: Supplementary file 7 — Source data Fig. 2 [file 44319_2024_358_MOESM7_ESM.zip › Source data Figure 2/Figure 2A/NC+HSV-1 4h.png]

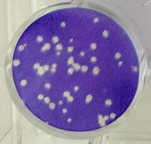

Supplement: Supplementary file 7 — Source data Fig. 2 [file 44319_2024_358_MOESM7_ESM.zip › Source data Figure 2/Figure 2A/NC+HSV-1 8h.png]

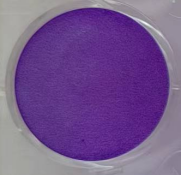

Supplement: Supplementary file 7 — Source data Fig. 2 [file 44319_2024_358_MOESM7_ESM.zip › Source data Figure 2/Figure 2A/NC.png]

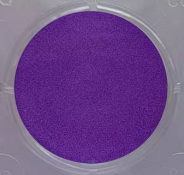

Supplement: Supplementary file 7 — Source data Fig. 2 [file 44319_2024_358_MOESM7_ESM.zip › Source data Figure 2/Figure 2A/S-1.png]

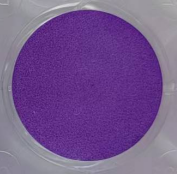

Supplement: Supplementary file 7 — Source data Fig. 2 [file 44319_2024_358_MOESM7_ESM.zip › Source data Figure 2/Figure 2A/S-2.png]

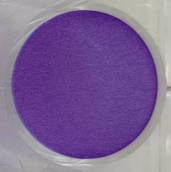

Supplement: Supplementary file 7 — Source data Fig. 2 [file 44319_2024_358_MOESM7_ESM.zip › Source data Figure 2/Figure 2A/S-3.png]

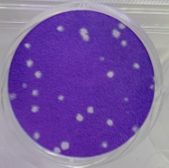

Supplement: Supplementary file 7 — Source data Fig. 2 [file 44319_2024_358_MOESM7_ESM.zip › Source data Figure 2/Figure 2A/si-HDAC6 1#+HSV-1 12h.png]

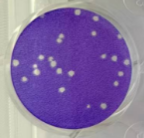

Supplement: Supplementary file 7 — Source data Fig. 2 [file 44319_2024_358_MOESM7_ESM.zip › Source data Figure 2/Figure 2A/si-HDAC6 1#+HSV-1 4h.png]

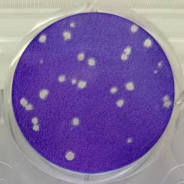

Supplement: Supplementary file 7 — Source data Fig. 2 [file 44319_2024_358_MOESM7_ESM.zip › Source data Figure 2/Figure 2A/si-HDAC6 1#+HSV-1 8h.png]

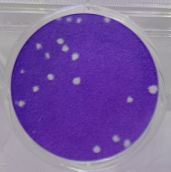

Supplement: Supplementary file 7 — Source data Fig. 2 [file 44319_2024_358_MOESM7_ESM.zip › Source data Figure 2/Figure 2A/si-HDAC6 2#+HSV-1 12h.png]

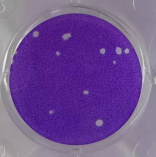

Supplement: Supplementary file 7 — Source data Fig. 2 [file 44319_2024_358_MOESM7_ESM.zip › Source data Figure 2/Figure 2A/si-HDAC6 2#+HSV-1 4h.png]

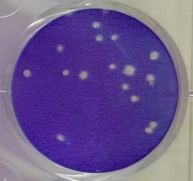

Supplement: Supplementary file 7 — Source data Fig. 2 [file 44319_2024_358_MOESM7_ESM.zip › Source data Figure 2/Figure 2A/si-HDAC6 2#+HSV-1 8h.png]

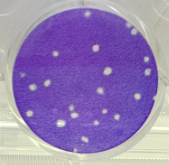

Supplement: Supplementary file 7 — Source data Fig. 2 [file 44319_2024_358_MOESM7_ESM.zip › Source data Figure 2/Figure 2A/si-HDAC6 3#+HSV-1 12h.png]

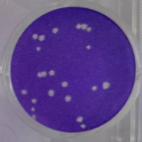

Supplement: Supplementary file 7 — Source data Fig. 2 [file 44319_2024_358_MOESM7_ESM.zip › Source data Figure 2/Figure 2A/si-HDAC6 3#+HSV-1 4h.png]

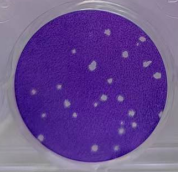

Supplement: Supplementary file 7 — Source data Fig. 2 [file 44319_2024_358_MOESM7_ESM.zip › Source data Figure 2/Figure 2A/si-HDAC6 3#+HSV-1 8h.png]

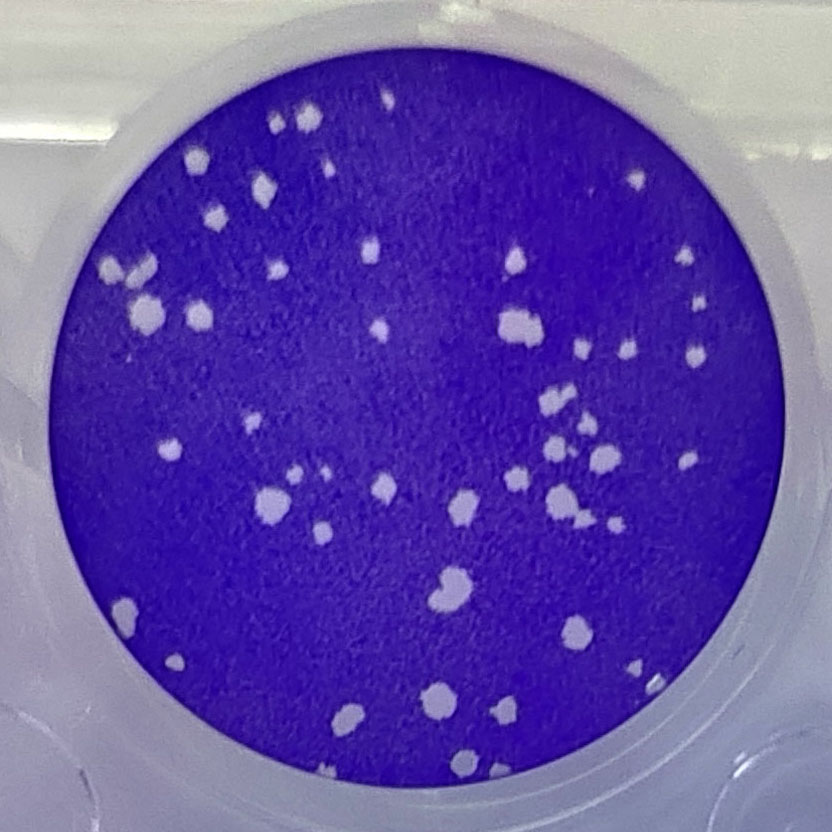

Supplement: Supplementary file 7 — Source data Fig. 2 [file 44319_2024_358_MOESM7_ESM.zip › Source data Figure 2/Figure 2C/HDAC6-WT+HSV-1.jpg]

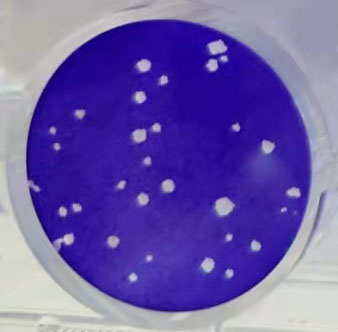

Supplement: Supplementary file 7 — Source data Fig. 2 [file 44319_2024_358_MOESM7_ESM.zip › Source data Figure 2/Figure 2C/vector+HSV-1.jpg]

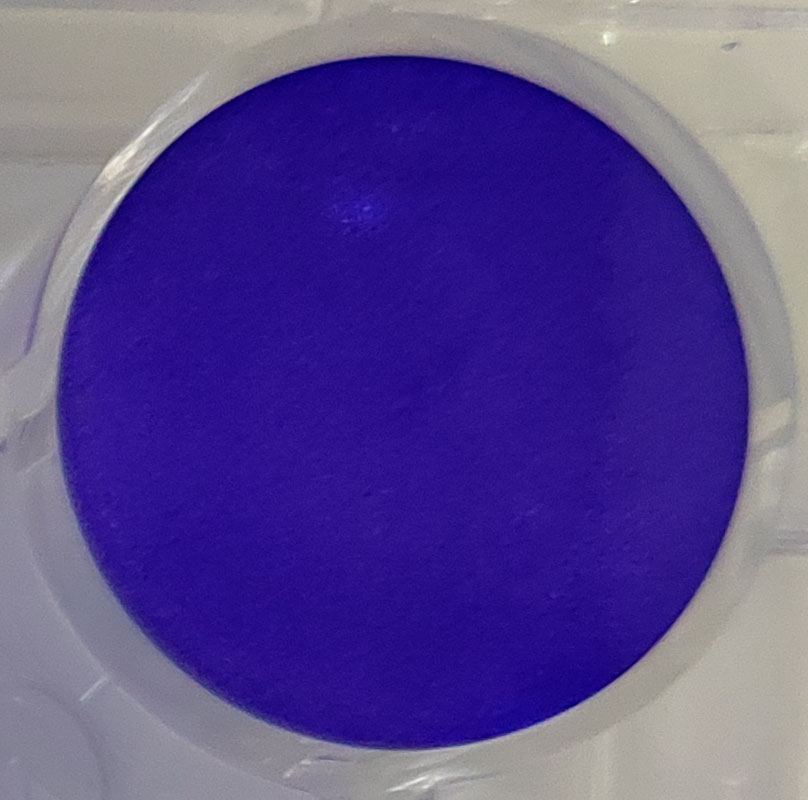

Supplement: Supplementary file 7 — Source data Fig. 2 [file 44319_2024_358_MOESM7_ESM.zip › Source data Figure 2/Figure 2C/vector.jpg]

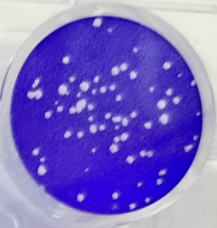

Supplement: Supplementary file 7 — Source data Fig. 2 [file 44319_2024_358_MOESM7_ESM.zip › Source data Figure 2/Figure 2D/HDAC6-WT+HSV-1.png]

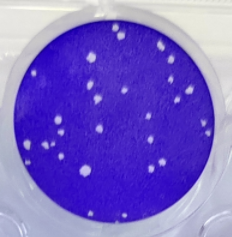

Supplement: Supplementary file 7 — Source data Fig. 2 [file 44319_2024_358_MOESM7_ESM.zip › Source data Figure 2/Figure 2D/mut+HSV-1.png]

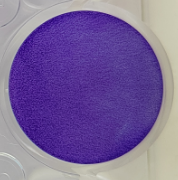

Supplement: Supplementary file 7 — Source data Fig. 2 [file 44319_2024_358_MOESM7_ESM.zip › Source data Figure 2/Figure 2F/cell.png]

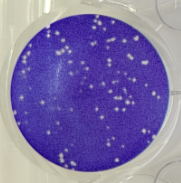

Supplement: Supplementary file 7 — Source data Fig. 2 [file 44319_2024_358_MOESM7_ESM.zip › Source data Figure 2/Figure 2F/H+T.png]

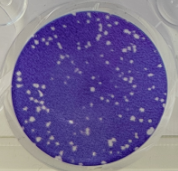

Supplement: Supplementary file 7 — Source data Fig. 2 [file 44319_2024_358_MOESM7_ESM.zip › Source data Figure 2/Figure 2F/HSV-1.png]

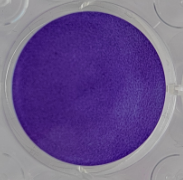

Supplement: Supplementary file 7 — Source data Fig. 2 [file 44319_2024_358_MOESM7_ESM.zip › Source data Figure 2/Figure 2F/Tubacin.png]

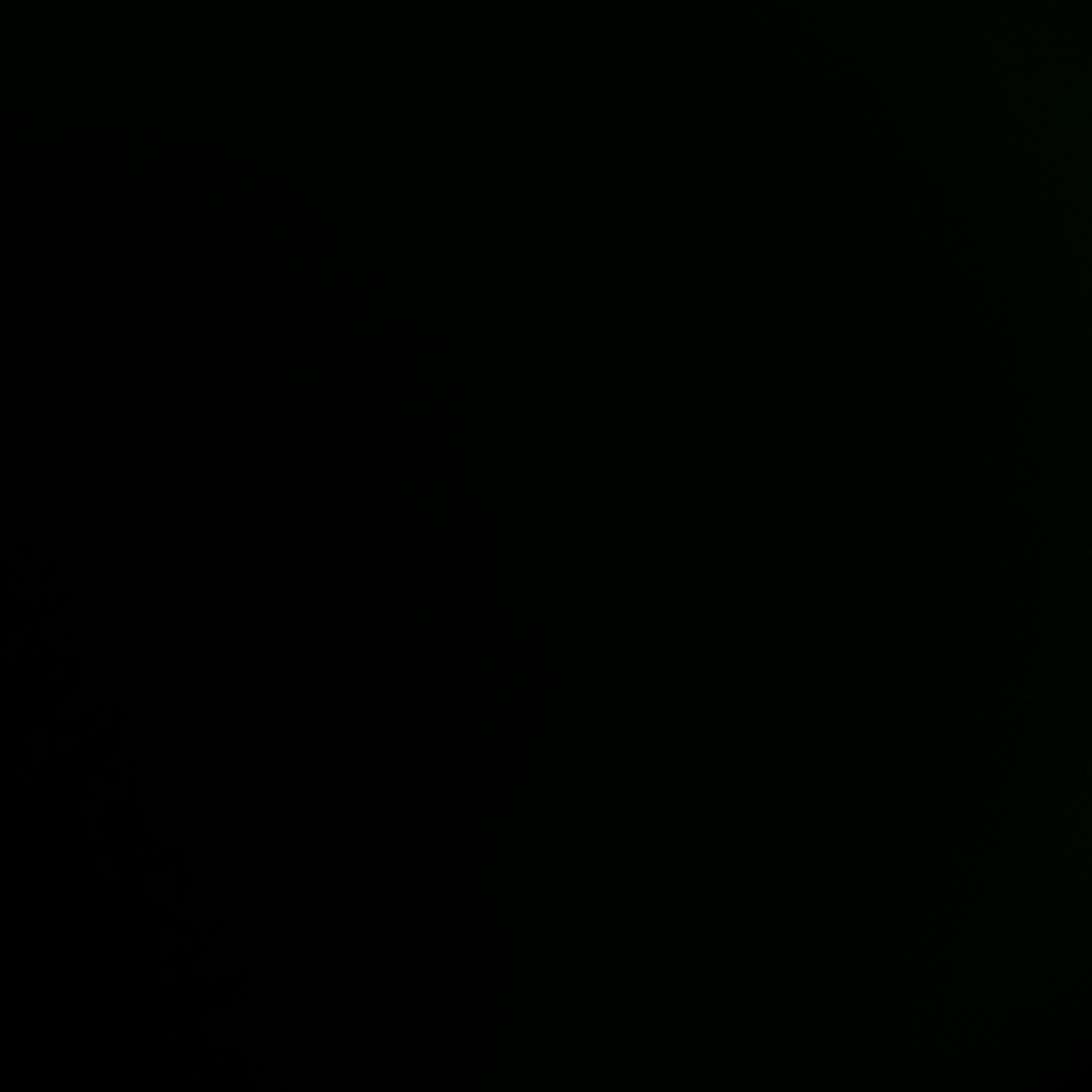

Supplement: Supplementary file 7 — Source data Fig. 2 [file 44319_2024_358_MOESM7_ESM.zip › Source data Figure 2/Figure 2G/EGFP-Cell.tif]

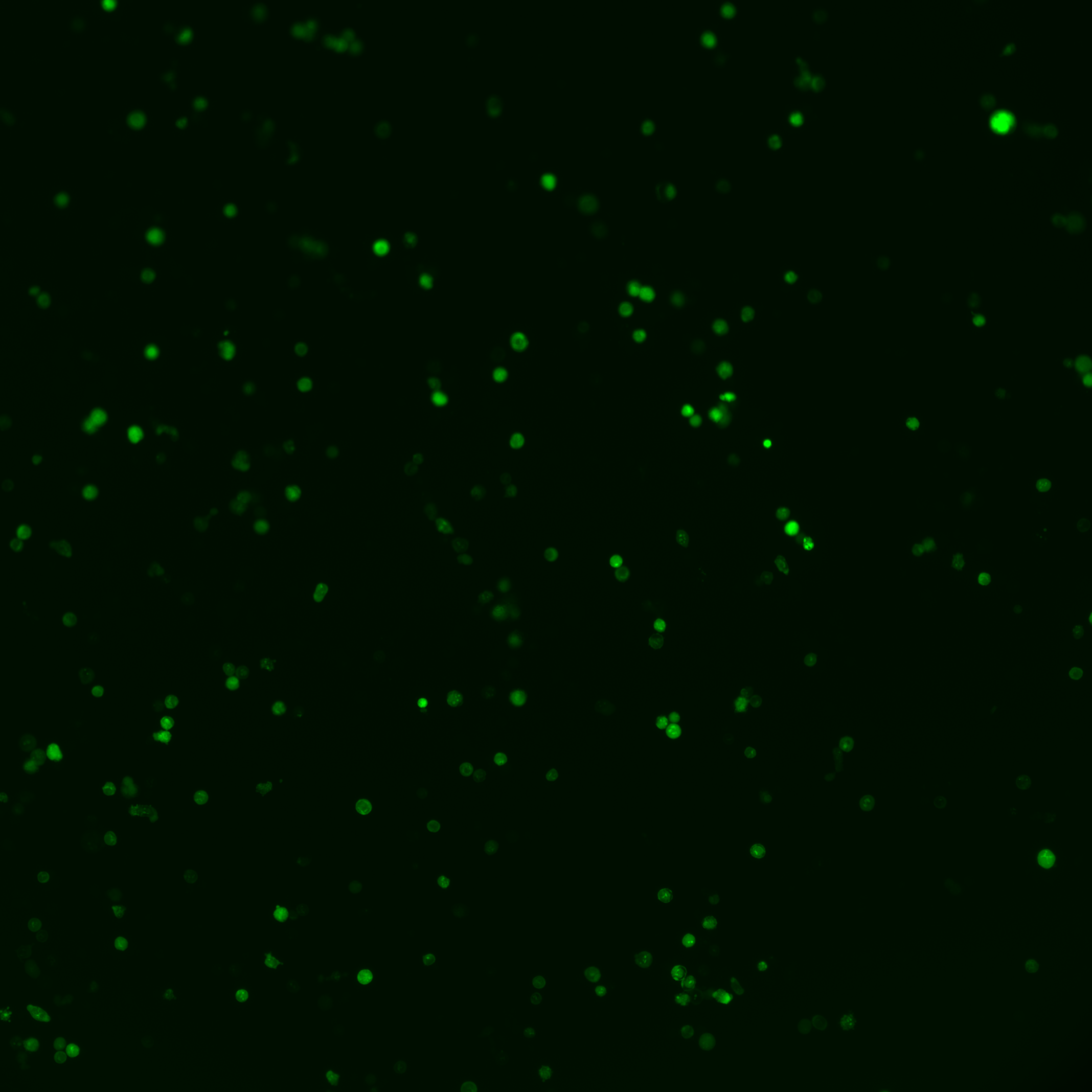

Supplement: Supplementary file 7 — Source data Fig. 2 [file 44319_2024_358_MOESM7_ESM.zip › Source data Figure 2/Figure 2G/EGPF-HSV-1+Tubacin.tif]

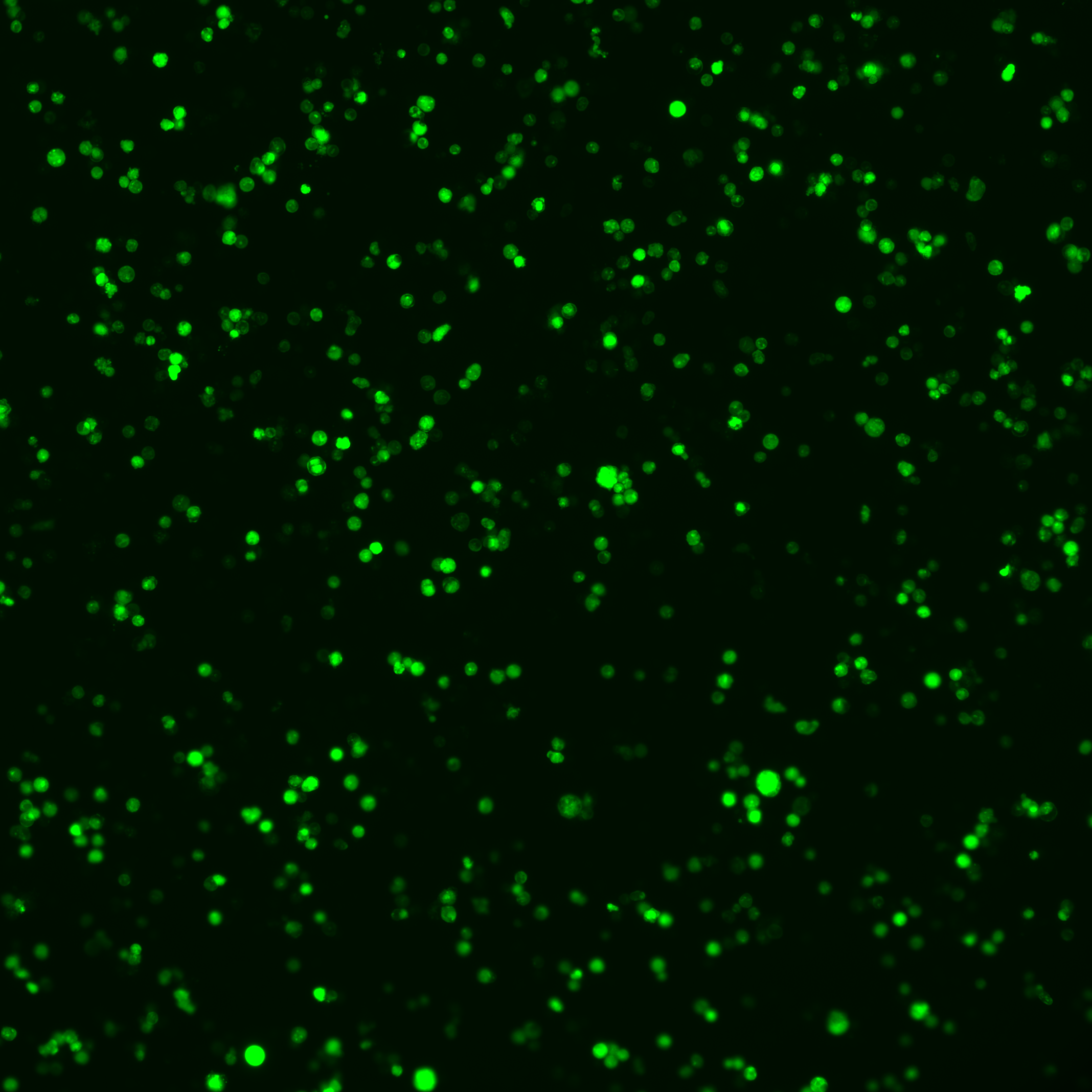

Supplement: Supplementary file 7 — Source data Fig. 2 [file 44319_2024_358_MOESM7_ESM.zip › Source data Figure 2/Figure 2G/EGPF-HSV-1.tif]

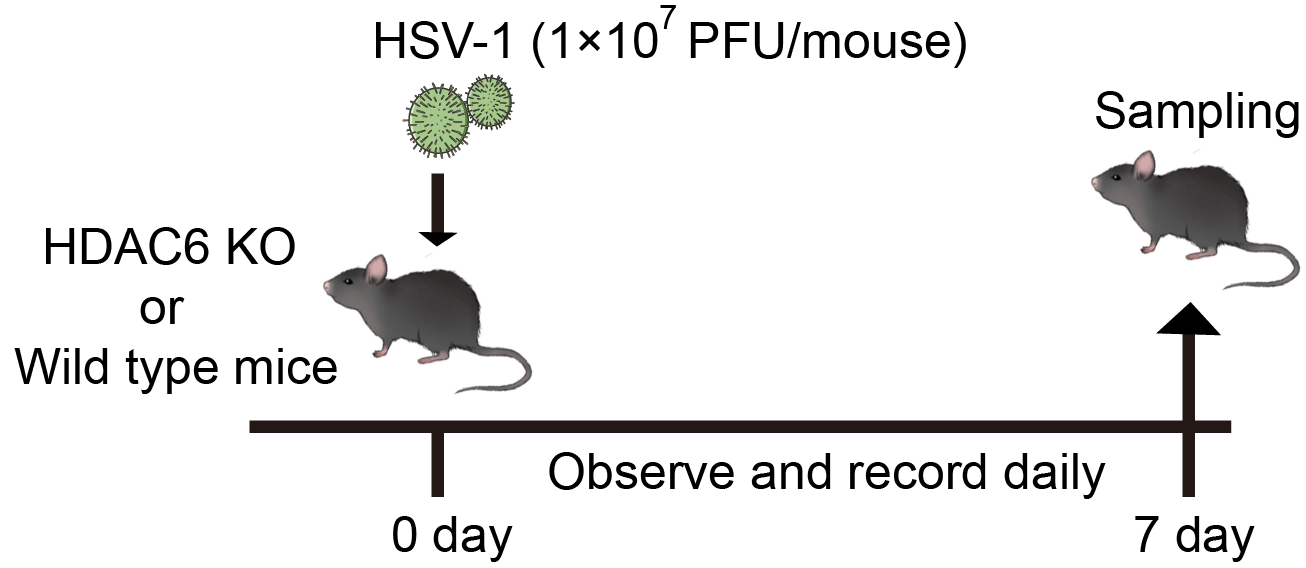

Supplement: Supplementary file 8 — Source data Fig. 3 [file 44319_2024_358_MOESM8_ESM.zip › Source data Figure 3/Figure 3A.tif]

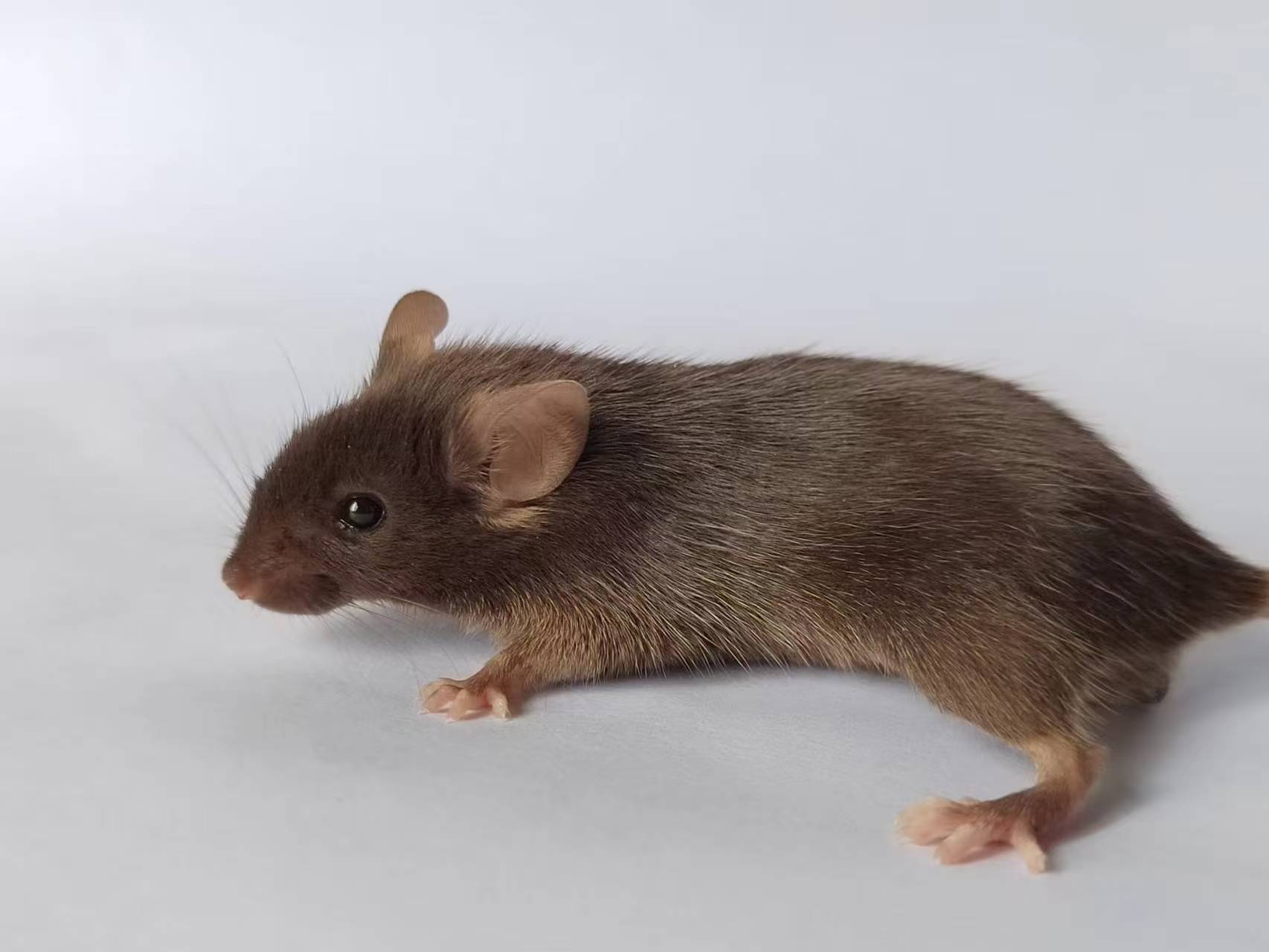

Supplement: Supplementary file 8 — Source data Fig. 3 [file 44319_2024_358_MOESM8_ESM.zip › Source data Figure 3/Figure 3B/HDAC6 KO+HSV-1 5 d.p.i.jpg]

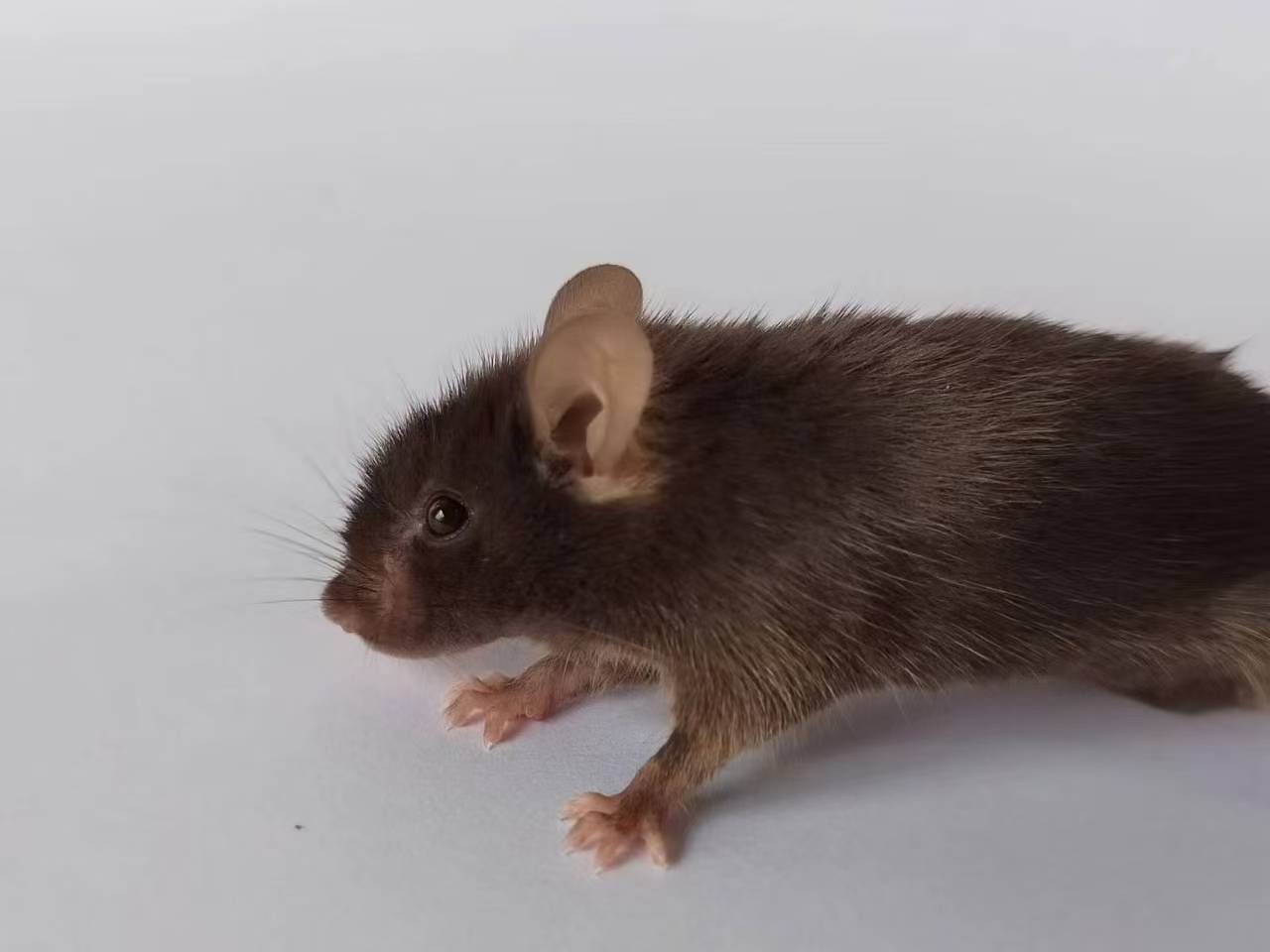

Supplement: Supplementary file 8 — Source data Fig. 3 [file 44319_2024_358_MOESM8_ESM.zip › Source data Figure 3/Figure 3B/HDAC6 KO+HSV-1 6 d.p.i.jpg]

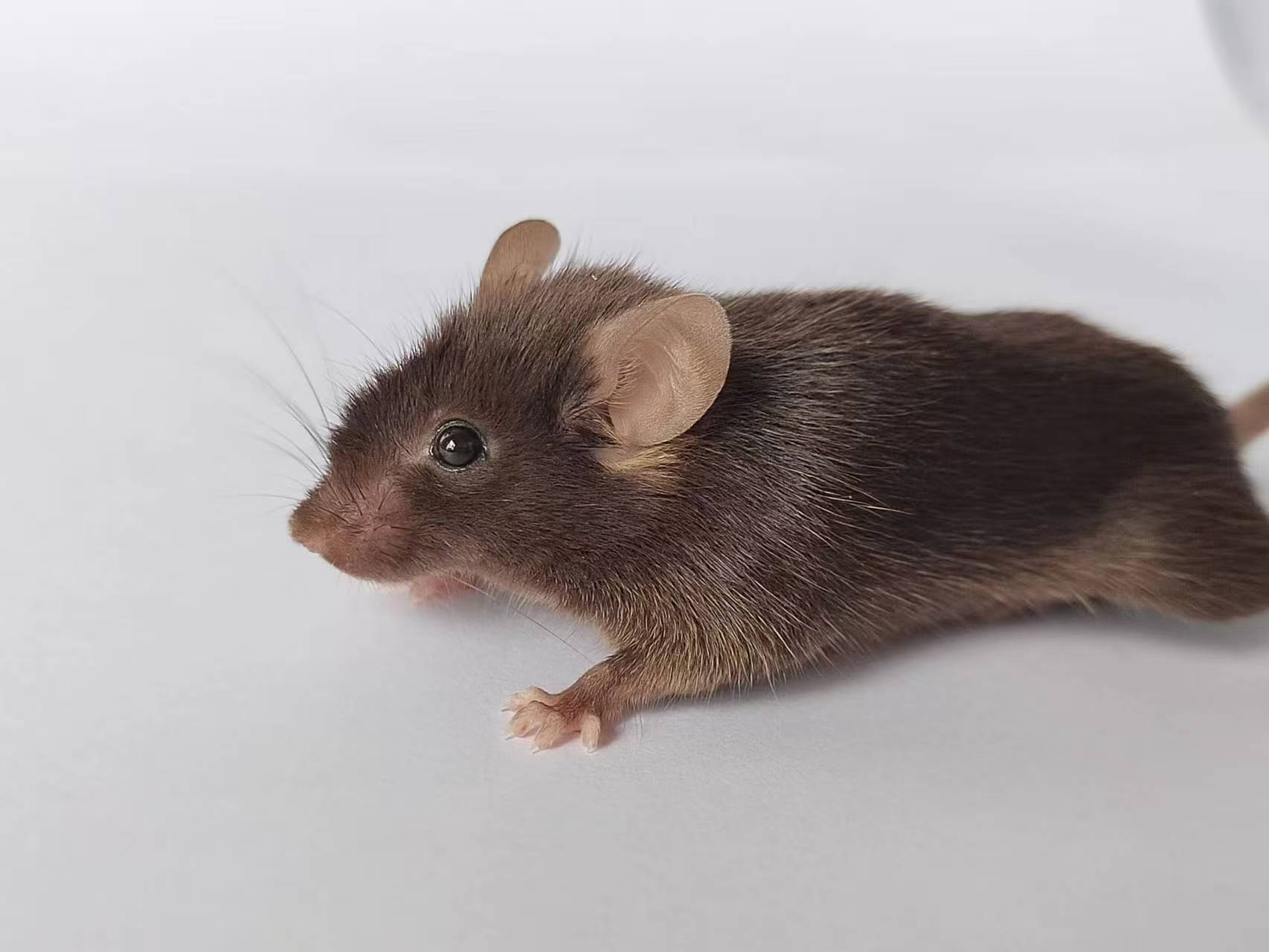

Supplement: Supplementary file 8 — Source data Fig. 3 [file 44319_2024_358_MOESM8_ESM.zip › Source data Figure 3/Figure 3B/HDAC6 KO+HSV-1 7 d.p.i.jpg]

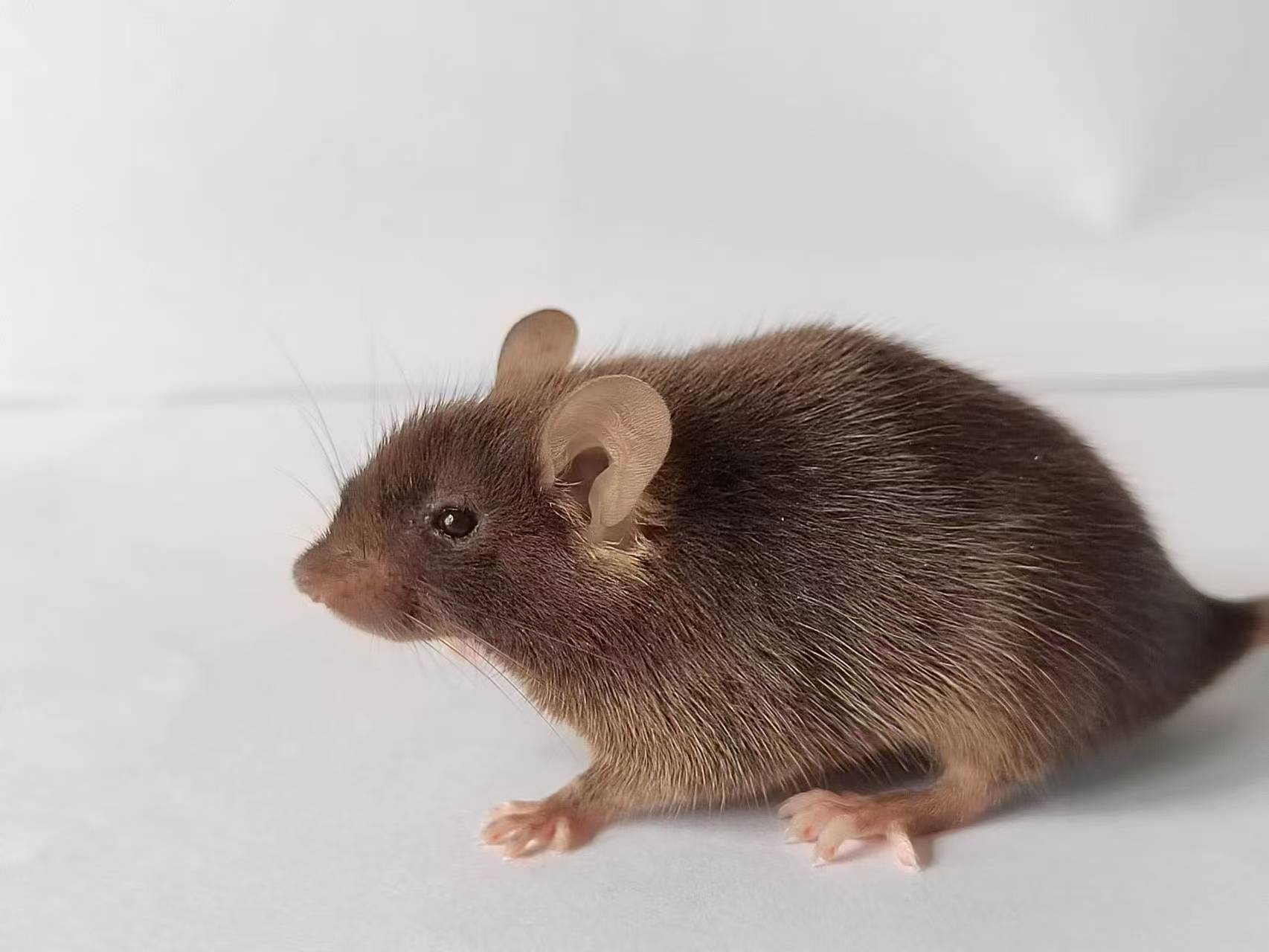

Supplement: Supplementary file 8 — Source data Fig. 3 [file 44319_2024_358_MOESM8_ESM.zip › Source data Figure 3/Figure 3B/WT+HSV-1 5 d.p.i.jpg]

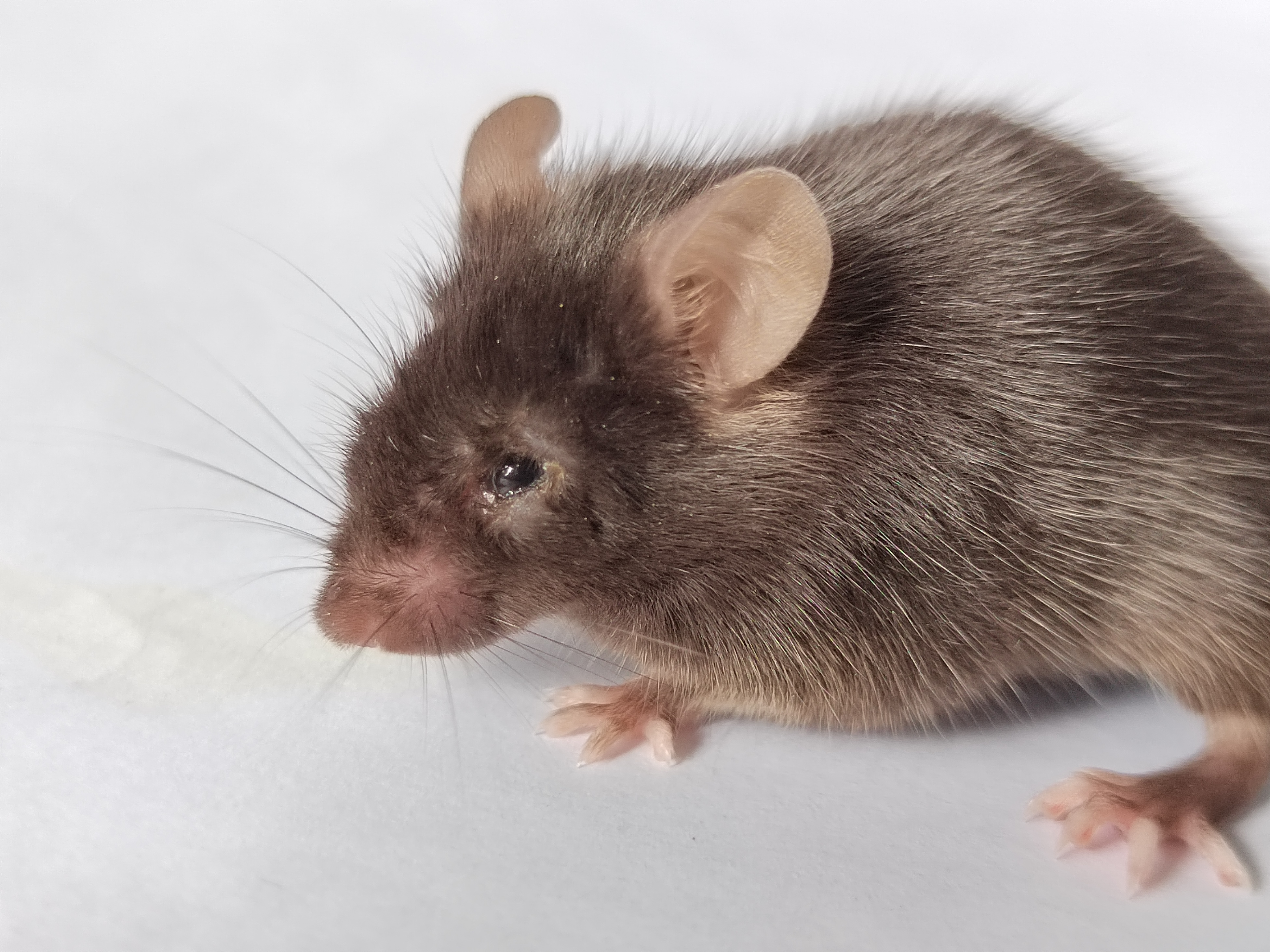

Supplement: Supplementary file 8 — Source data Fig. 3 [file 44319_2024_358_MOESM8_ESM.zip › Source data Figure 3/Figure 3B/WT+HSV-1 6 d.p.i.jpg]

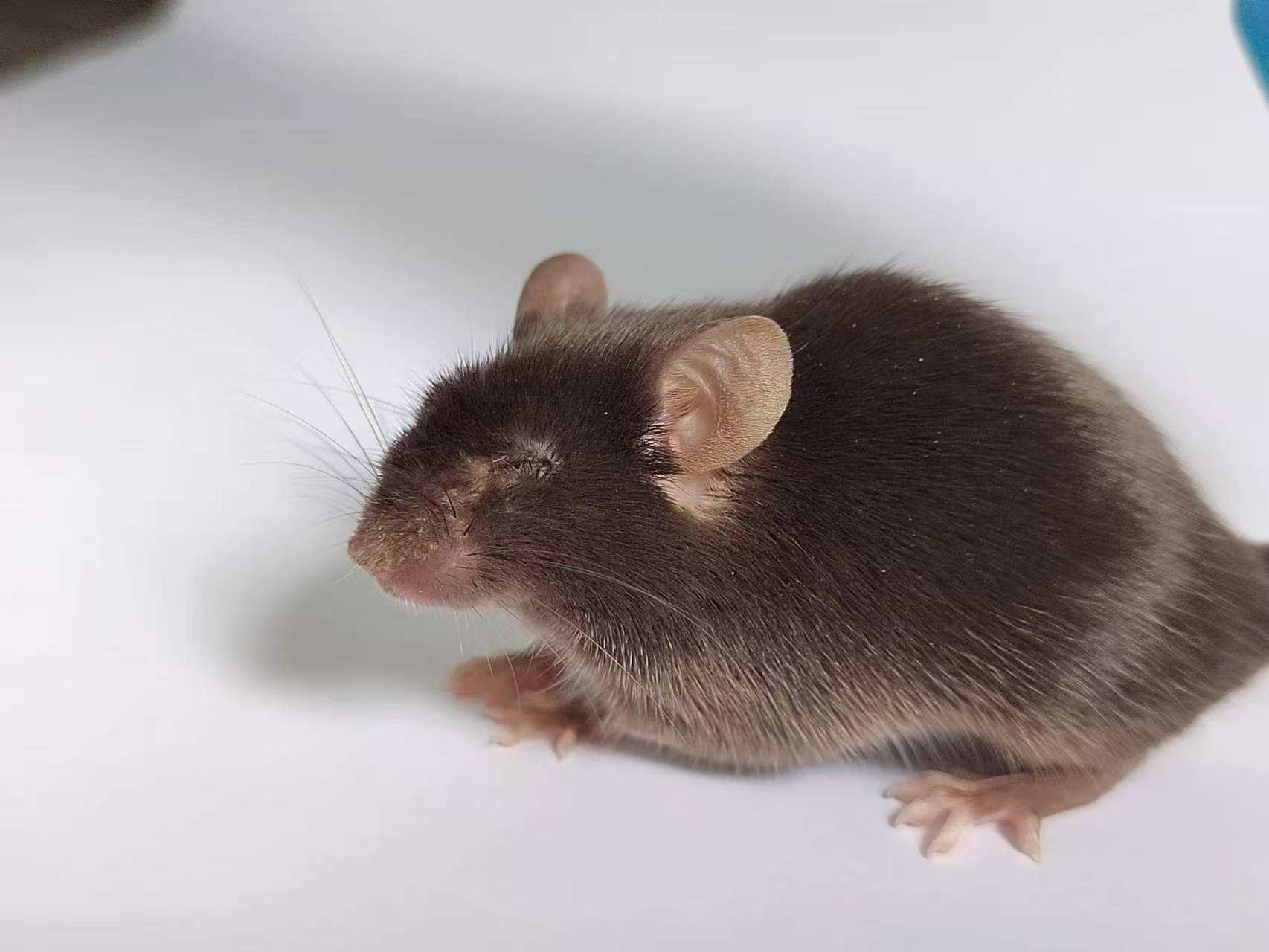

Supplement: Supplementary file 8 — Source data Fig. 3 [file 44319_2024_358_MOESM8_ESM.zip › Source data Figure 3/Figure 3B/WT+HSV-1 7 d.p.i.jpg]

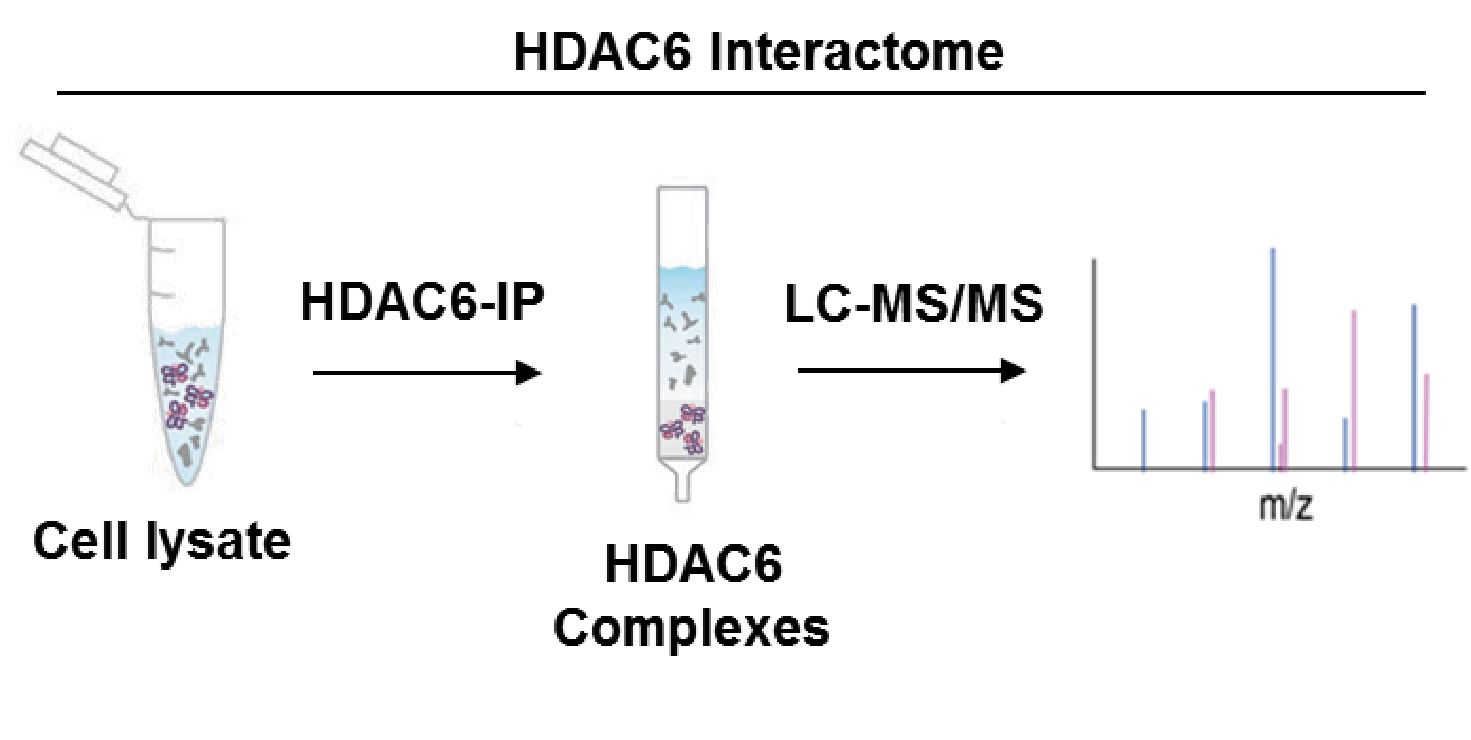

Supplement: Supplementary file 9 — Source data Fig. 4 [file 44319_2024_358_MOESM9_ESM.zip › Source data Figure 4/Figure 4A.tif]

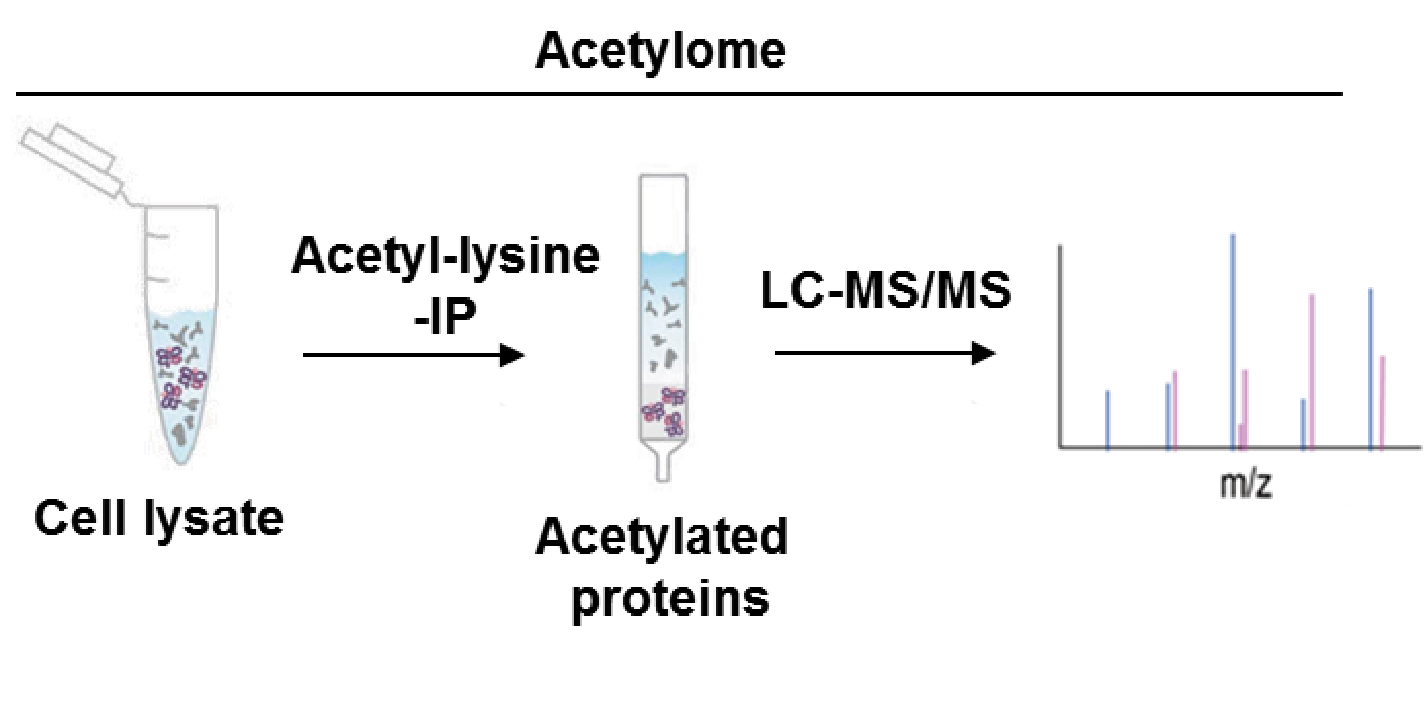

Supplement: Supplementary file 9 — Source data Fig. 4 [file 44319_2024_358_MOESM9_ESM.zip › Source data Figure 4/Figure 4C.tif]

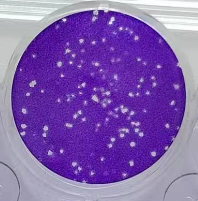

Supplement: Supplementary file 10 — Source data Fig. 5 [file 44319_2024_358_MOESM10_ESM.zip › Source data Figure 5/Figure 5J/B-box-A+HSV-1.png]

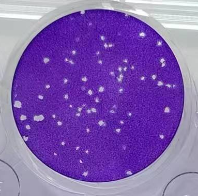

Supplement: Supplementary file 10 — Source data Fig. 5 [file 44319_2024_358_MOESM10_ESM.zip › Source data Figure 5/Figure 5J/B-box-WT+HSV-1.png]

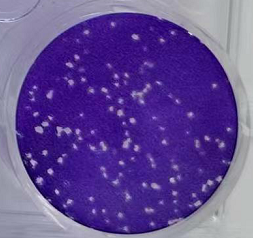

Supplement: Supplementary file 10 — Source data Fig. 5 [file 44319_2024_358_MOESM10_ESM.zip › Source data Figure 5/Figure 5J/vector+HSV-1.png]

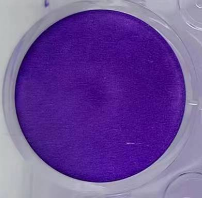

Supplement: Supplementary file 10 — Source data Fig. 5 [file 44319_2024_358_MOESM10_ESM.zip › Source data Figure 5/Figure 5J/vector.png]

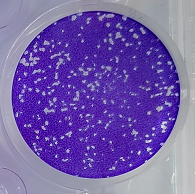

Supplement: Supplementary file 11 — Source data Fig. 6 [file 44319_2024_358_MOESM11_ESM.zip › Source data Figure 6/Figure 6H/3.1.png]

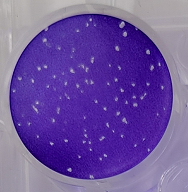

Supplement: Supplementary file 11 — Source data Fig. 6 [file 44319_2024_358_MOESM11_ESM.zip › Source data Figure 6/Figure 6H/TRIM56.png]

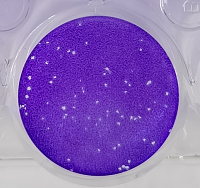

Supplement: Supplementary file 11 — Source data Fig. 6 [file 44319_2024_358_MOESM11_ESM.zip › Source data Figure 6/Figure 6H/TRIM56Q.png]

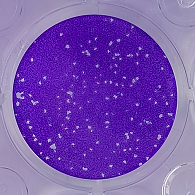

Supplement: Supplementary file 11 — Source data Fig. 6 [file 44319_2024_358_MOESM11_ESM.zip › Source data Figure 6/Figure 6H/TRIM56R.png]

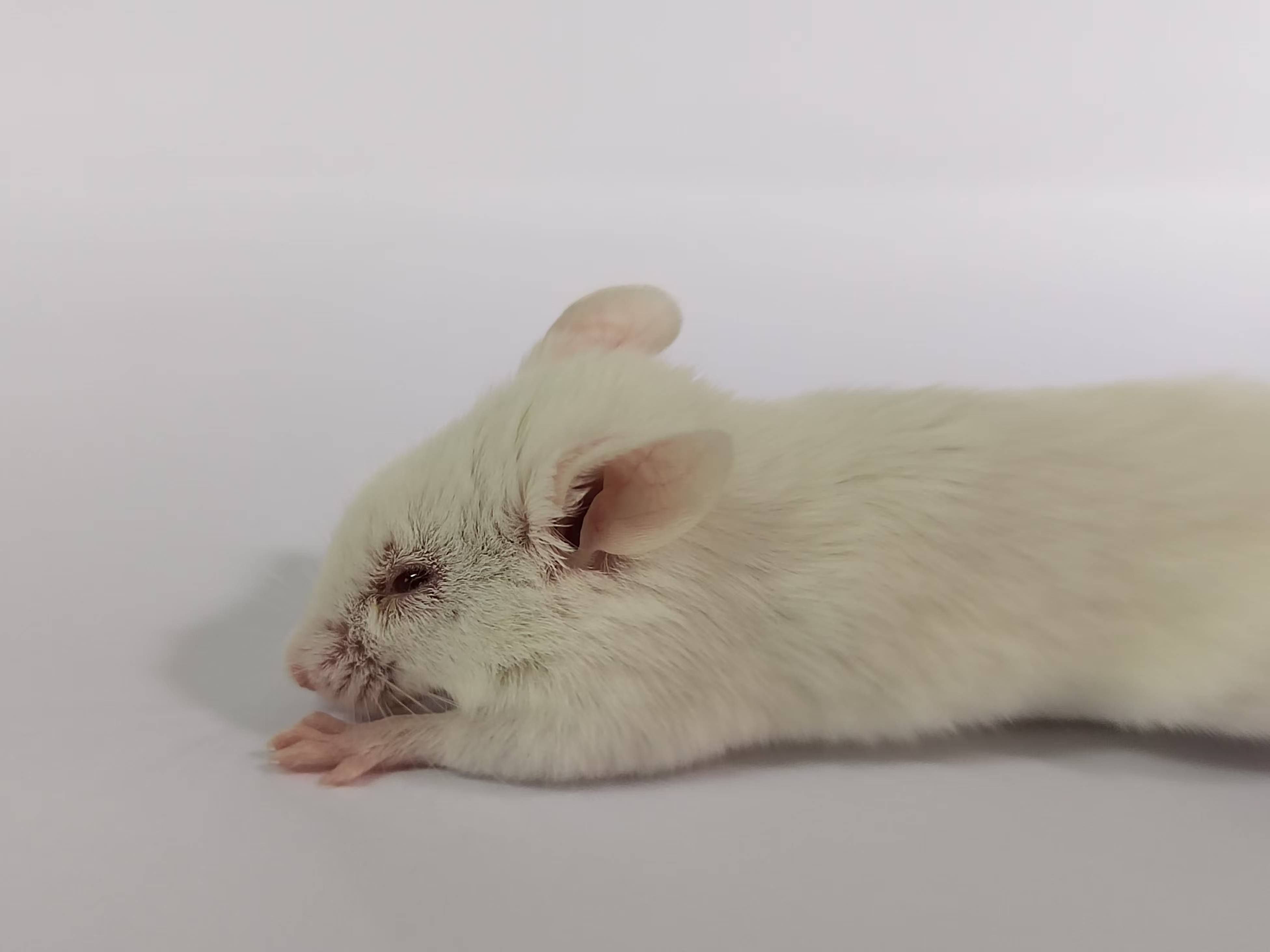

Supplement: Supplementary file 12 — Source data Fig. 7 [file 44319_2024_358_MOESM12_ESM.zip › Source data Figure 7/7C/LV-Ctrl 5d.p.i.jpg]

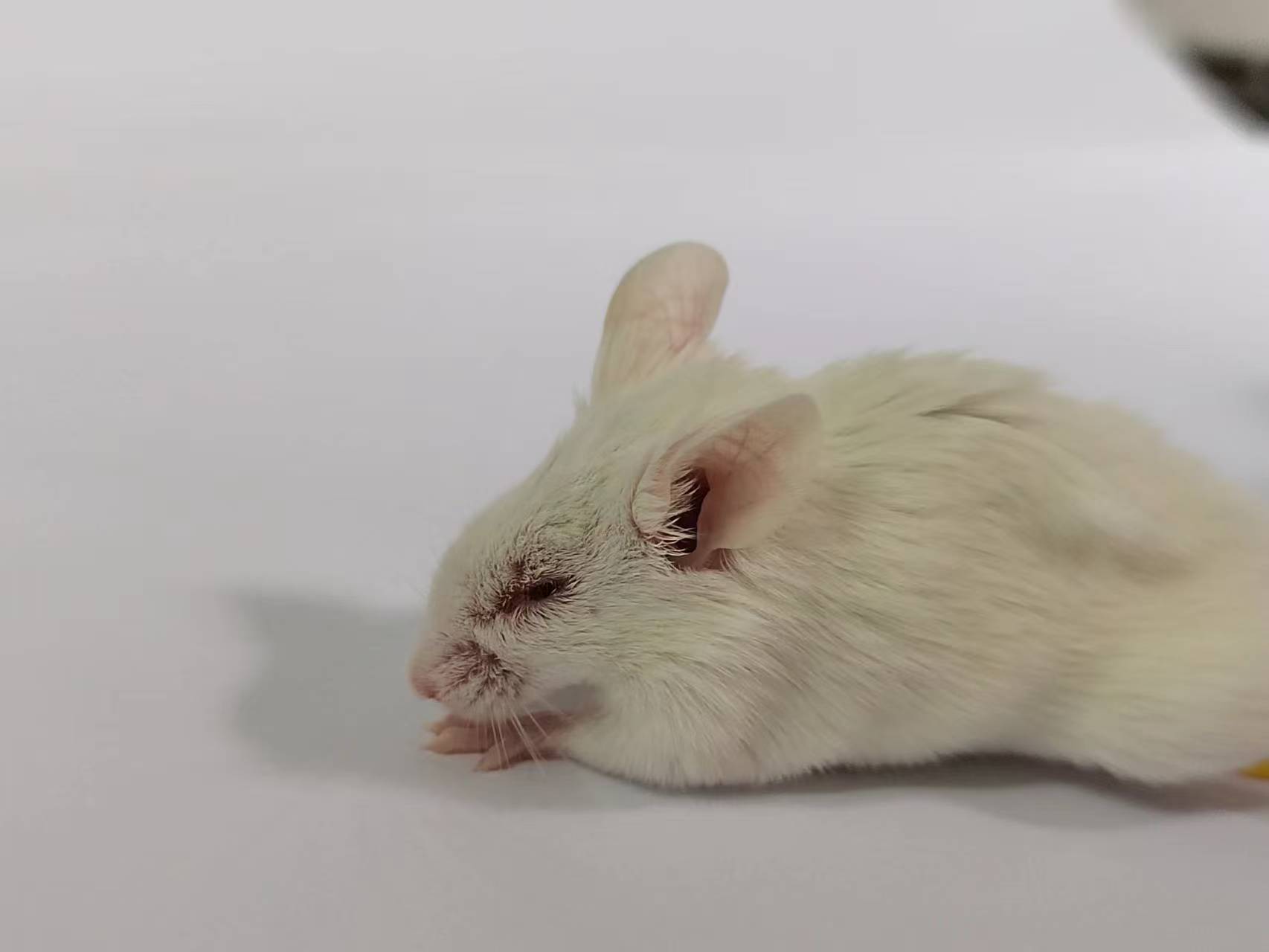

Supplement: Supplementary file 12 — Source data Fig. 7 [file 44319_2024_358_MOESM12_ESM.zip › Source data Figure 7/7C/LV-Ctrl 6d.p.i.jpg]

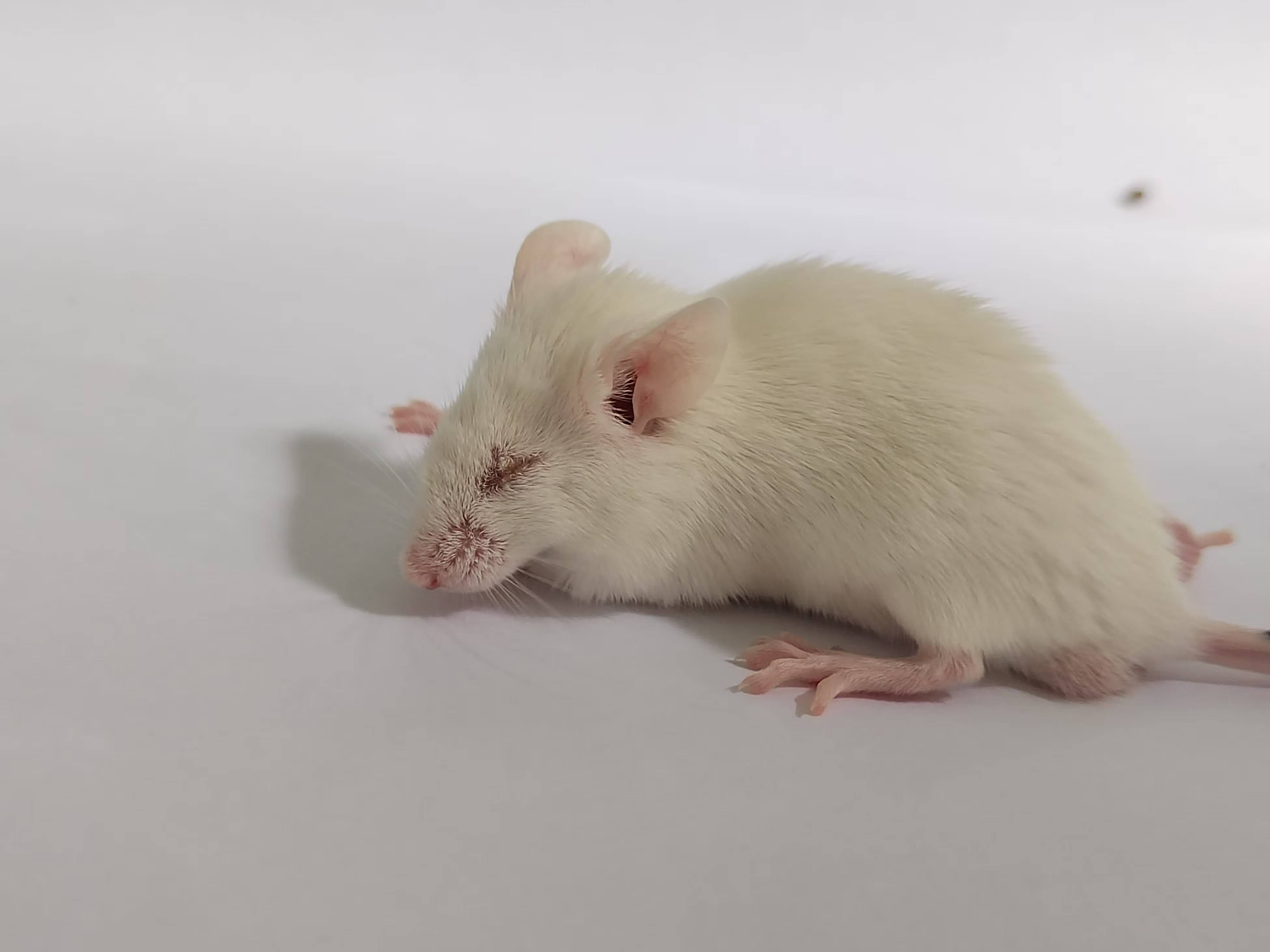

Supplement: Supplementary file 12 — Source data Fig. 7 [file 44319_2024_358_MOESM12_ESM.zip › Source data Figure 7/7C/LV-Ctrl 7d.p.i.jpg]

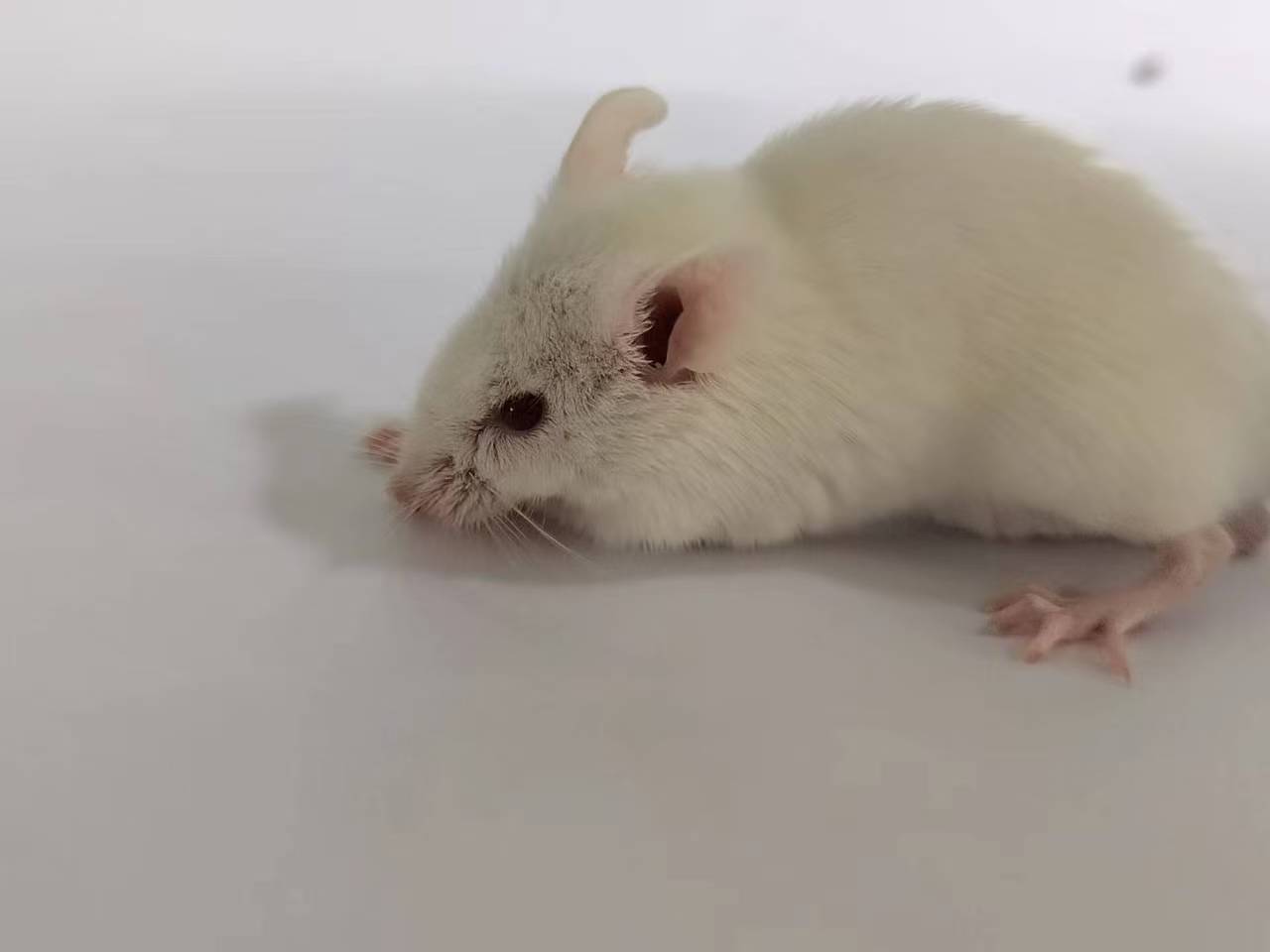

Supplement: Supplementary file 12 — Source data Fig. 7 [file 44319_2024_358_MOESM12_ESM.zip › Source data Figure 7/7C/LV-TRIM56 5d.p.i.jpg]

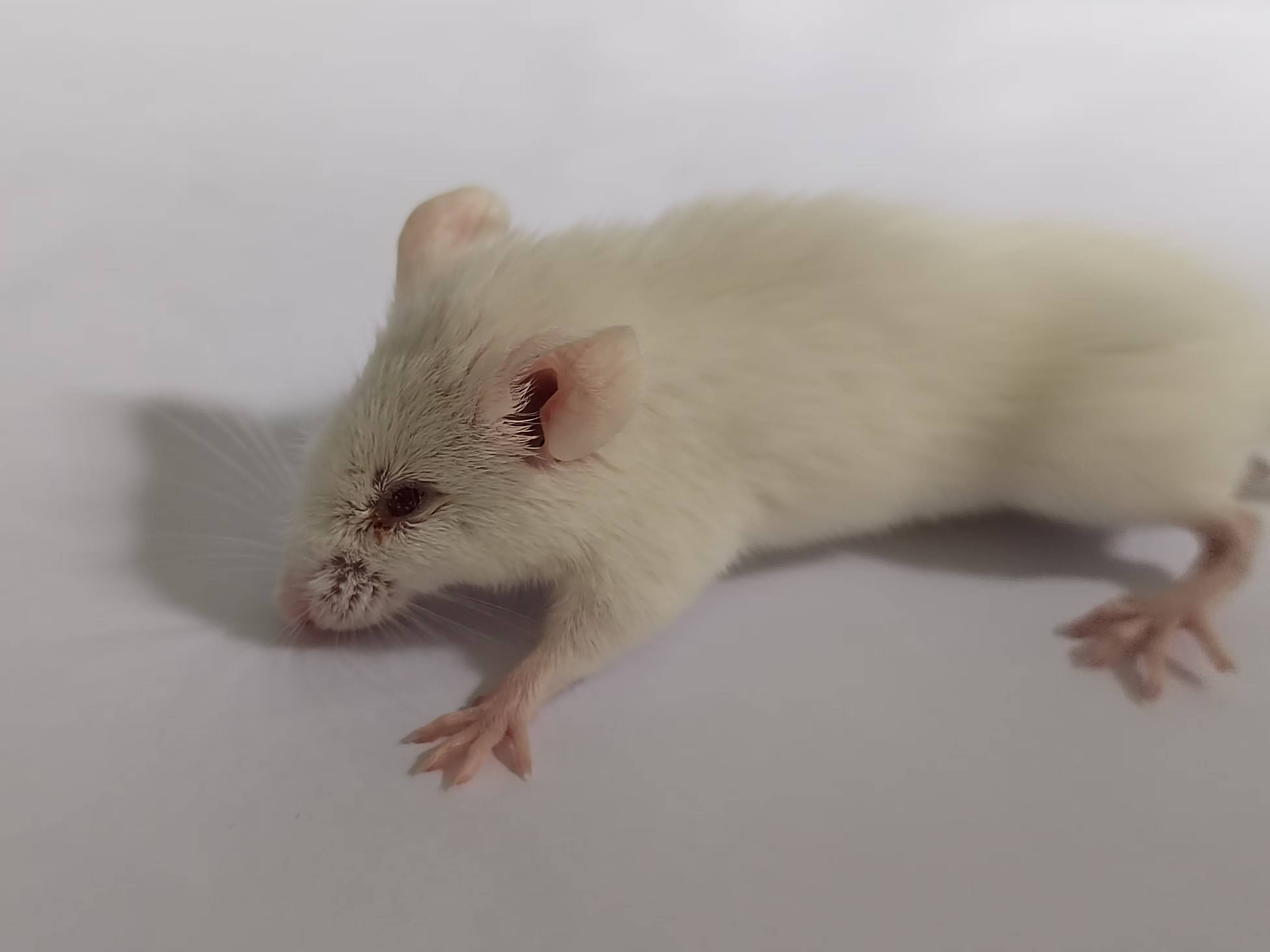

Supplement: Supplementary file 12 — Source data Fig. 7 [file 44319_2024_358_MOESM12_ESM.zip › Source data Figure 7/7C/LV-TRIM56 6d.p.i.jpg]

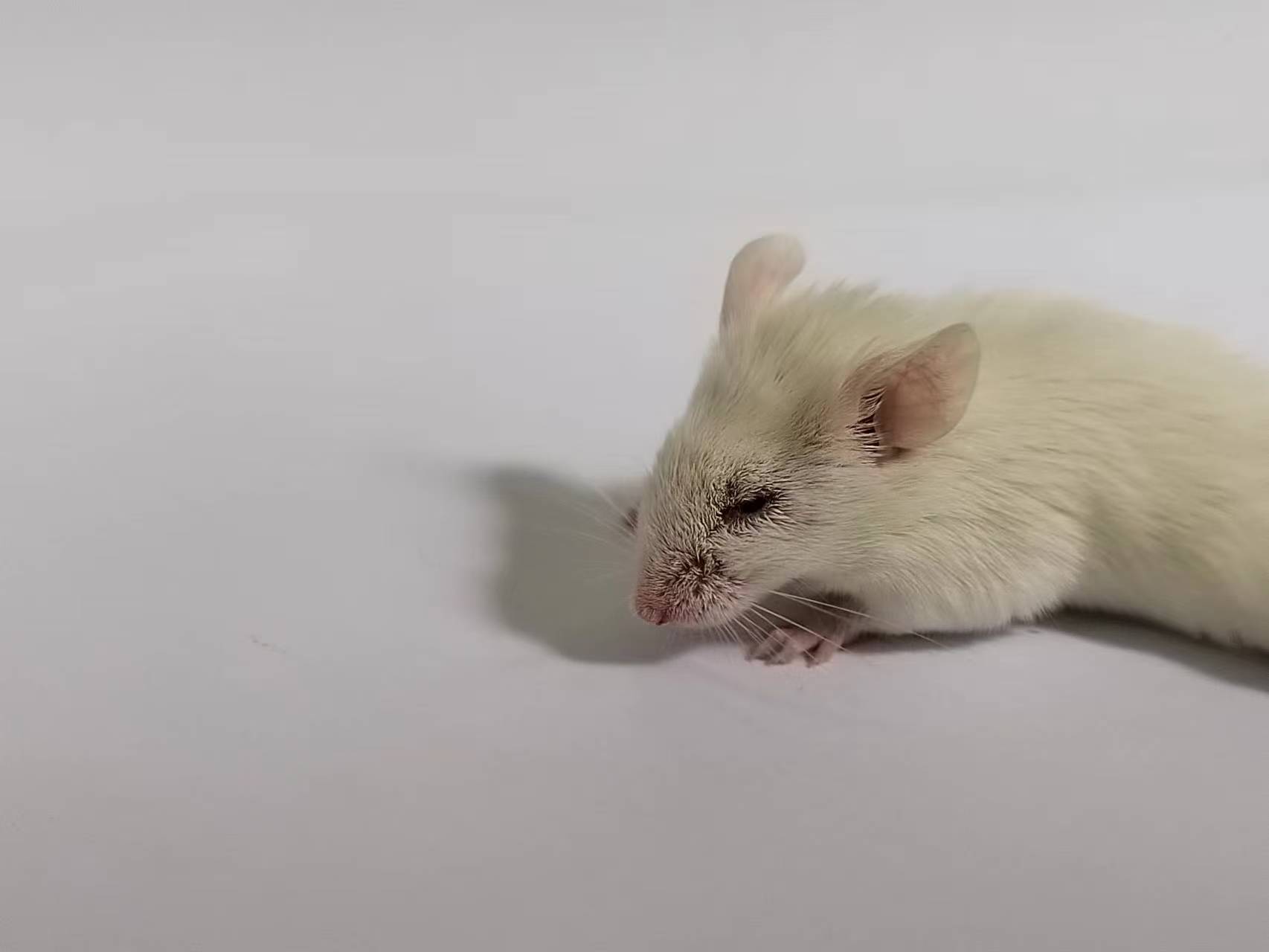

Supplement: Supplementary file 12 — Source data Fig. 7 [file 44319_2024_358_MOESM12_ESM.zip › Source data Figure 7/7C/LV-TRIM56 7d.p.i.jpg]

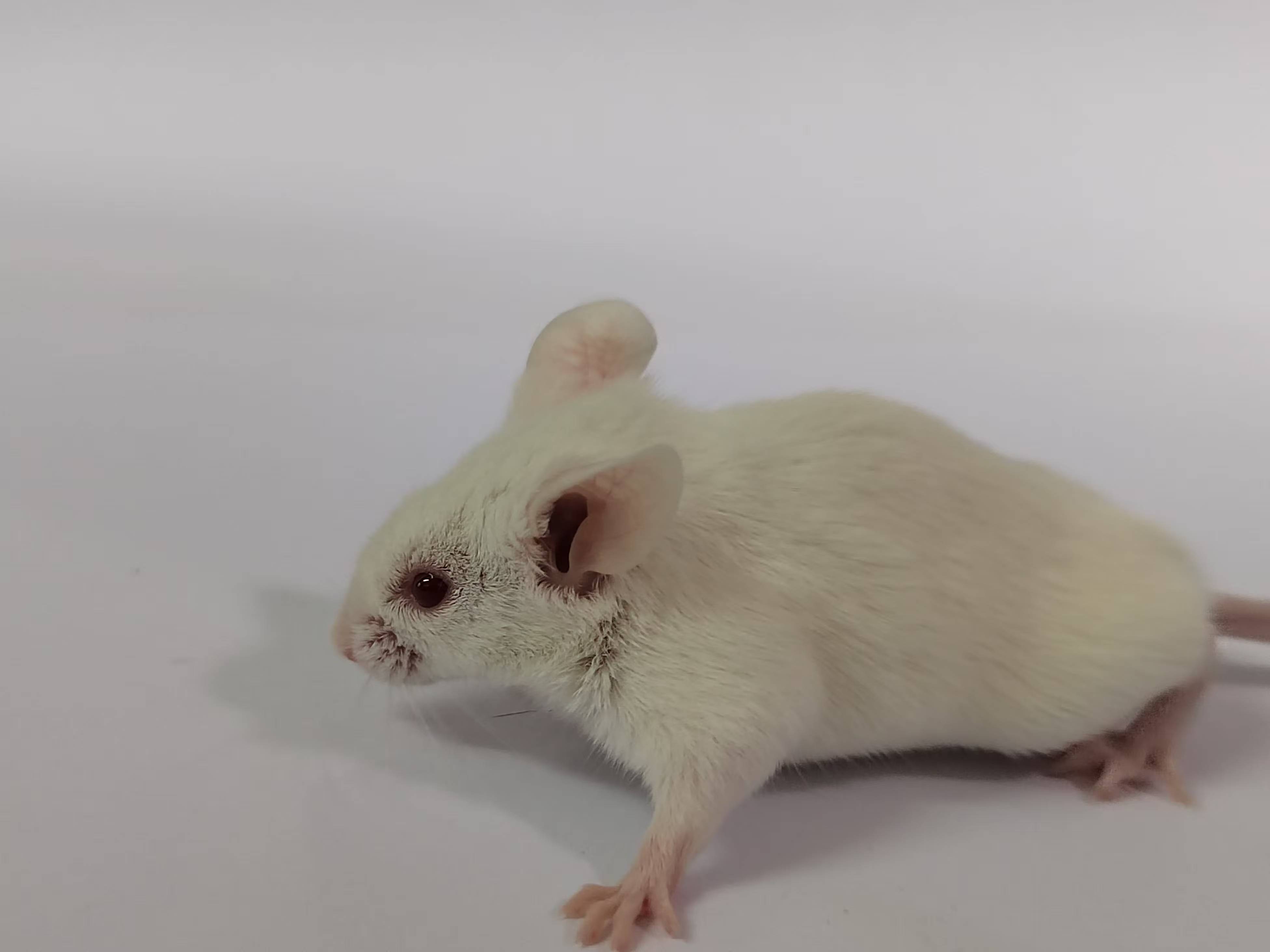

Supplement: Supplementary file 12 — Source data Fig. 7 [file 44319_2024_358_MOESM12_ESM.zip › Source data Figure 7/7C/LV-TRIM56 K110Q 5d.p.i.jpg]

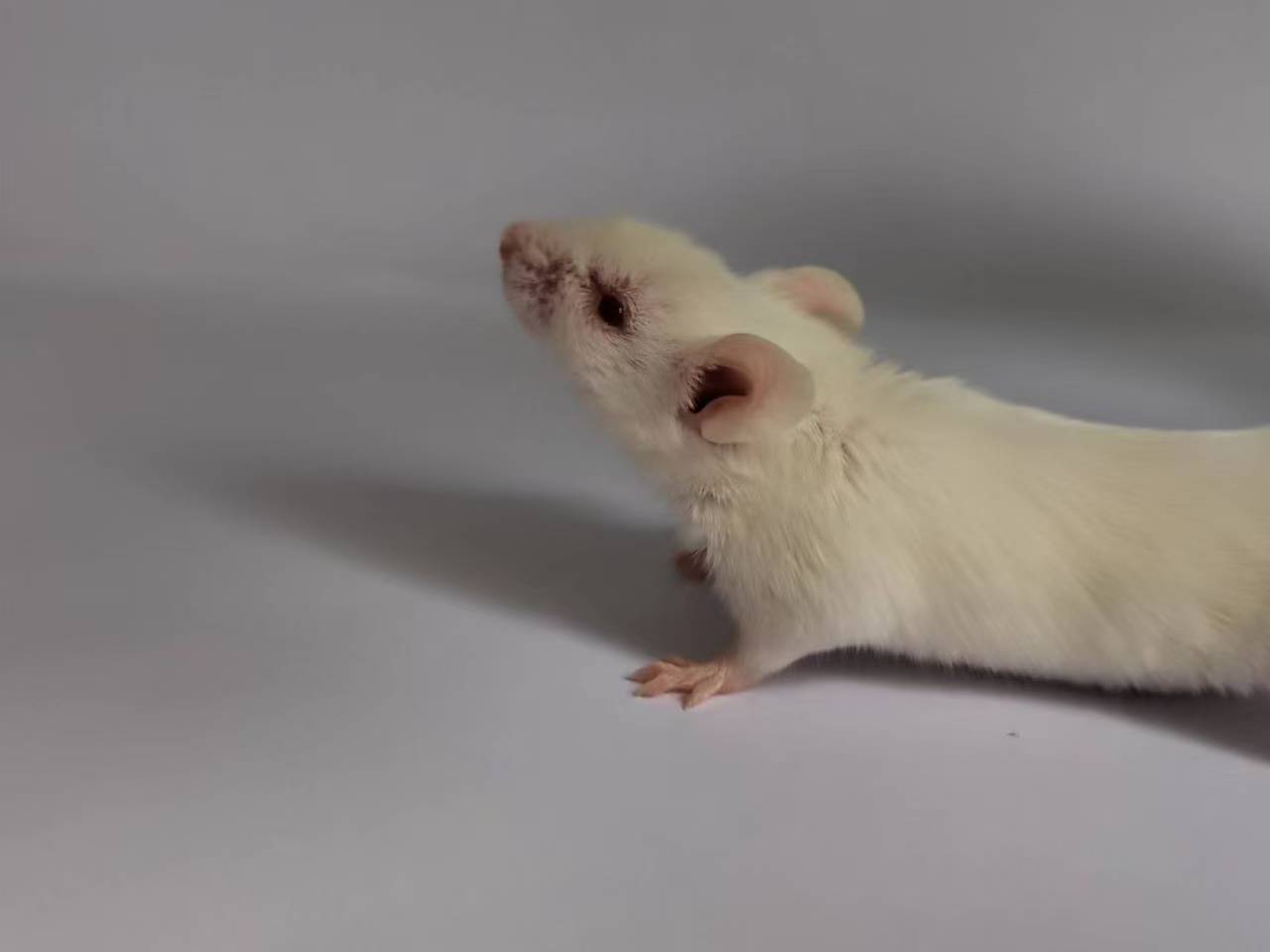

Supplement: Supplementary file 12 — Source data Fig. 7 [file 44319_2024_358_MOESM12_ESM.zip › Source data Figure 7/7C/LV-TRIM56 K110Q 6d.p.i.jpg]

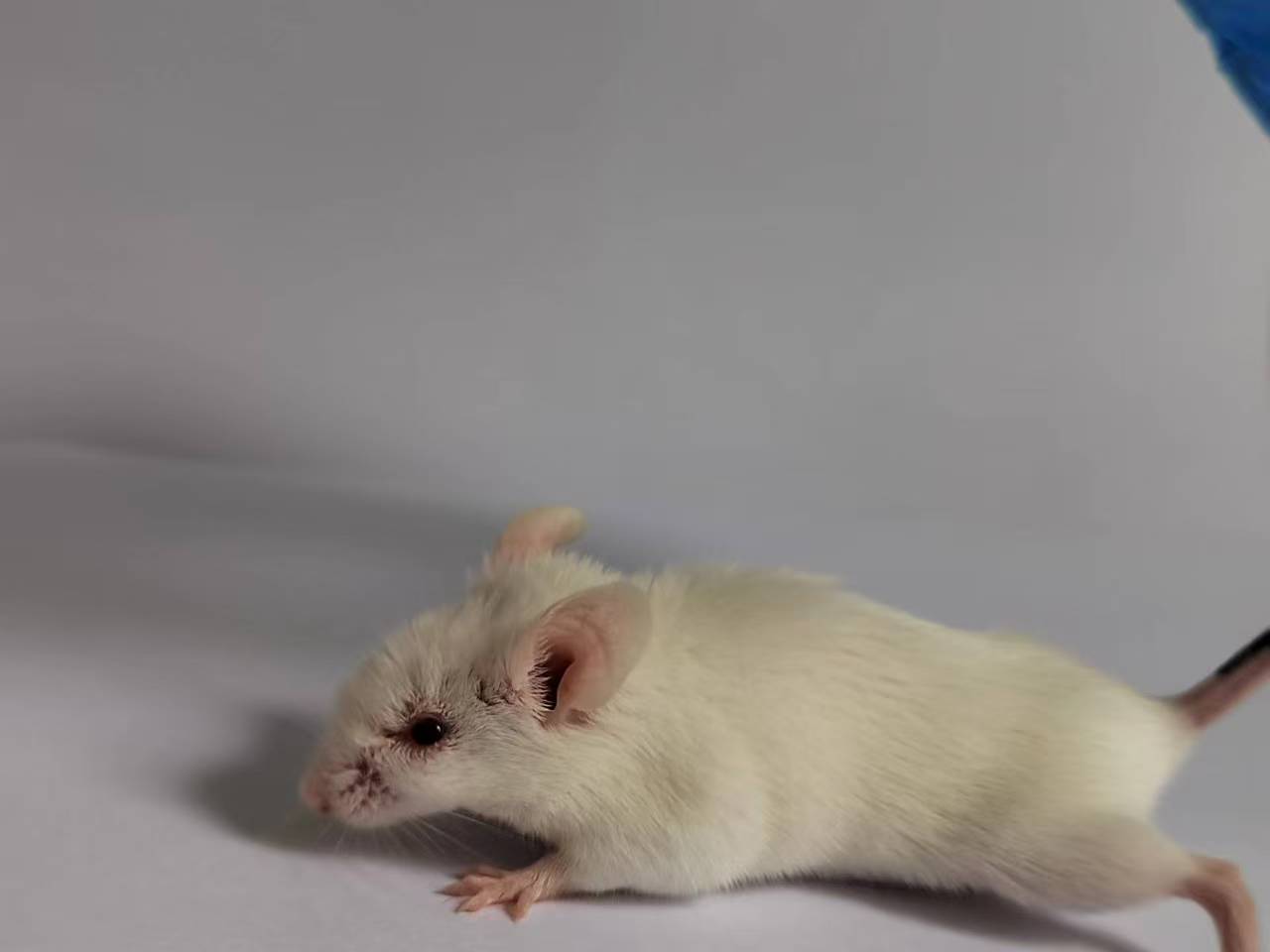

Supplement: Supplementary file 12 — Source data Fig. 7 [file 44319_2024_358_MOESM12_ESM.zip › Source data Figure 7/7C/LV-TRIM56 K110Q 7d.p.i.jpg]

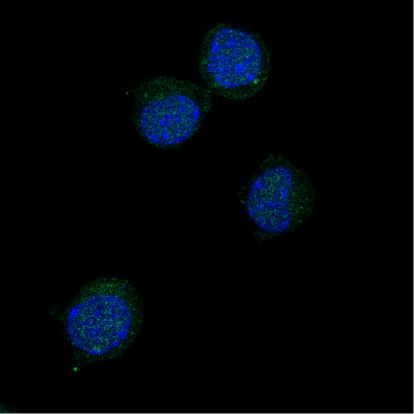

Supplement: Supplementary file 13 — Source data Fig. 8 [file 44319_2024_358_MOESM13_ESM.zip › Source data Figure 8/Figure 8B/0 h.tif]

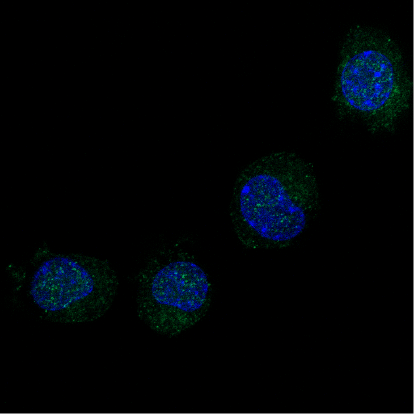

Supplement: Supplementary file 13 — Source data Fig. 8 [file 44319_2024_358_MOESM13_ESM.zip › Source data Figure 8/Figure 8B/2 h.tif]

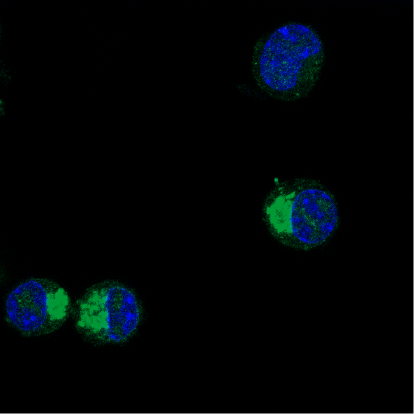

Supplement: Supplementary file 13 — Source data Fig. 8 [file 44319_2024_358_MOESM13_ESM.zip › Source data Figure 8/Figure 8B/4 h.tif]

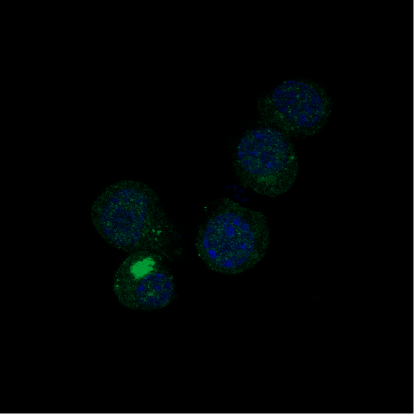

Supplement: Supplementary file 13 — Source data Fig. 8 [file 44319_2024_358_MOESM13_ESM.zip › Source data Figure 8/Figure 8B/6 h.tif]

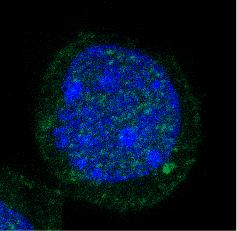

Supplement: Supplementary file 13 — Source data Fig. 8 [file 44319_2024_358_MOESM13_ESM.zip › Source data Figure 8/Figure 8B/inset 0h.tif]

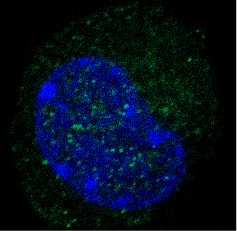

Supplement: Supplementary file 13 — Source data Fig. 8 [file 44319_2024_358_MOESM13_ESM.zip › Source data Figure 8/Figure 8B/inset 2h.tif]

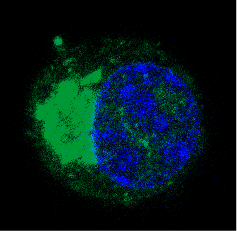

Supplement: Supplementary file 13 — Source data Fig. 8 [file 44319_2024_358_MOESM13_ESM.zip › Source data Figure 8/Figure 8B/inset 4h.tif]

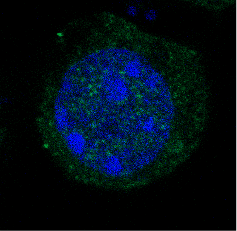

Supplement: Supplementary file 13 — Source data Fig. 8 [file 44319_2024_358_MOESM13_ESM.zip › Source data Figure 8/Figure 8B/inset 6h.tif]

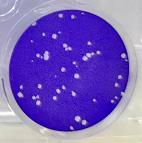

Supplement: Supplementary file 13 — Source data Fig. 8 [file 44319_2024_358_MOESM13_ESM.zip › Source data Figure 8/Figure 8G/HDAC6+HSV-1.jpg]

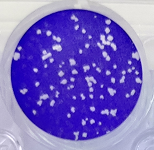

Supplement: Supplementary file 13 — Source data Fig. 8 [file 44319_2024_358_MOESM13_ESM.zip › Source data Figure 8/Figure 8G/HDAC6-NLS+HSV-1.png]

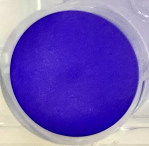

Supplement: Supplementary file 13 — Source data Fig. 8 [file 44319_2024_358_MOESM13_ESM.zip › Source data Figure 8/Figure 8G/vector.png]
