# Supplementary material for: HDAC6 deacetylates TRIM56 to negatively regulate cGAS-STING-mediated type I interferon responses
Source: EMBO Rep. 2025 Jan 2;26(3):720–47. doi: 10.1038/s44319-024-00358-5 (PMC11811133; doi:10.1038/s44319-024-00358-5)
Supplement: Supplementary file 8 — Source data Fig. 3 [file 44319_2024_358_MOESM8_ESM.zip › Source data Figure 3/Figure 3F.docx]

**Source Figure 3F**

HDAC6

**OB**


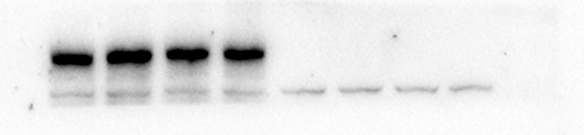

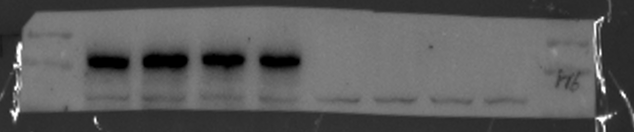


**170**

**130**




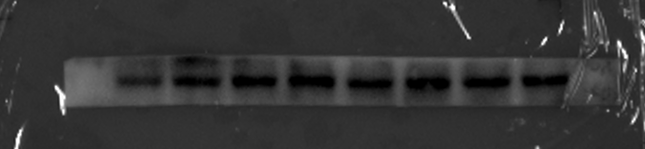


**55**

**70**

cGAS




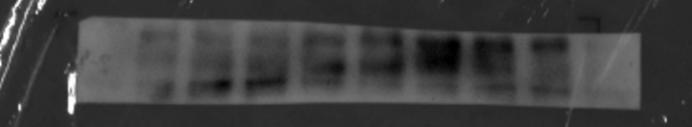


**35**

**40**

P-STING




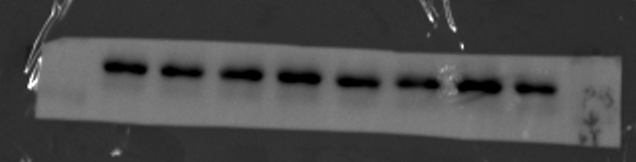


**35**

**40**

STING







**70**

**100**

P-TBK1




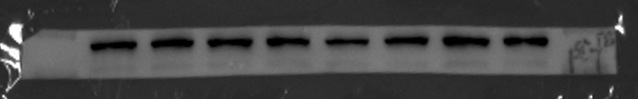


**100**

**70**

TBK1


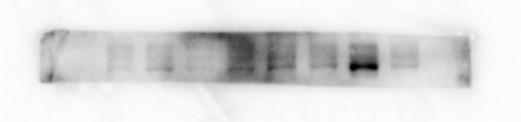

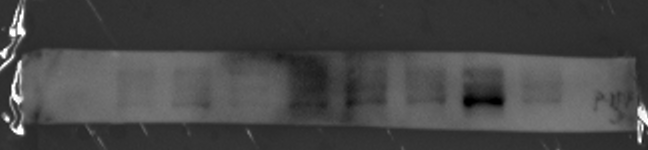


**40**

**55**

P-IRF3




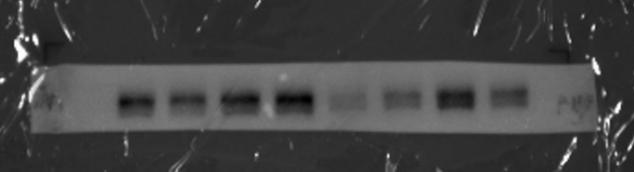


**40**

**55**

IRF3




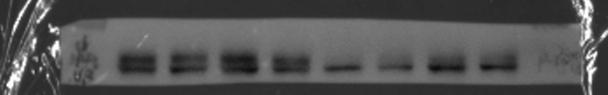


**40**

**55**

β-actin

**Source Figure 3F**

**CX**


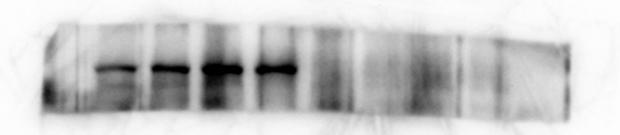

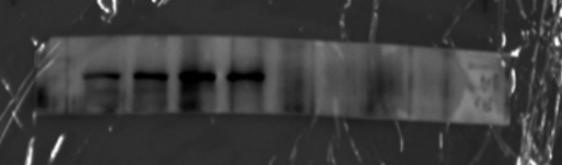


**130**

**170**

HDAC6


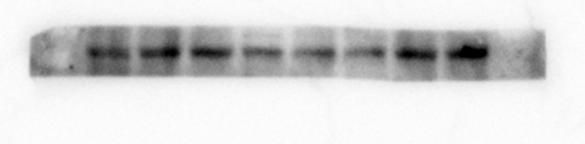




**55**

**70**

P-STING

cGAS


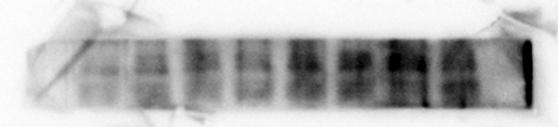

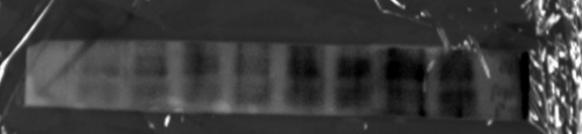


**40**

**35**


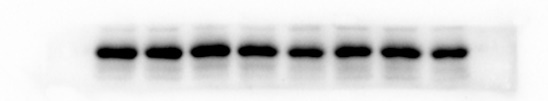

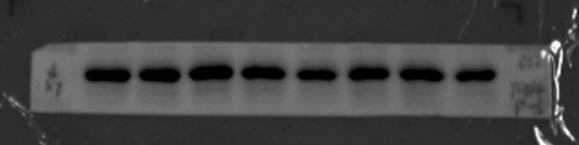


**40**

**35**

STING







**100**

**70**

P-TBK1


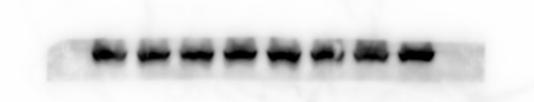

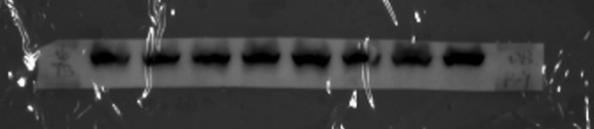


**70**

**100**

TBK1

**55**

P-IRF3


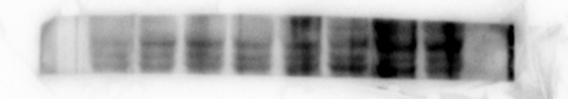

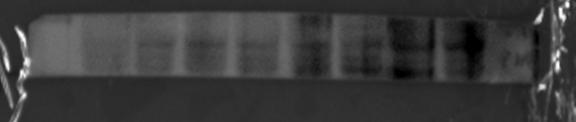


**40**


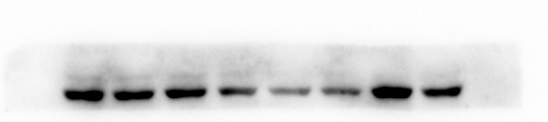

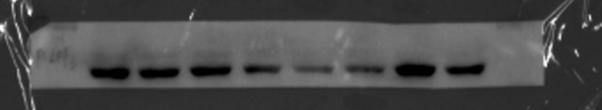


**40**

**55**

IRF3




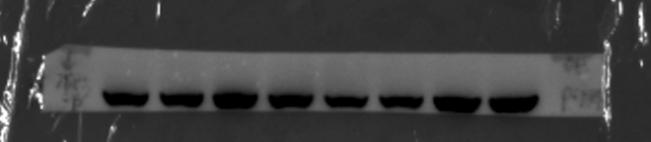


**55**

**40**

β-actin

**Source Figure 3F**

**P/M/C**

HDAC6


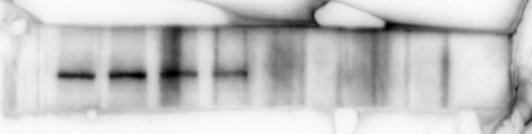

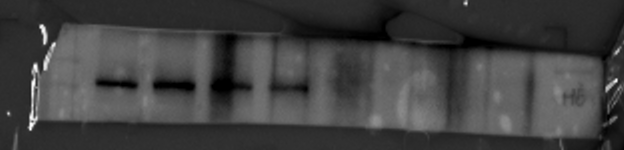


**130**

**170**


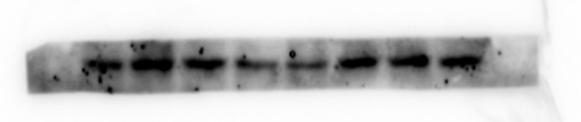




**70**

**55**

cGAS


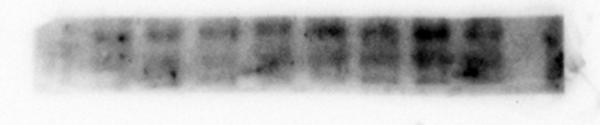

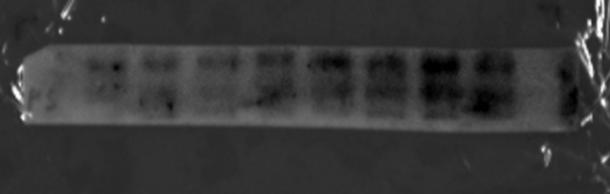


**40**

**35**

P-STING


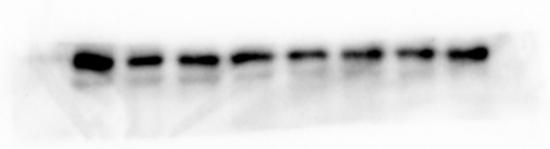

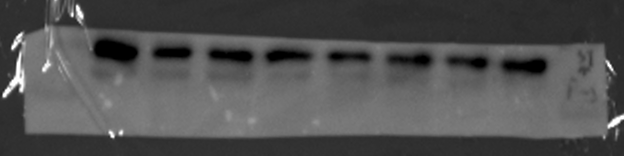


**35**

**40**

STING


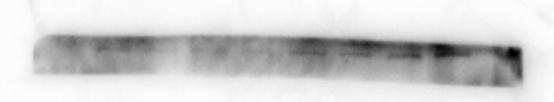




**70**

**100**

P-TBK1




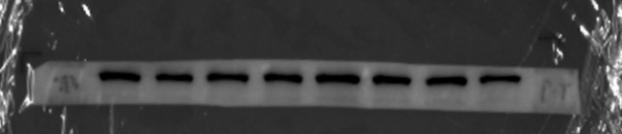


**100**

**70**

TBK1


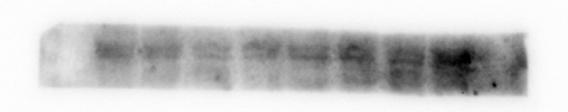




**40**

**55**

IRF3

P-IRF3


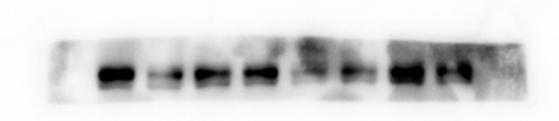

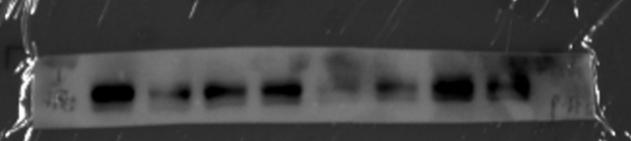


**55**

**40**




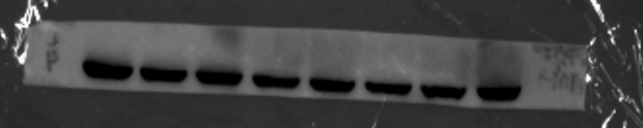


**40**

**55**

β-actin
