# Supplementary material for: HDAC6 deacetylates TRIM56 to negatively regulate cGAS-STING-mediated type I interferon responses
Source: EMBO Rep. 2025 Jan 2;26(3):720–47. doi: 10.1038/s44319-024-00358-5 (PMC11811133; doi:10.1038/s44319-024-00358-5)
Supplement: Supplementary file 9 — Source data Fig. 4 [file 44319_2024_358_MOESM9_ESM.zip › Source data Figure 4/Figure 4H-M.docx]

**Source Figure 4H**

IB: HDAC6


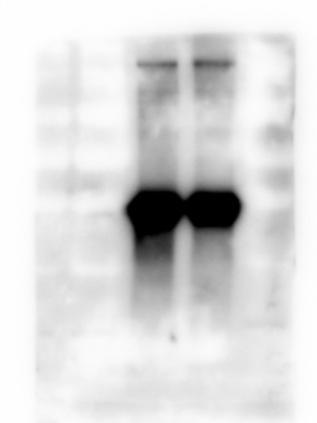

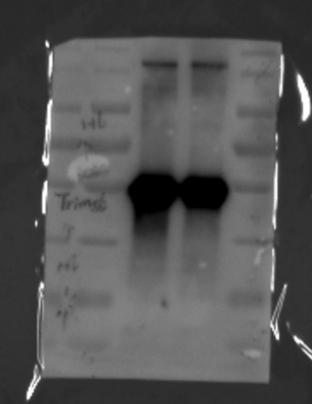


**25**

**35**

**40**

**55**

**70**

**100**

**130**

**170**

IB: TRIM56


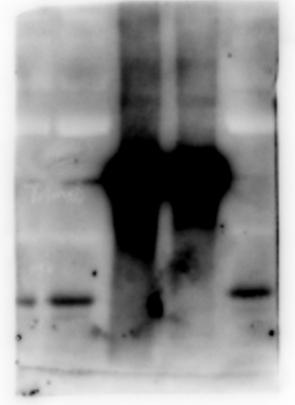

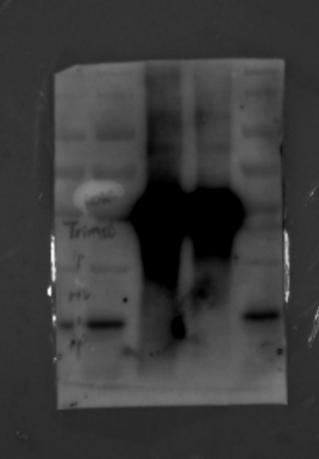


**170**

**130**

**100**

**55**

**25**

**35**

**40**

**70**


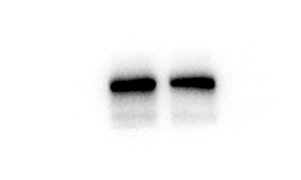

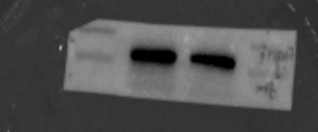


**130**

**170**

Input: HDAC6

Input: TRIM56


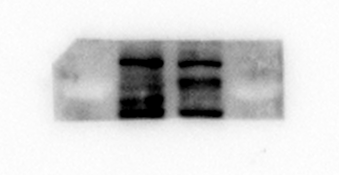

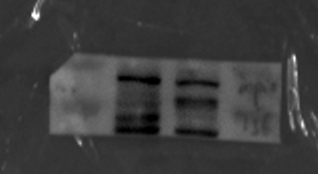


**100**

**70**


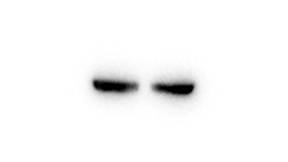

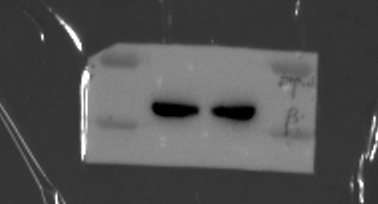


**40**

**55**

Input: actin

**Source Figure 4I**


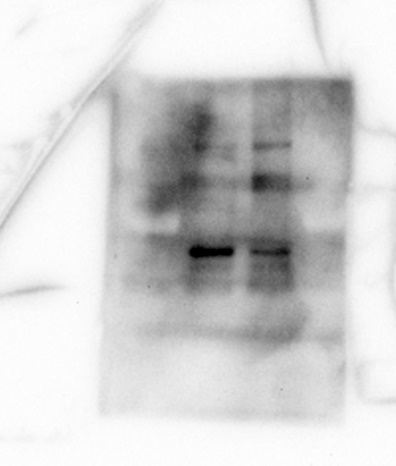

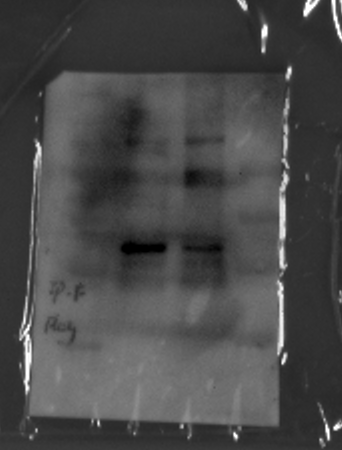


**40**

**55**

**70**

**130**

**100**

**170**

IB: FLAG




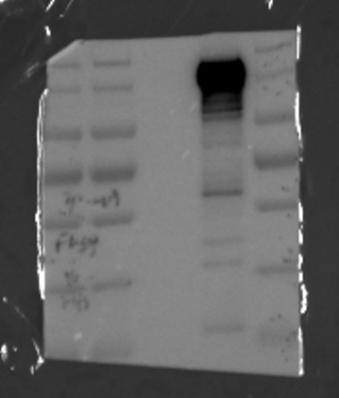


**35**

**100**

**130**

**40**

**55**

**70**

**170**

IB: HA

Input: FLAG


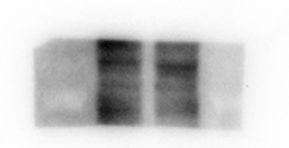

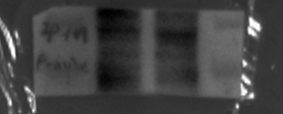


**70**

**100**




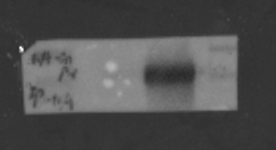


**130**

**170**

Input: HA

Input: β-actin


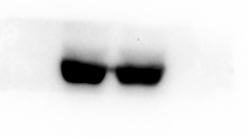

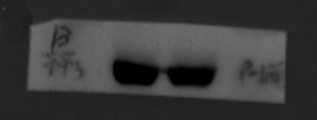


**40**

**55**

**Source Figure 4J**


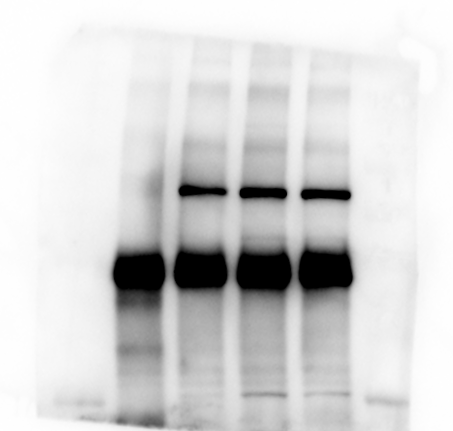

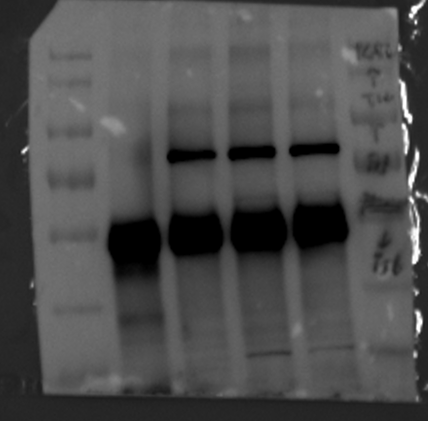


**35**

**40**

**55**

**70**

**100**

**170**

**130**

IB: TRIM56


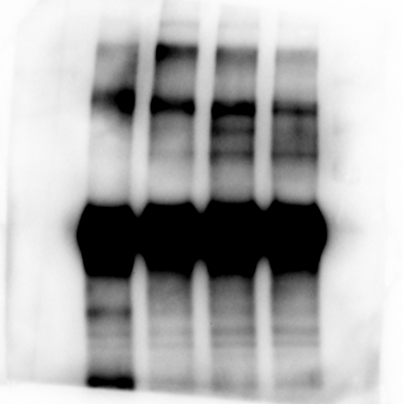

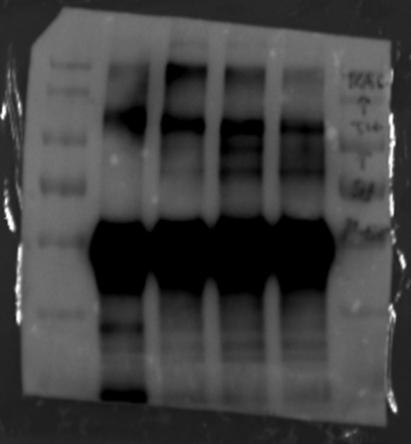


**130**

**100**

**70**

**35**

**40**

**55**

**170**

IB: Acetyllysine


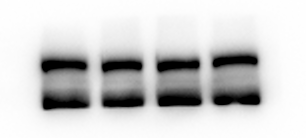

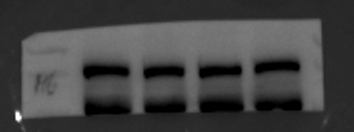


**130**

**170**

Input: HDAC6


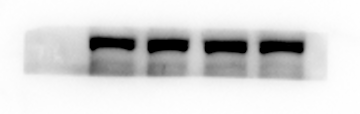

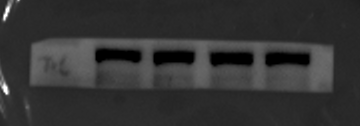


**100**

**70**

Input: TRIM56

Input: β-actin


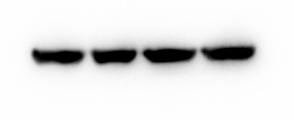

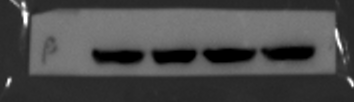


**40**

**55**

**Source Figure 4K**

IB: TRIM56


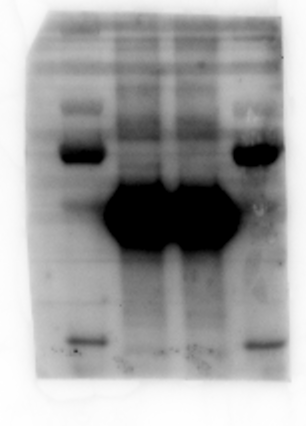

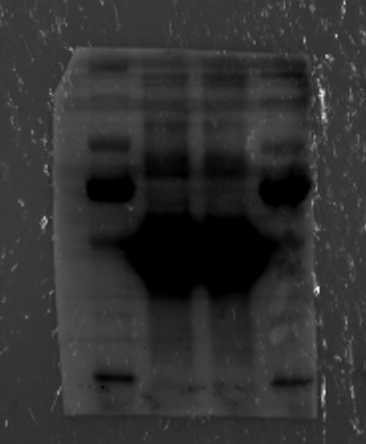


**35**

**40**

**55**

**70**

**130**

**100**

**170**

IB: Acetyllysine


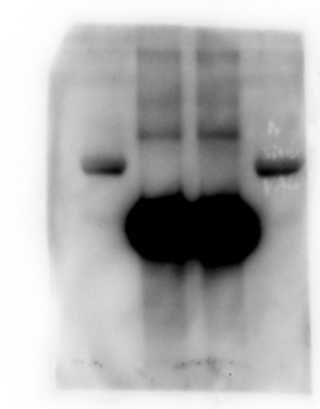

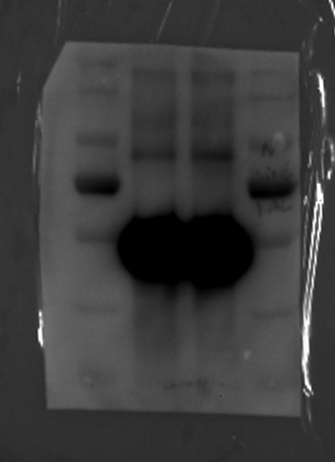


**35**

**40**

**55**

**170**

**70**

**130**

**100**

Input: HDAC6


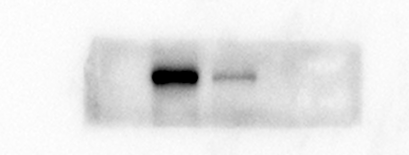

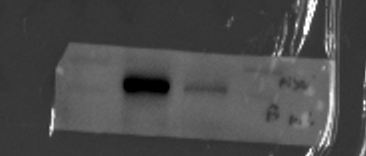


**170**

**130**


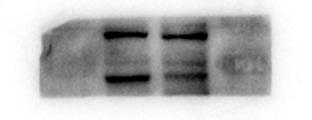

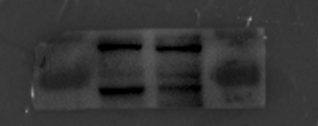


**70**

**100**

Input: TRIM56


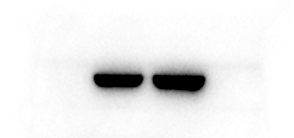

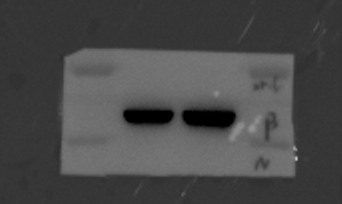


**40**

**55**

Input: β-actin

**Source Figure 4L**

IB: Acetyllysine


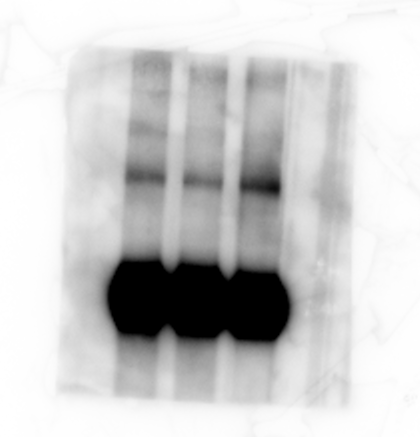

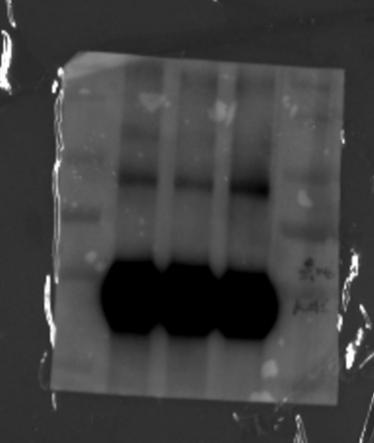


**40**

**55**

**70**

**100**

**130**

**170**


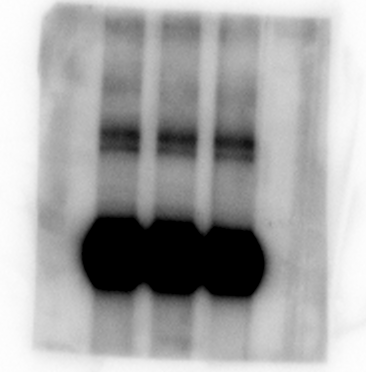

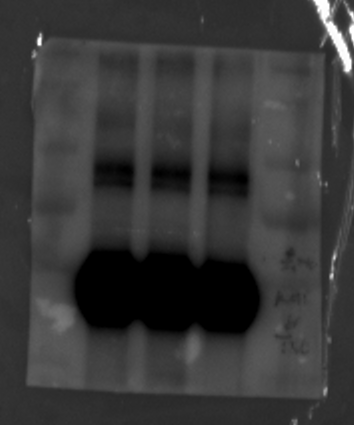


**40**

**55**

**130**

**170**

**70**

**100**

IB: TRIM56


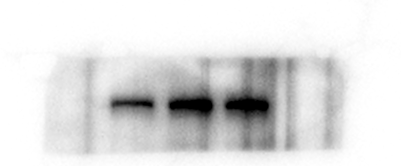

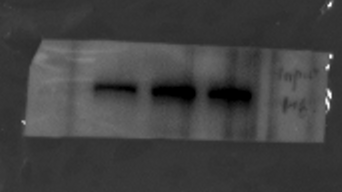


**170**

**130**

Input: HDAC6


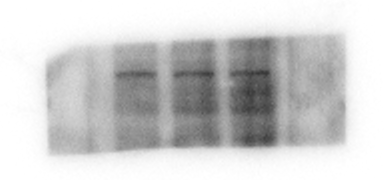

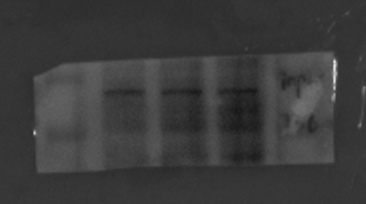


**100**

**70**

Input: TRIM56


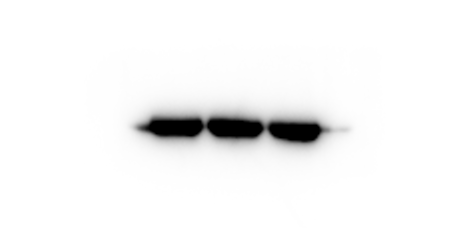

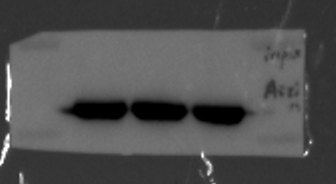


**55**

**40**

Input: β-actin

**Source Figure 4M**

IB: Acetyllysine


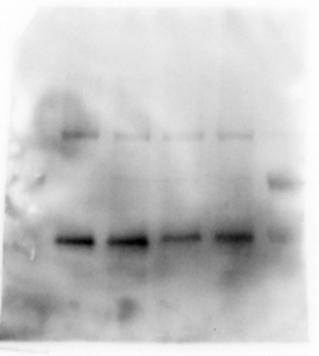

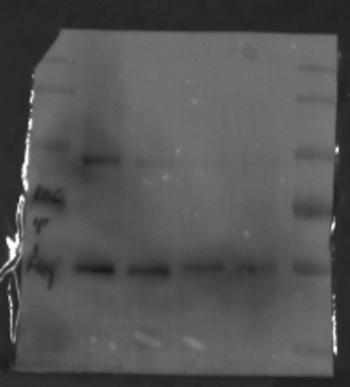


IB: Flag

**40**

**55**

**70**

**100**

**130**

**170**


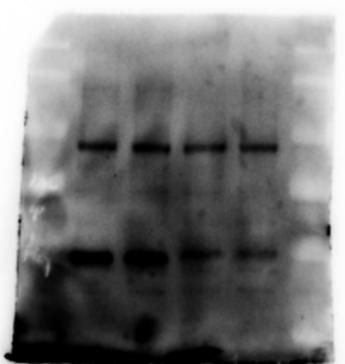

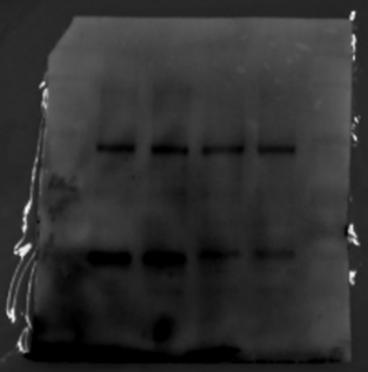


**40**

**170**

**55**

**70**

**100**

**130**

Input: Flag


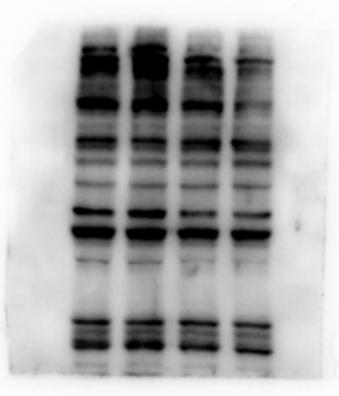

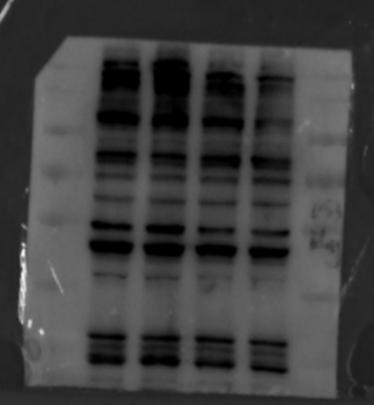


**35**

**40**

**55**

**70**

**130**

**100**

**170**




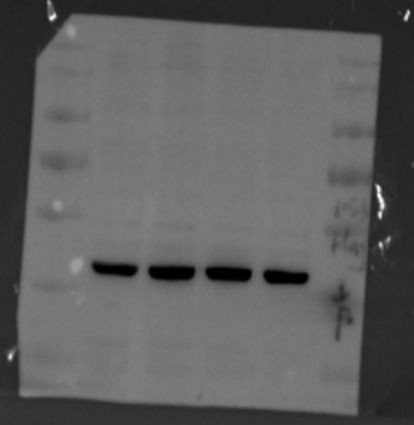


Input: β-actin

**35**

**40**

**55**

**70**

**100**

**130**

**170**
