# Supplementary material for: HDAC6 deacetylates TRIM56 to negatively regulate cGAS-STING-mediated type I interferon responses
Source: EMBO Rep. 2025 Jan 2;26(3):720–47. doi: 10.1038/s44319-024-00358-5 (PMC11811133; doi:10.1038/s44319-024-00358-5)
Supplement: Supplementary file 10 — Source data Fig. 5 [file 44319_2024_358_MOESM10_ESM.zip › Source data Figure 5/Figure 5D,G,H.docx]

**Source Figure 5D**


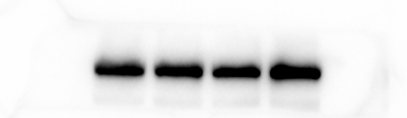

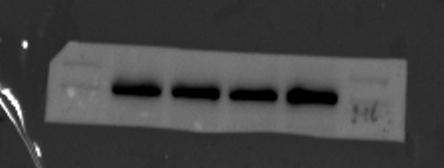


**130**

**170**

HDAC6

ICP0


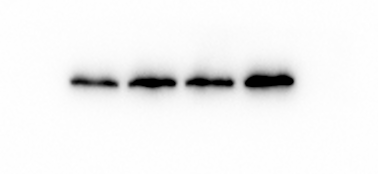

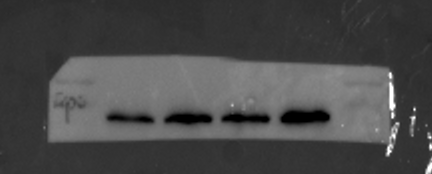


**100**

**130**

**170**

cGAS


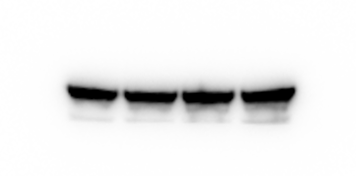

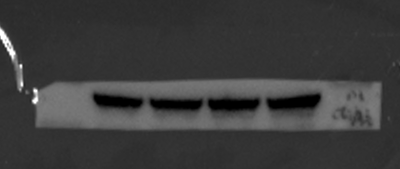


**55**

**70**

STING


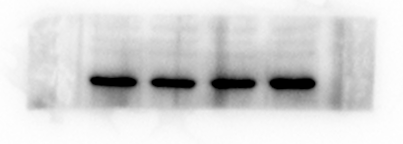

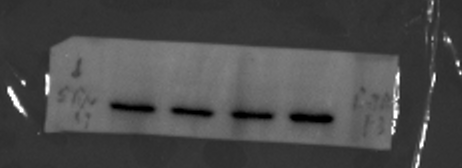


**55**

**40**

**35**


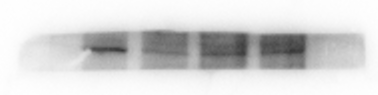

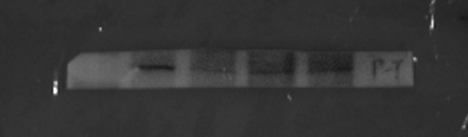


**70**

**100**

P-TBK1


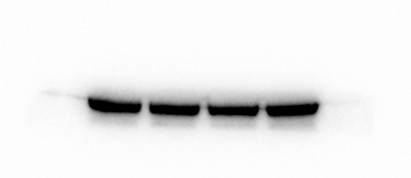

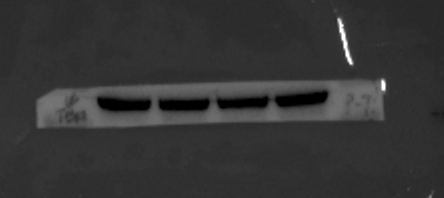


**70**

**100**

TBK1


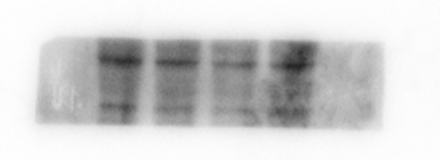

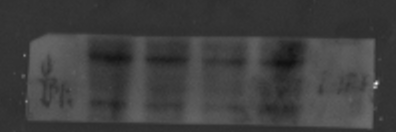


**35**

**40**

**55**

IRF3

P-IRF3


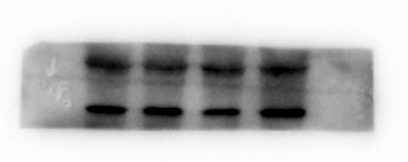

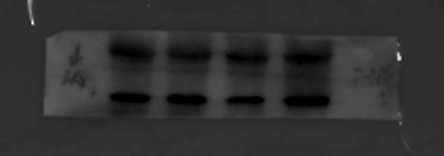


**35**

**40**

**55**


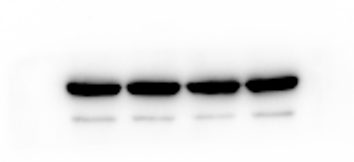

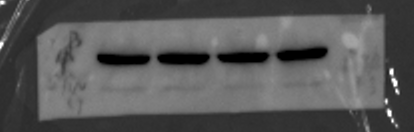


**35**

**40**

**55**

β-actin


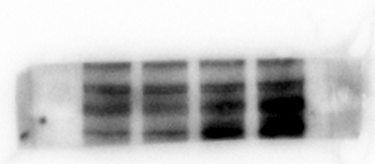

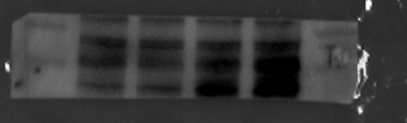


**70**

**100**

TRIM56

**Source Figure 5G**


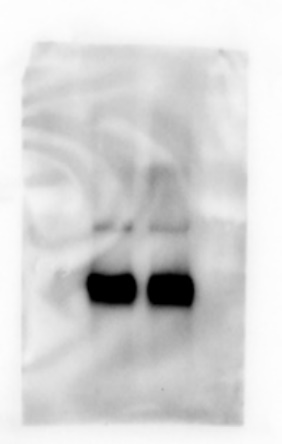

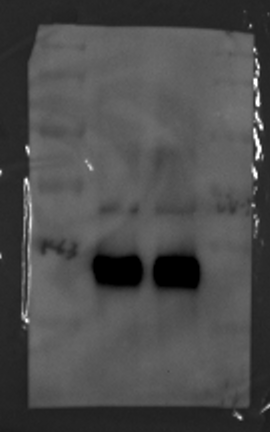


IB:VU-1

**40**

**55**

**70**

**170**

**130**

**100**

**35**

IB:cGAS


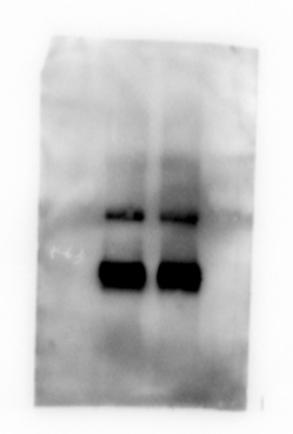

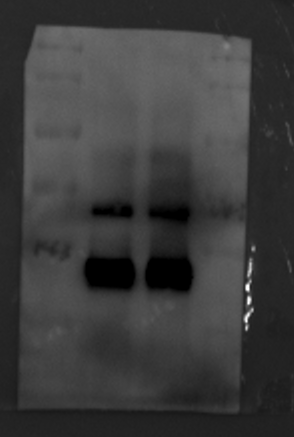


**130**

**100**

**70**

**55**

**40**

**170**

**35**

input:cGAS


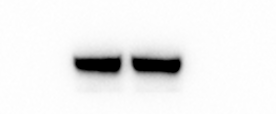

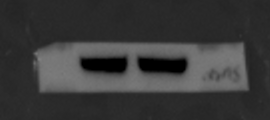


**70**

**55**

input:β-actin


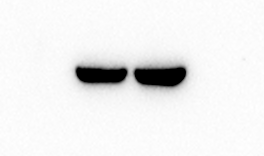

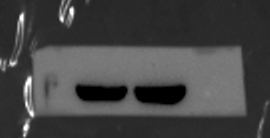


**40**

**55**

**Source Figure 5H**


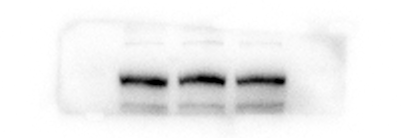

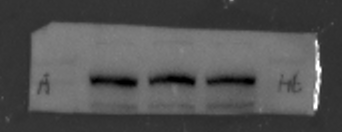


**130**

**170**

P-TBK1

HDAC6


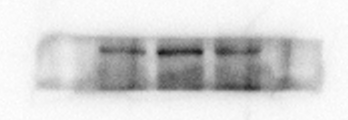

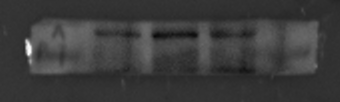


**70**

**100**


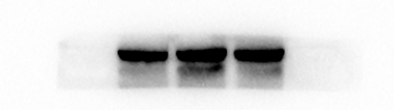

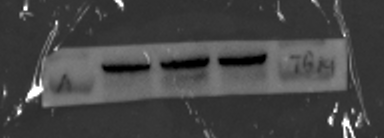


**100**

**70**

TBK1


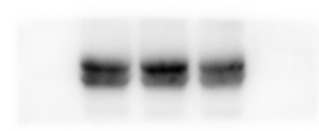

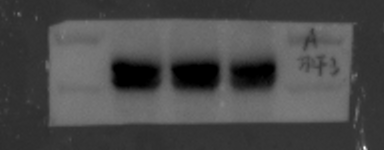


**55**

**40**

P-IRF3


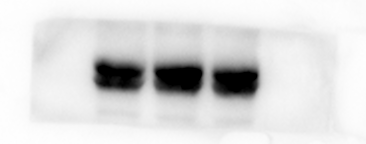

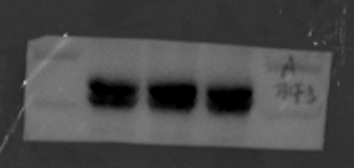


**55**

**40**

IRF3


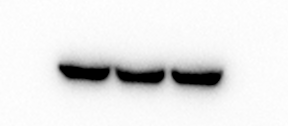

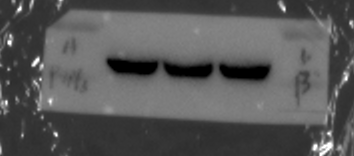


**55**

**40**

β-actin
