# Supplementary material for: HDAC6 deacetylates TRIM56 to negatively regulate cGAS-STING-mediated type I interferon responses
Source: EMBO Rep. 2025 Jan 2;26(3):720–47. doi: 10.1038/s44319-024-00358-5 (PMC11811133; doi:10.1038/s44319-024-00358-5)
Supplement: Supplementary file 11 — Source data Fig. 6 [file 44319_2024_358_MOESM11_ESM.zip › Source data Figure 6/Figure 6B,C,D,E,F,G.docx]

**Source Figure 6B**


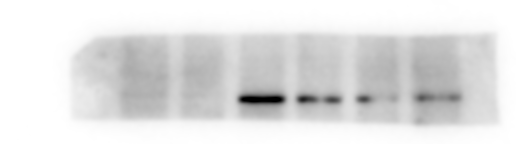

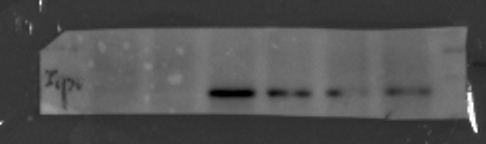


**130**

**100**

**170**

cell

ICP0


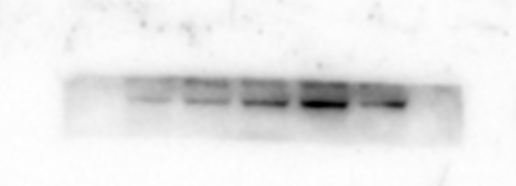

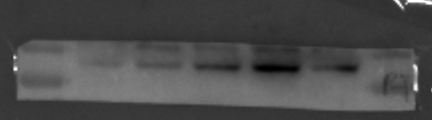


**70**

**100**

P-TBK1


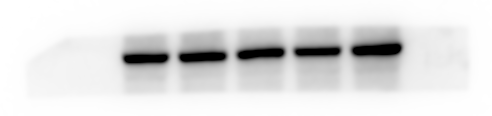

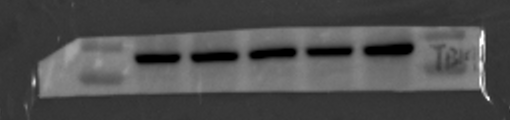


**70**

**100**

P-IRF3

TBK1


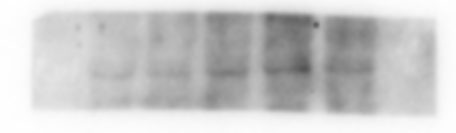

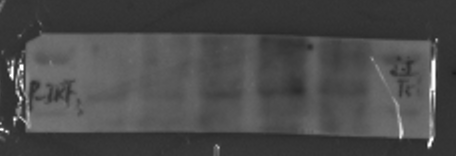


**40**

**55**

IRF3


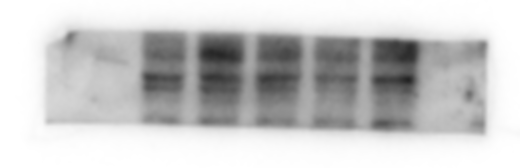

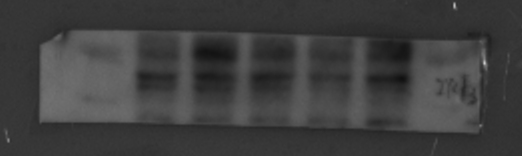


**40**

**55**

TRIM56


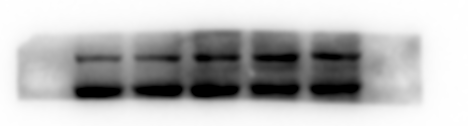

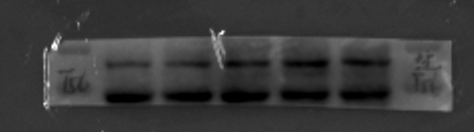


**70**

**100**

β-actin


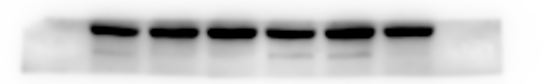

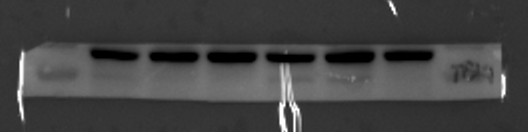


**55**

**40**

cell

**Source Figure 6C**


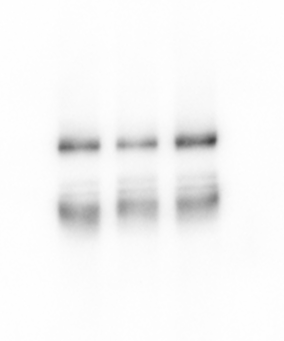

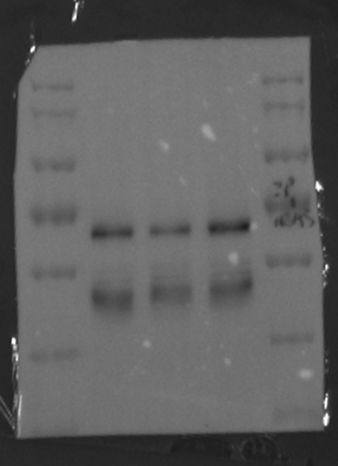


IB:VU-1

**35**

**40**

**55**

**70**

**100**

**130**

**170**




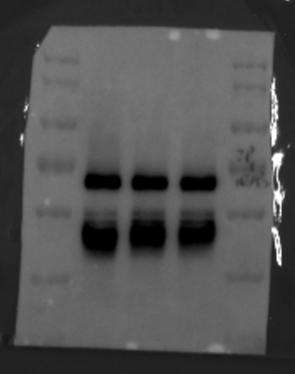


IB:cGAS

**35**

**40**

**55**

**70**

**100**

**170**

**130**

IB:TRIM56


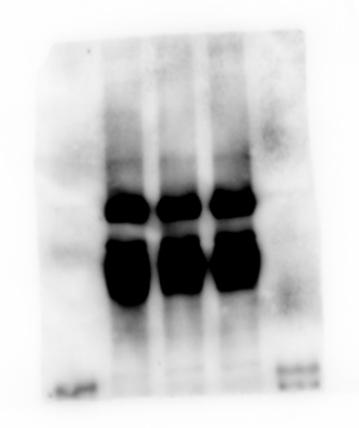

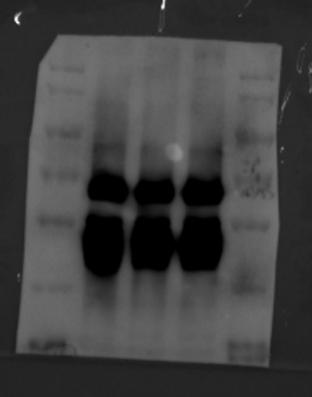


**35**

**170**

**40**

**55**

**70**

**100**

**130**

Input:HDAC6


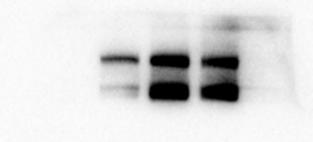

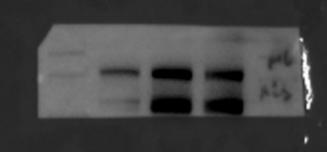


**100**

**170**

**130**

Input:TRIM56


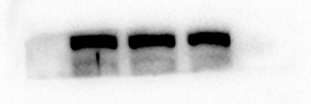




**100**

**70**

Input:cGAS


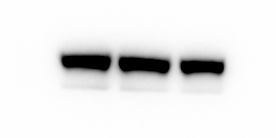

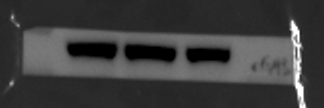


**55**

**70**


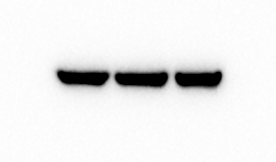

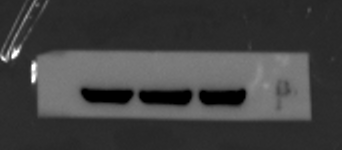


Input:β-actin

**55**

**40**

**Source Figure 6D**

IB:TRIM56


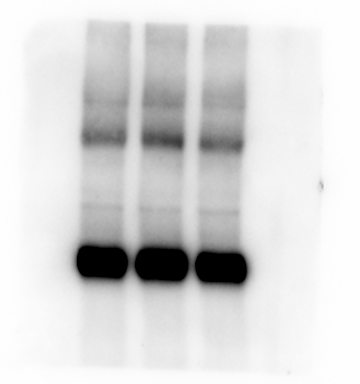

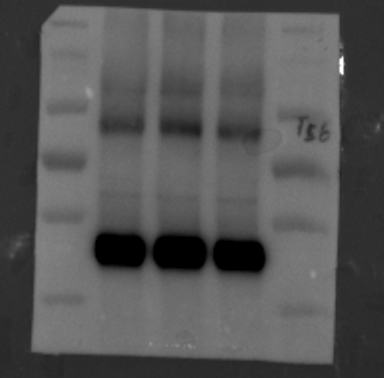


**130**

**170**

**55**

**40**

**100**

**70**


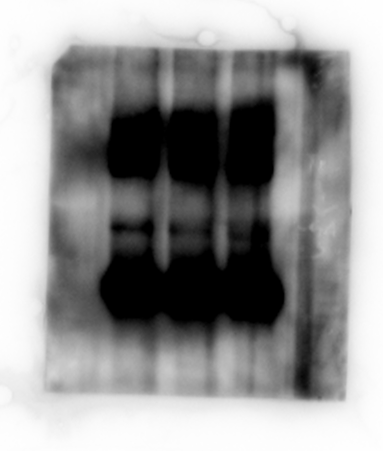

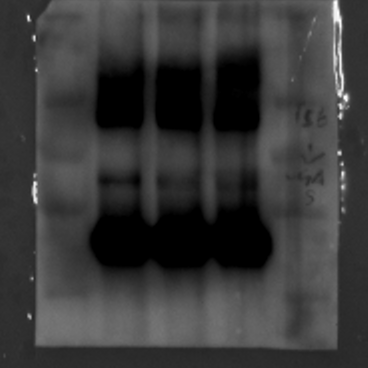


**40**

**55**

**70**

**130**

**170**

**100**

IB:cGAS

Input:cGAS


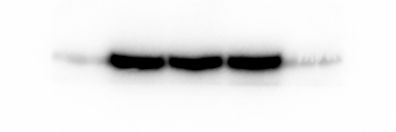

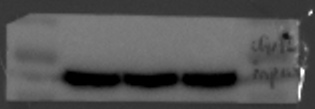


**55**

**70**

**100**


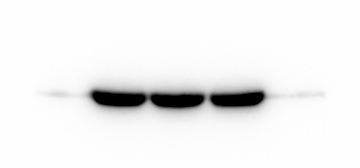

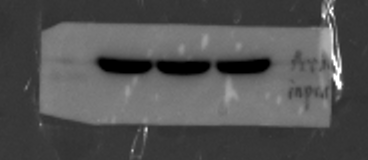


Input:β-actin

**35**

**40**

**55**

**Source Figure 6E**

IB: VU-1


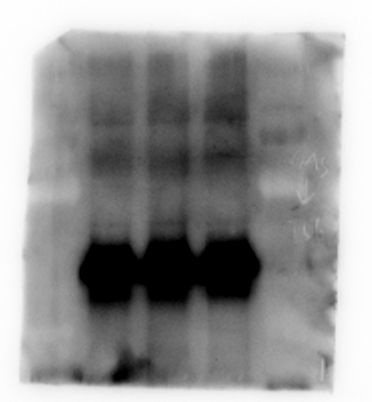

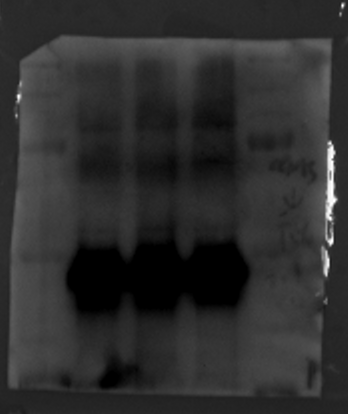


**70**

**55**


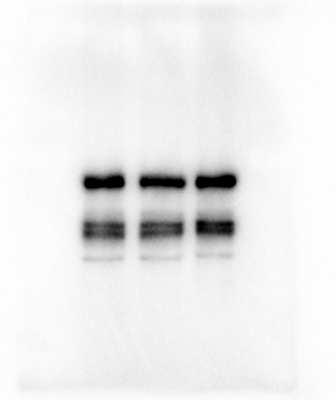

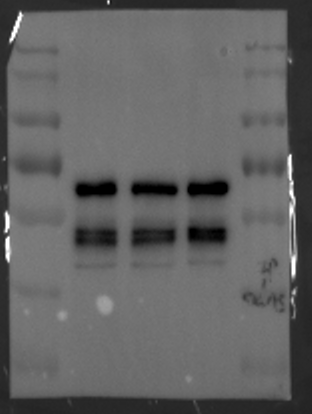


**35**

**40**

**100**

**130**

**170**

**70**

**55**

IB:cGAS


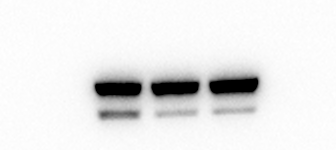

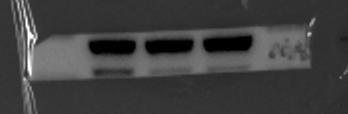


**55**

**70**

Input:cGAS


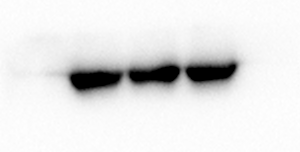

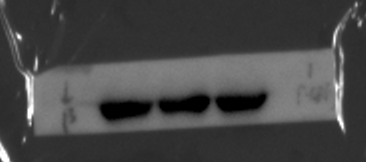


**40**

**55**

Input:β-actin

**Source Figure 6F**


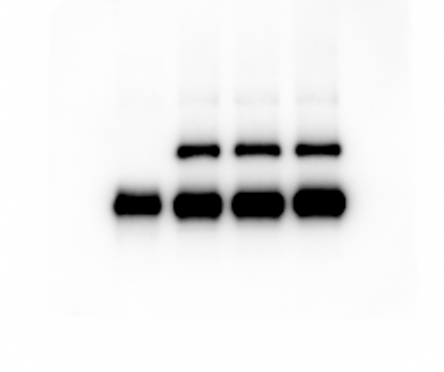

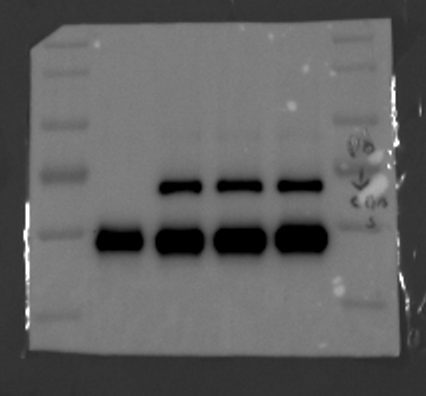


**40**

**55**

**70**

**100**

**130**

**170**

IB:cGAS


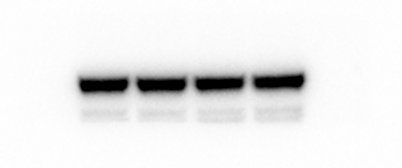

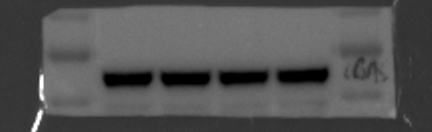


**55**

**70**

**100**

Input:cGAS


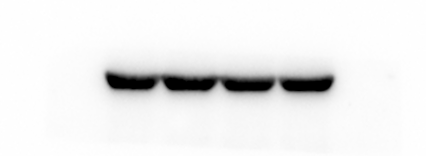

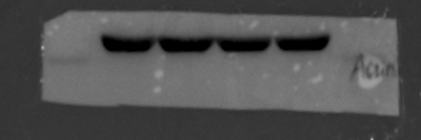


**40**

**55**

Input:β-actin

**Source Figure 6G**


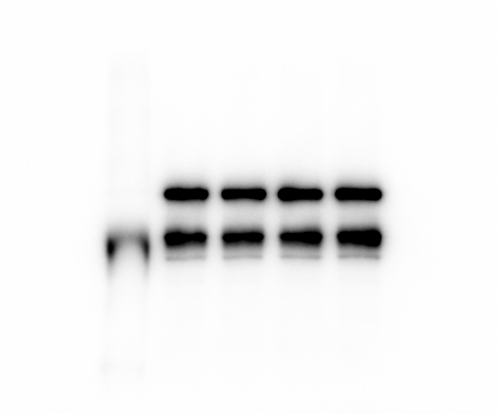

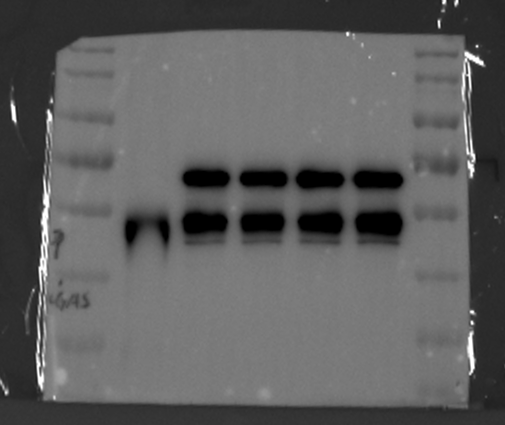


**25**

**35**

**40**

**55**

**70**

**100**

**130**

**170**

IB:cGAS


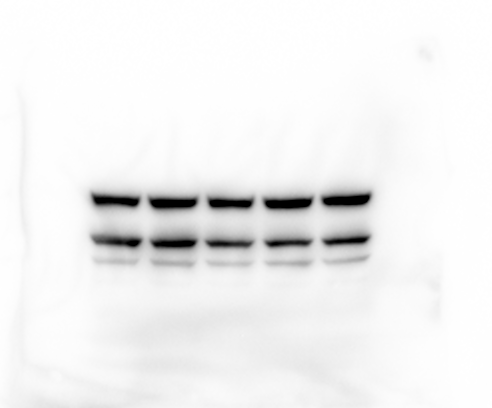

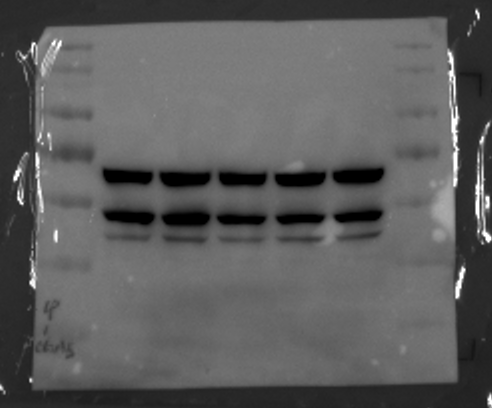


**130**

**35**

**40**

**55**

**70**

**170**

**100**

Input:cGAS

Input:β-actin


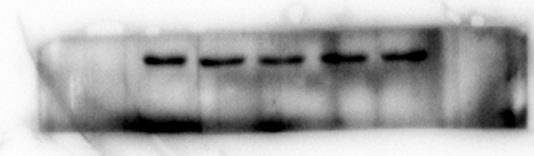

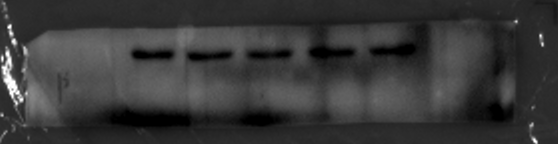


**40**

**35**

**55**
