# Supplementary material for: HDAC6 deacetylates TRIM56 to negatively regulate cGAS-STING-mediated type I interferon responses
Source: EMBO Rep. 2025 Jan 2;26(3):720–47. doi: 10.1038/s44319-024-00358-5 (PMC11811133; doi:10.1038/s44319-024-00358-5)
Supplement: Supplementary file 12 — Source data Fig. 7 [file 44319_2024_358_MOESM12_ESM.zip › Source data Figure 7/Figure 7B,E.docx]

**Source Figure 7B**

TRIM56


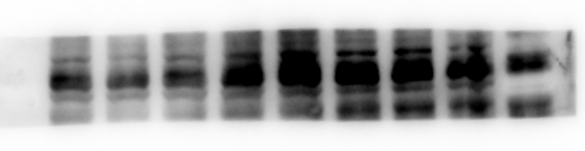


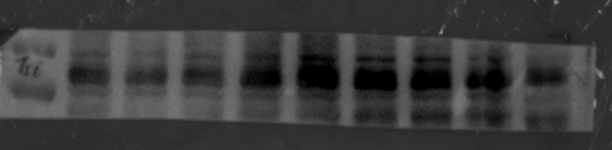


**70**

**100**

β-actin


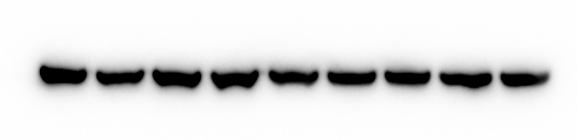


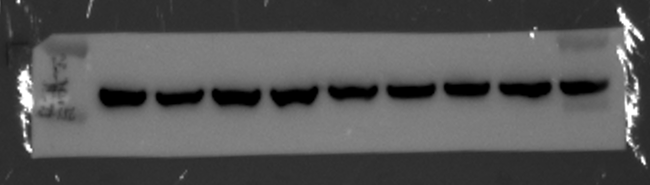


**40**

**55**

**Source Figure 7E OB**

**
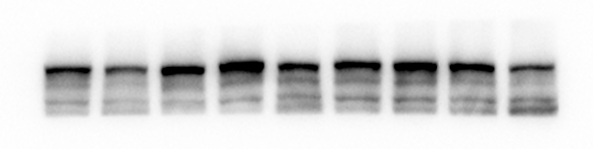

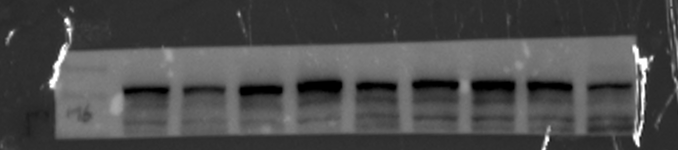
**

**100**

**130**

**170**

P-STING

HDAC6


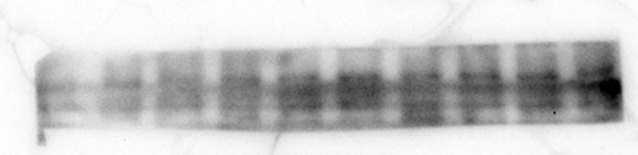

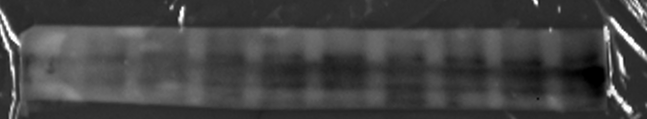


**40**

**35**


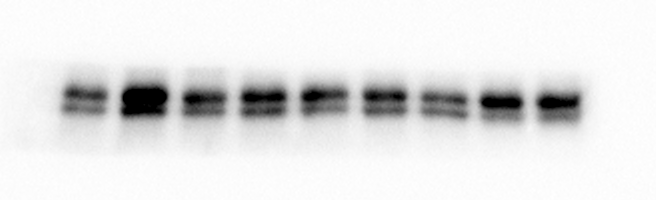

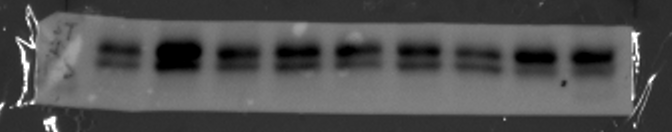


**40**

**35**

P-TBK1

STING


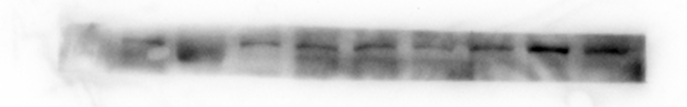

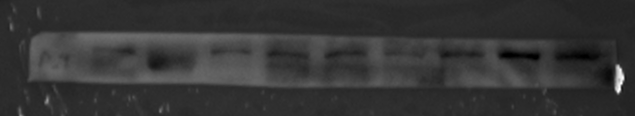


**70**

**100**


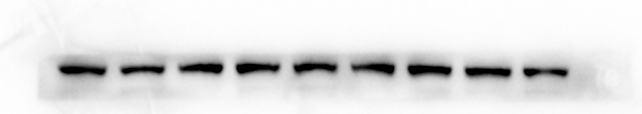

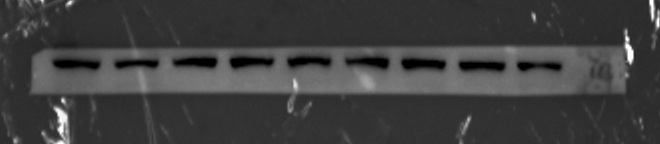


**100**

**70**

TBK1

P-IRF3


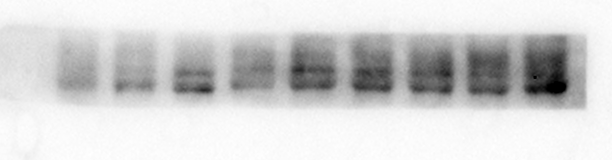

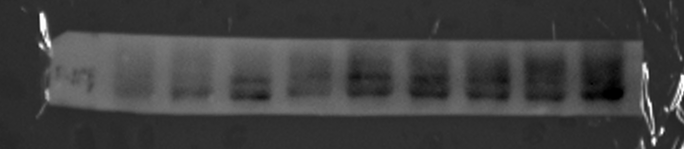


**40**

**55**

IRF3


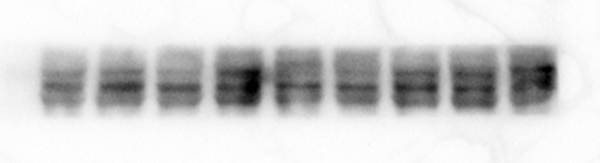

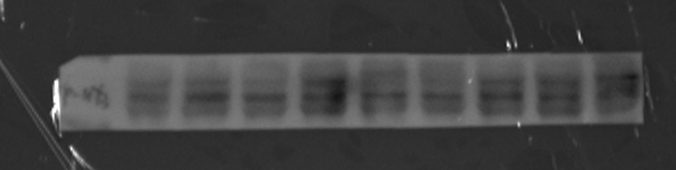


**55**

**40**

β-actin


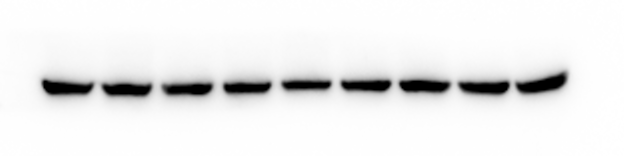

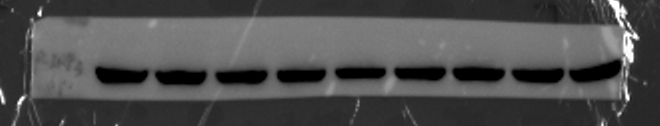


**55**

**40**

**Source Figure 7E CX**


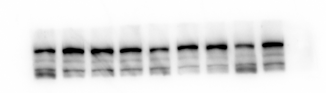

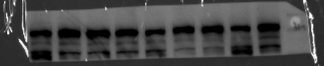


**100**

**130**

**170**

HDAC6


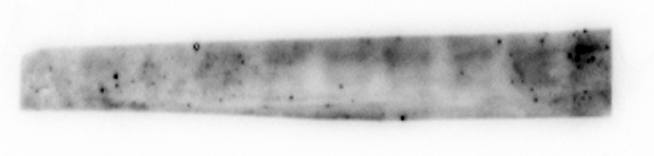

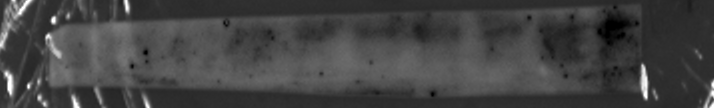


**35**

**40**

P-STING


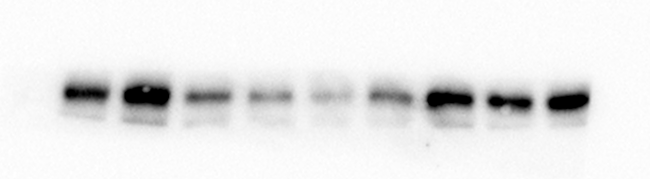

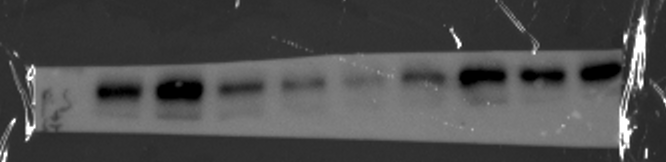


**35**

**40**

P-TBK1

STING


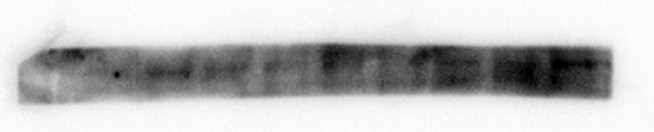

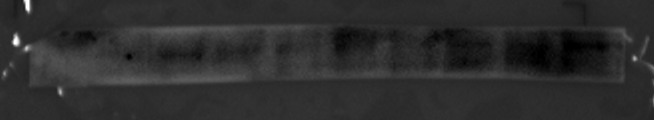


**70**

**100**


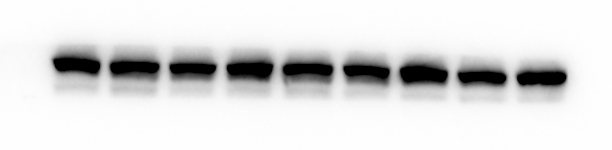

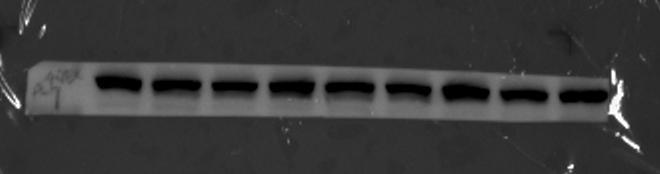


**100**

**70**

TBK1


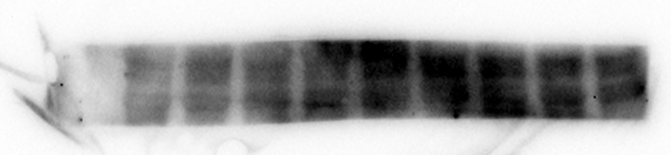

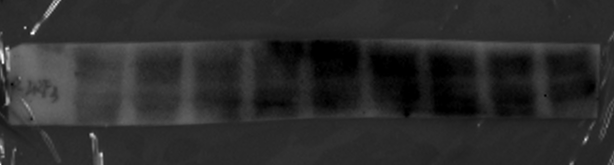


**40**

**55**

P-IRF3


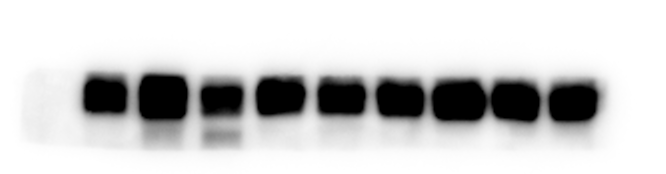

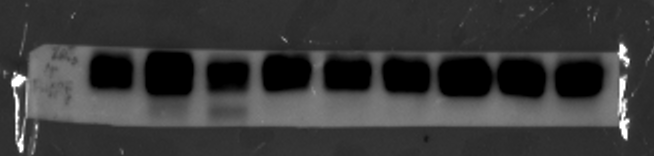


**55**

**40**

IRF3


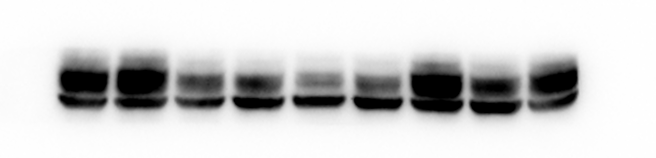

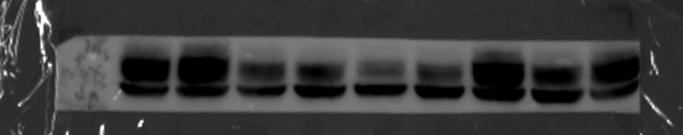


**40**

**55**

β-actin
