# Supplementary material for: HDAC6 deacetylates TRIM56 to negatively regulate cGAS-STING-mediated type I interferon responses
Source: EMBO Rep. 2025 Jan 2;26(3):720–47. doi: 10.1038/s44319-024-00358-5 (PMC11811133; doi:10.1038/s44319-024-00358-5)
Supplement: Supplementary file 13 — Source data Fig. 8 [file 44319_2024_358_MOESM13_ESM.zip › Source data Figure 8/Figure 8C,D,E,H,I,J.docx]

**Source Figure 8C**


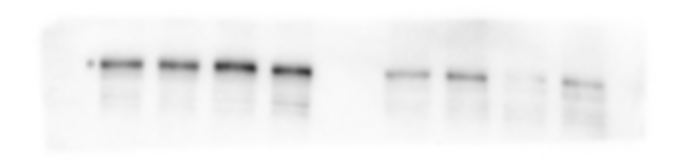


HDAC6


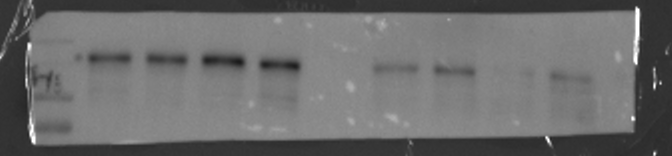


**130**

**100**

**170**

GAPDH


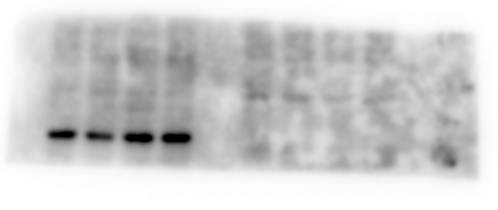


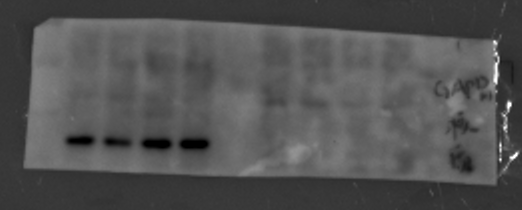


**35**

**40**

**55**

**70**

Histone3


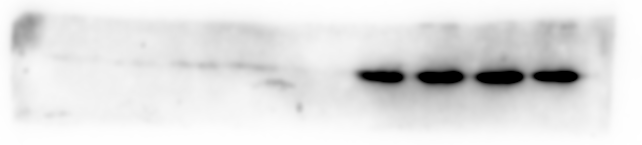


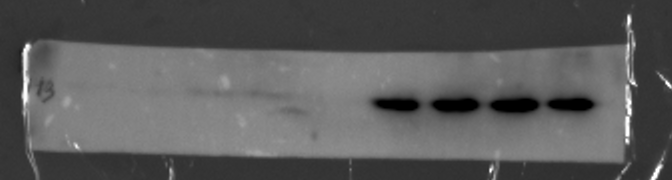


**25**

**Source Figure 8D**

IB: HDAC6


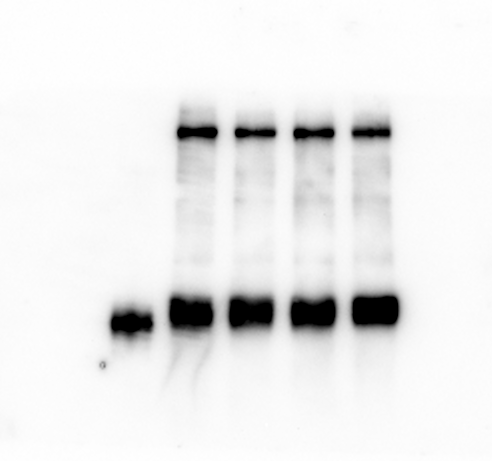

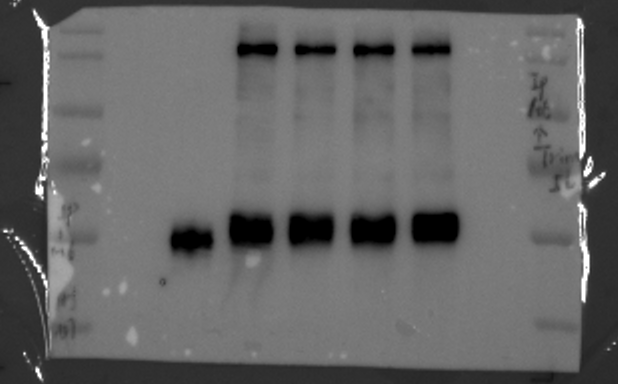


**40**

**55**

**70**

**170**

**130**

**100**


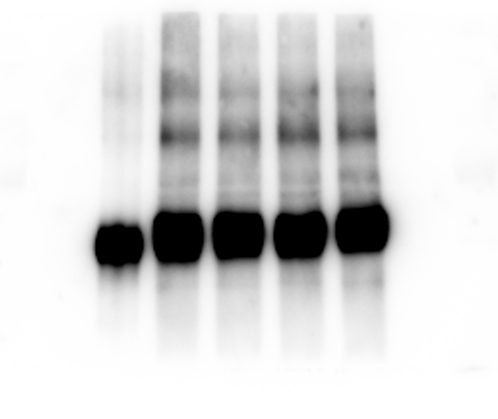

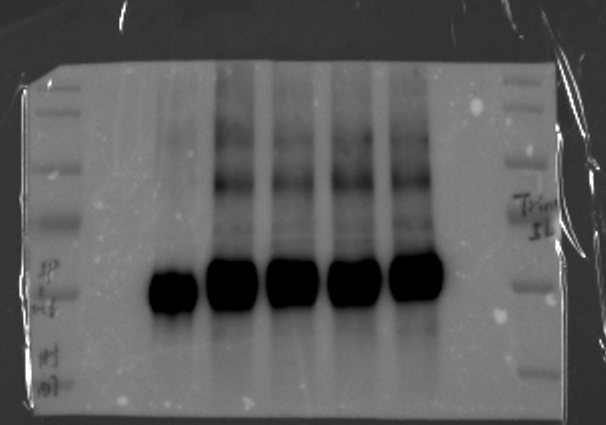


**100**

**40**

**55**

**70**

**170**

**130**

IB: TRIM56

Input: HDAC6


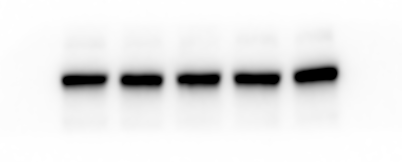

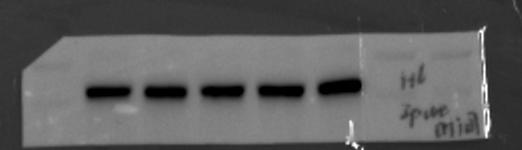


**100**

**130**

**170**


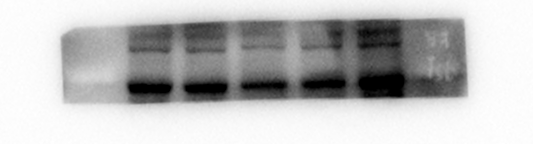

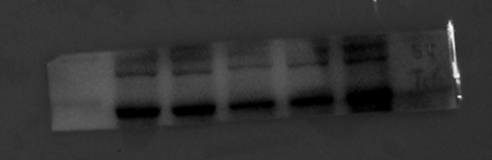


**70**

**100**

Input: TRIM56


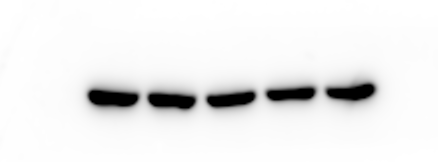

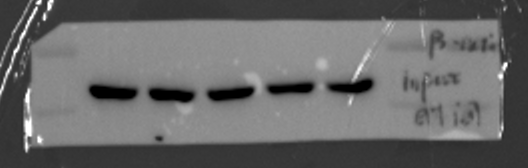


**55**

**40**

Input: β-actin

**Source Figure 8E**


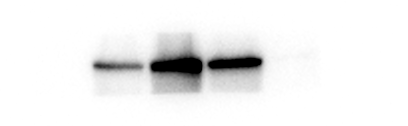

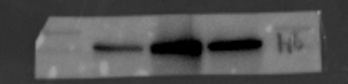


**130**

**100**

HDAC6

**170**


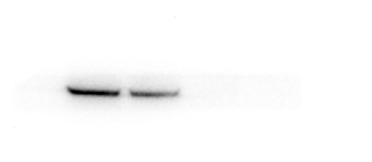

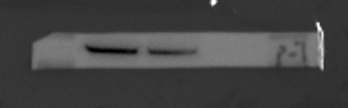


**70**

**100**

P-TBK1

TBK1


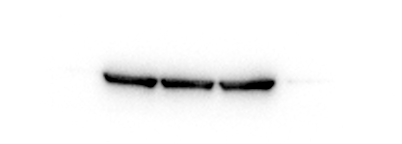

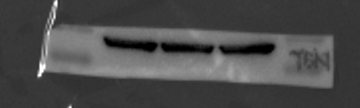


**100**

**70**


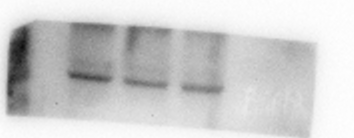

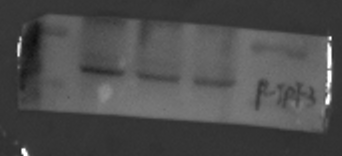


**40**

p-IRF3

**55**


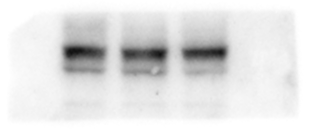

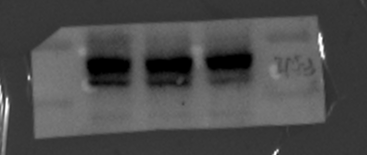


**55**

**40**

IRF3


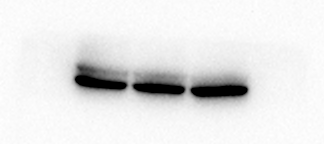

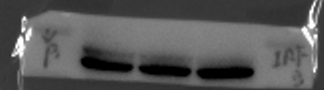


**55**

**40**

β-actin

**Source Figure 8F**

**
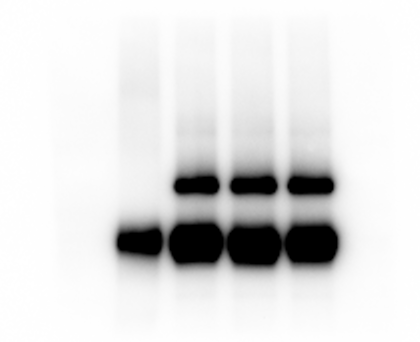

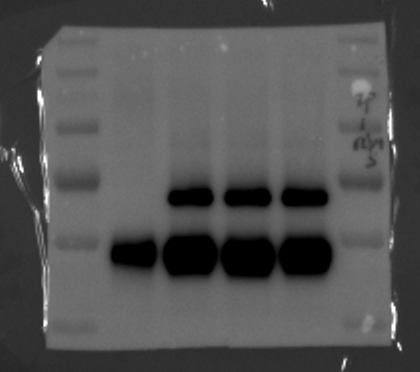
**

IgG

**40**

**55**

**70**

**100**

**130**

**170**

IB: cGAS

**
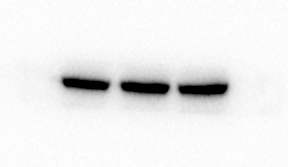

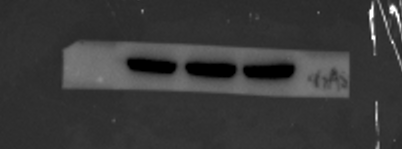
**

**70**

**55**

Input: cGAS

**
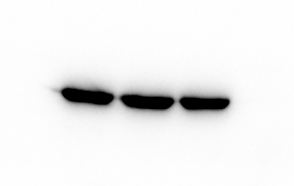

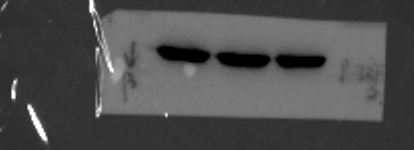
**

**40**

**55**

Input: β-actin

**Source Figure 8H**

IB: P-ser

**
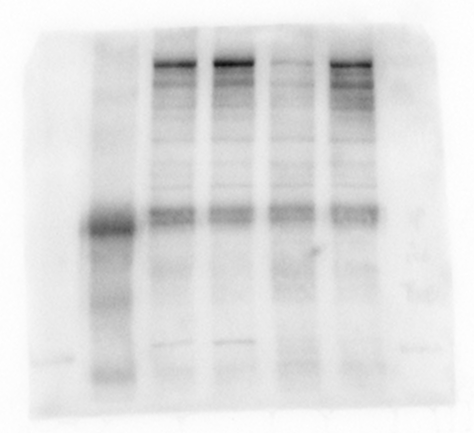

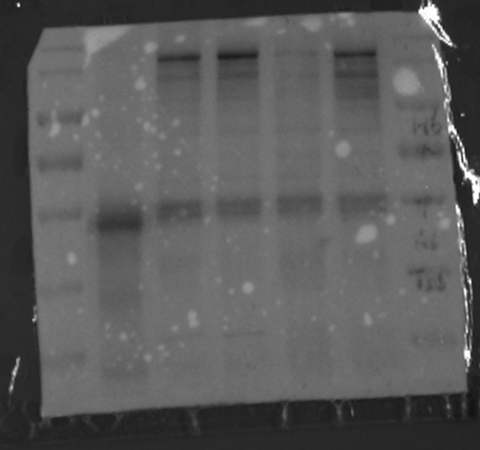
**

**130**

**170**

**
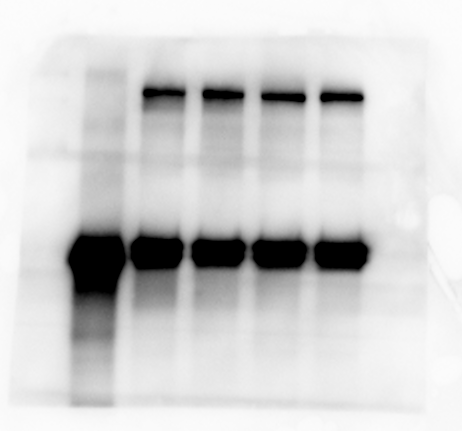

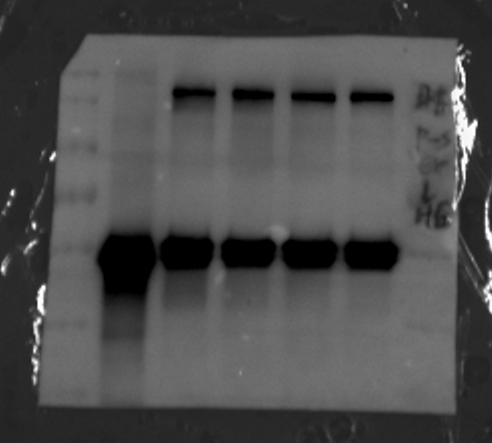
**

IB: HDAC6

**130**

**170**

Input: HDAC6

**
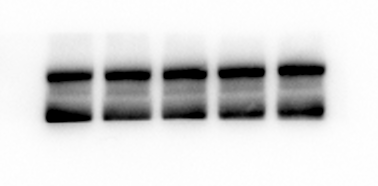

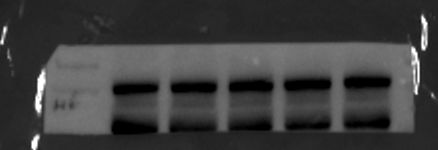
**

**130**

**170**

Input: β-actin

**
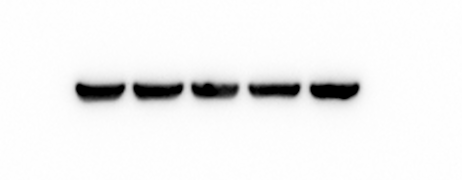

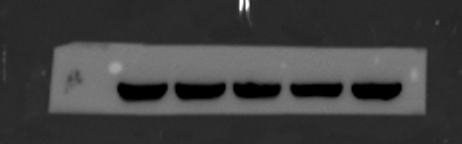
**

**55**

**40**

**Source Figure 8I**

IB: HDAC6

**
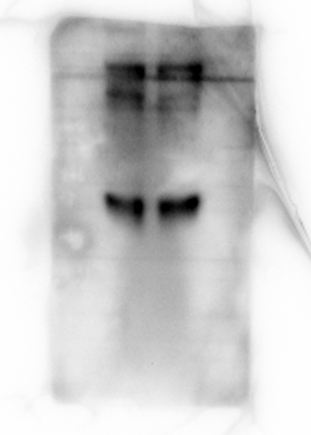

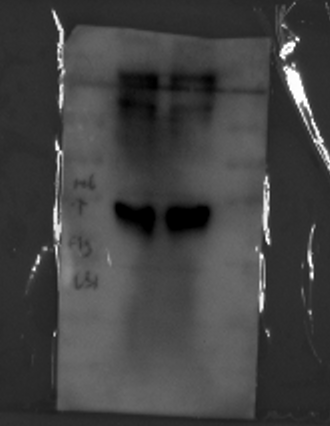
**

**40**

**55**

**70**

**100**

**130**

**170**

IB: FLAG-US3

**
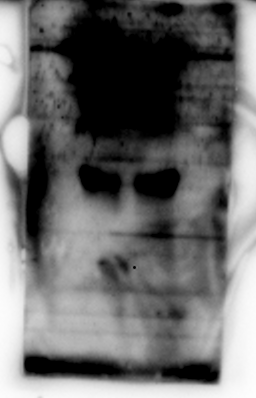

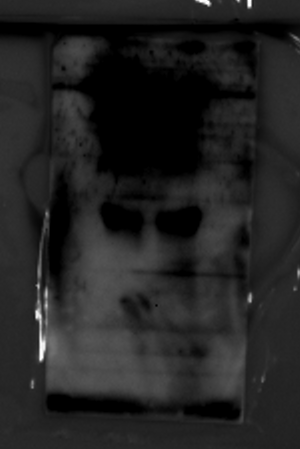
**

**70**

**40**

**55**

**100**

**130**

**170**

**
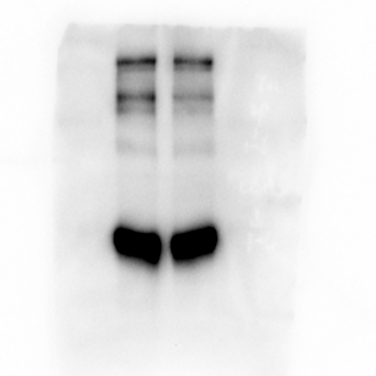

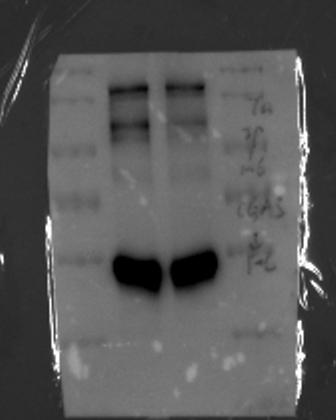
**

**40**

**55**

**70**

**100**

**130**

**170**

IB: HDAC6

**
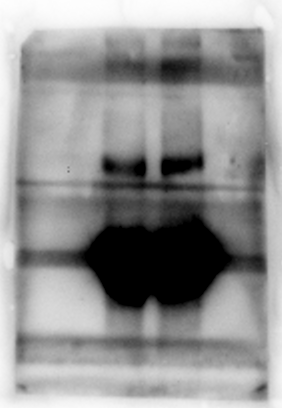

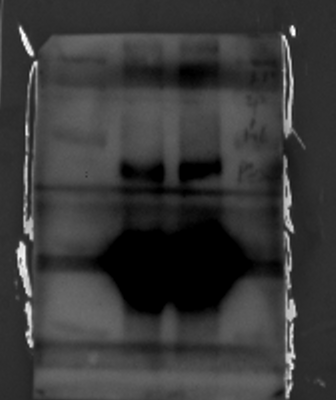
**

**55**

**100**

**130**

**170**

IB: phosphoserine

**
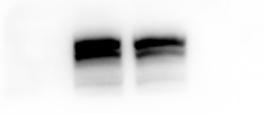

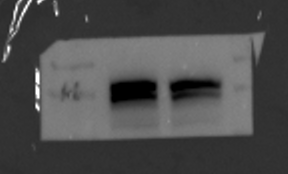
**

**100**

**170**

**130**

Input: HDAC6

Input: FLAG-US3

**
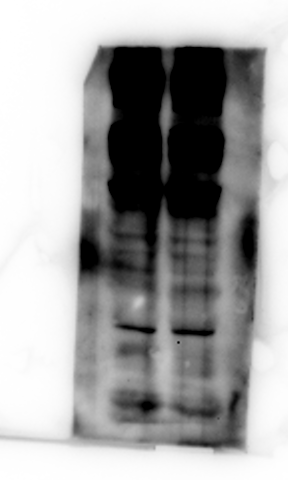

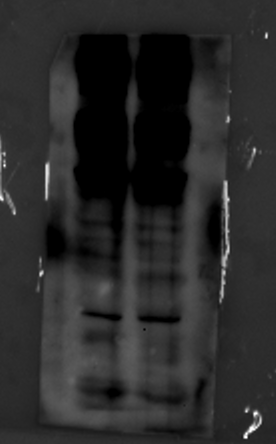
**

**100**

**70**

**55**

**35**

**40**

**130**

**170**

**
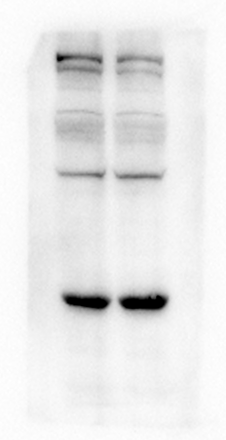

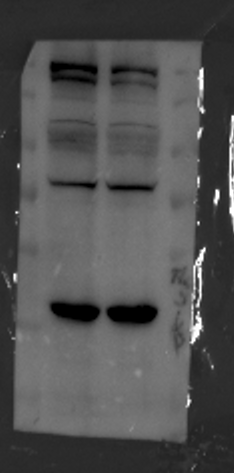
**

**35**

**55**

**40**

**70**

**100**

**130**

**170**

Input: β-actin

**Source Figure 8J**




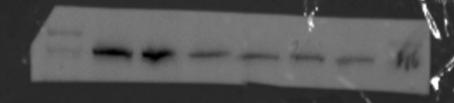


**100**

**130**

**170**

HDAC6

P-TBK1


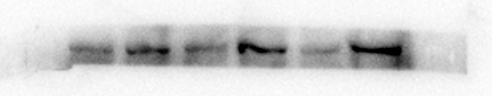

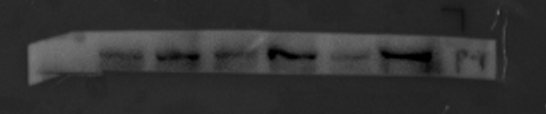


**70**

**100**




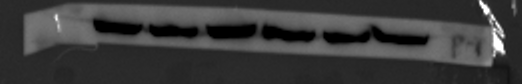


**100**

**70**

TBK1




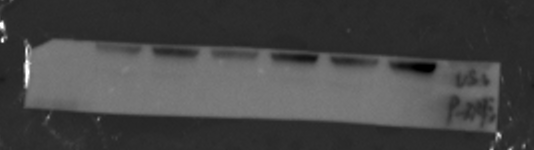


**55**

**40**

p-IRF3




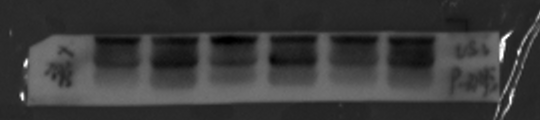


**40**

**55**

IRF3


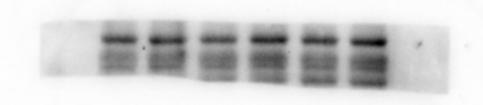

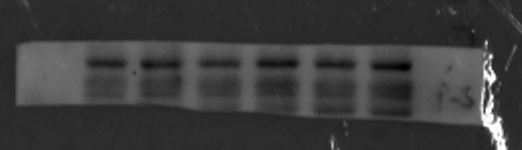


**35**

**40**

p-STING

STING




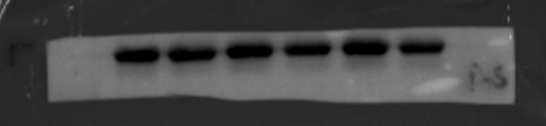


**35**

**40**


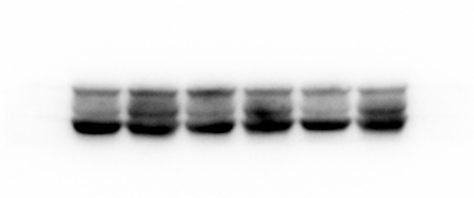

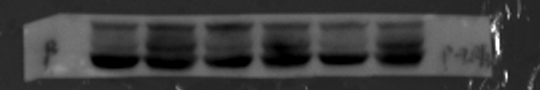


β-actin

**55**

**40**
