# Supplementary material for: HDAC6 deacetylates TRIM56 to negatively regulate cGAS-STING-mediated type I interferon responses
Source: EMBO Rep. 2025 Jan 2;26(3):720–47. doi: 10.1038/s44319-024-00358-5 (PMC11811133; doi:10.1038/s44319-024-00358-5)
Supplement: Supplementary file 15 — Expanded View Figures [file 44319_2024_358_MOESM15_ESM.pdf]

Expanded View Figures

Figure EV1. HDAC6 negatively regulates IFN production.

(**A**, **B**) N2a cells were transfected with negative control (NC) or HDAC6 siRNAs for 72 h. Knockdown efficacy of HDAC6 siRNAs in N2a cells (**A**). RT-qPCR analysis of the mRNA expression of *Ifnb1*, C*xcl10* and *Isg15* in N2a cells transfected with DHAC6 siRNAs for 72 h followed by infection with HSV-1 (MOI = 3) for 12 h (**B**). Data were analyzed using the unpaired *t* test, which are shown as mean ± SD ($n = 3$ biological replicates). (**C**) Western blot analysis of the indicated proteins in BV2 cells infected with HSV-1 (MOI = 3) for different time points. (**D–F**) N2a cells were transfected with vector, HDAC6 WT or deacetylase activity mutation (HD1/2 m) for 72 h followed by infection without or with HSV-1 (MOI = 3) for 12 h. (**D**, **E**) Western blot analysis of the indicated proteins in cells. (**F**) The mRNA expression of *Isg15*, *Isg20* and *Mx2* was detected by RT-qPCR assay. Data were analyzed using the unpaired t-test, which are shown as mean ± SD ($n = 3$ biological replicates). (**G**, **H**) Effect of tubacin on IFN production. N2a cells (**G**) and BV2 cells (**H**) were infected with HSV-1 (MOI = 3) in the presence or absence of tubacin (1 μM) for 12 h, and RT-qPCR analysis was performed to detect the expression of *Ifnb1, ifnα4, cxcl10, isg15* genes. Data were analyzed using the unpaired t-test, which are shown as mean ± SD ($n = 3$ biological replicates). (**I**) Western blot analysis of tubacin's effect on TBK1 activation. Source data are available online for this figure.

 © The Author(s)

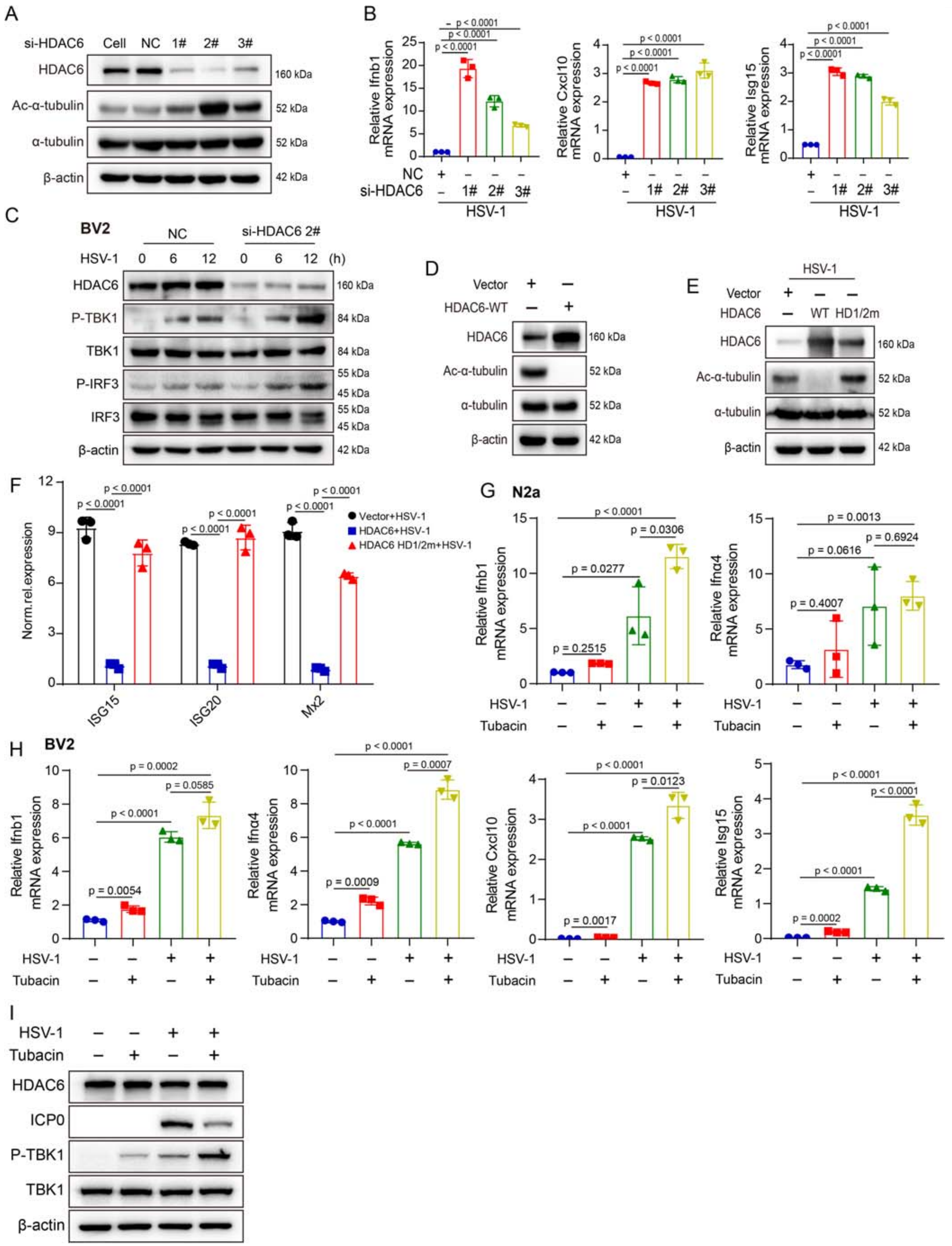
A
si-HDAC6 Cell NC 1# 2# 3#
HDAC6
Ac-α-tubulin
α-tubulin
β-actin
160 kDa
52 kDa
52 kDa
42 kDa
B
Relative Ifnb1 mRNA expression
Relative Cxcl10 mRNA expression
Relative Isg15 mRNA expression
p < 0.0001
NC
si-HDAC6
1# 2# 3#
HSV-1
C
BV2
NC si-HDAC6 2#
HSV-1 0 6 12 0 6 12 (h)
HDAC6
P-TBK1
TBK1
P-IRF3
IRF3
β-actin
160 kDa
84 kDa
84 kDa
55 kDa
45 kDa
55 kDa
45 kDa
42 kDa
D
Vector
HDAC6-WT
HDAC6
Ac-α-tubulin
α-tubulin
β-actin
E
HSV-1
Vector
HDAC6 WT HD1/2m
HDAC6
Ac-α-tubulin
α-tubulin
β-actin
F
Norm.rel.expression
p < 0.0001
Vector+HSV-1
HDAC6+HSV-1
HDAC6 HD1/2m+HSV-1
ISG15
ISG20
Mx2
G N2a
Relative Ifnb1 mRNA expression
Relative Ifnα4 mRNA expression
p < 0.0001
p = 0.0306
p = 0.0277
p = 0.2515
p = 0.0013
p = 0.6924
p = 0.0616
p = 0.4007
HSV-1
Tubacin
H BV2
Relative Ifnb1 mRNA expression
Relative Ifnα4 mRNA expression
Relative Cxcl10 mRNA expression
Relative Isg15 mRNA expression
p = 0.0002
p = 0.0585
p < 0.0001
p = 0.0054
p < 0.0001
p = 0.0007
p = 0.0009
p = 0.0123
p = 0.0017
p = 0.0002
HSV-1
Tubacin
I
HSV-1
Tubacin
HDAC6
ICP0
P-TBK1
TBK1
β-actin

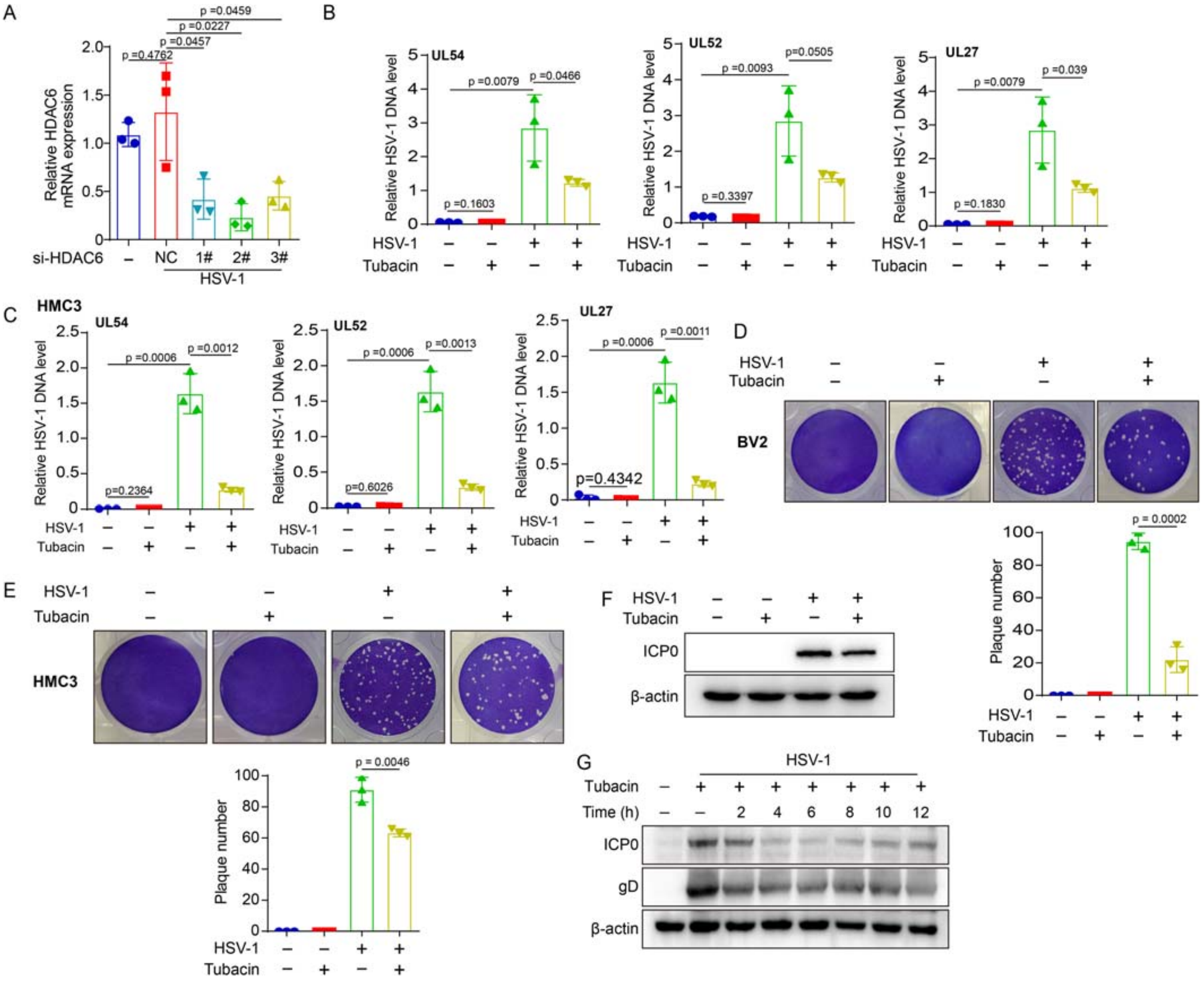

Figure EV2. HDAC6 promotes HSV-1 infection.

(**A**, **B**) RT-qPCR analysis of the mRNA expression of *HDAC6* in N2a cells transfected with HDAC6 siRNA and infected with HSV-1 (MOI = 3) for 12 h. Data were analyzed using the unpaired *t* test, which are shown as mean ± SD (*n* = 3 biological replicates). (**B**, **C**) The DNA copy number of viral genes in BV2 (**B**) and HMC3 cells (**C**) infected with HSV-1 (MOI = 3) in the presence or absence of tubacin (1 μM) for 12 h. Data were analyzed using the unpaired *t* test, which are shown as mean ± SD (*n* = 3 biological replicates). (**D**, **E**) Viral plaque assays of BV2 (**D**) and HMC3 cells (**E**) treated with or without HSV-1 (MOI = 3) in the presence or absence of tubacin (1 μM) for 12 h. The number of plaques was calculated. Data were analyzed using the unpaired *t* test, which are shown as mean ± SD (*n* = 3 biological replicates). (**F**, **G**) Western blot assay was performed to assess the levels of ICP0 and gD in N2a cells infected with HSV-1 (MOI = 3) for 12 h followed by treatment with tubacin (1 μM) in the indicated times of HSV-1 infection. Source data are available online for this figure.

© The Author(s)

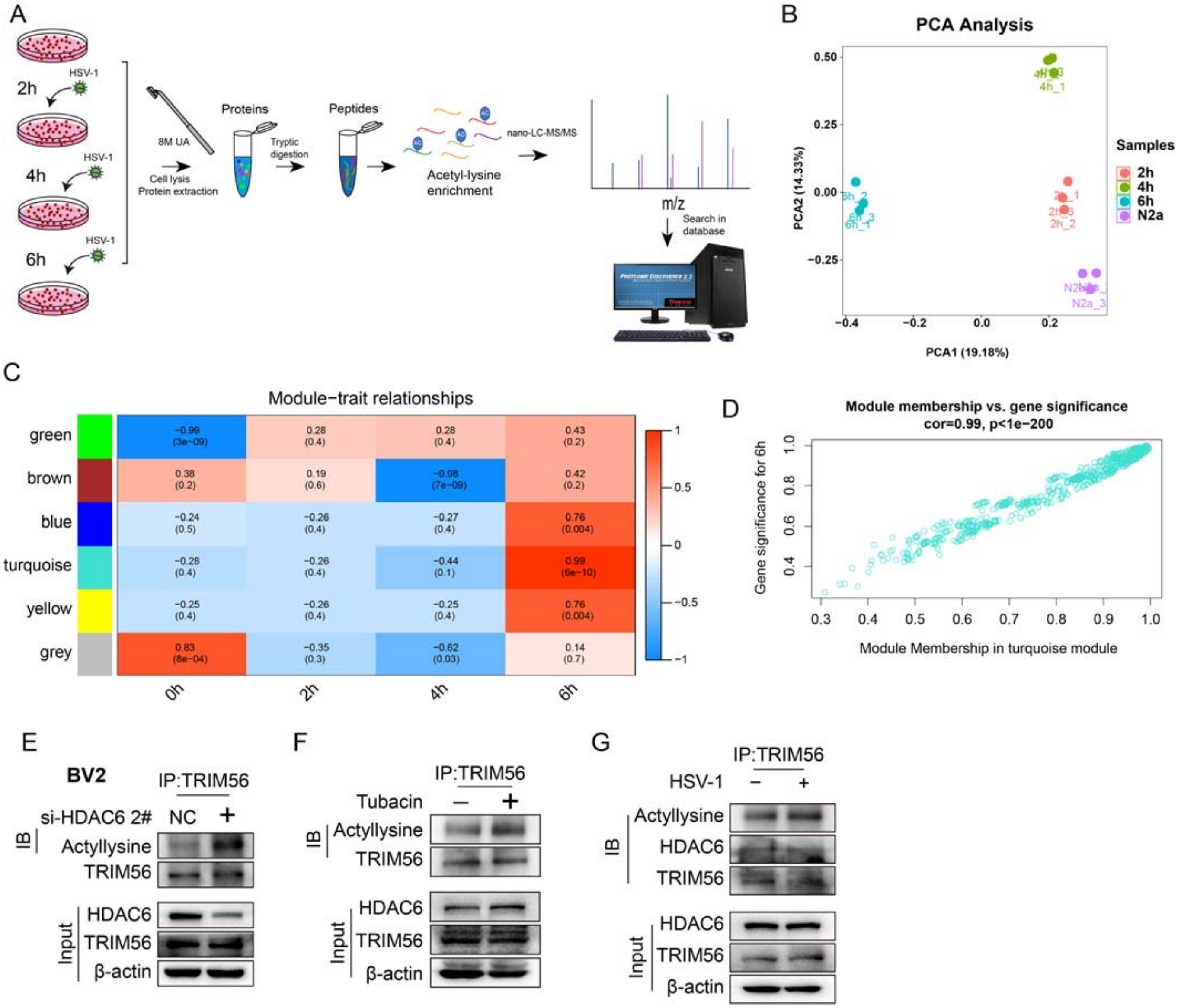

Figure EV3. HDAC6 regulates TRIM56 deacetylation in early stages of HSV-1 infection.

(**A**) Workflow diagram illustrating the process of profiling various time points in the acetylome of N2a cells infected with HSV-1 (MOI = 10). (**B**) PCA analysis of each sample. (**C**) Each row corresponds to a module feature point, and each column corresponds to the HSV-1 (MOI = 10) infected time point. The right panel represents the range of correlations: red indicates positive correlations and blue indicates negative correlations; numbers indicate correlations and significance. (**D**) Correlations between sites within the turquoise module and highly correlated sites following a 6 h infection. (**E–G**) Immunoprecipitation analysis of the acetylation of TRIM56 in BV2 cells transfected with or without HDAC6 siRNA for 72 h (**E**), and treated with or without tubacin (1 μM) (**F**), and infected with HSV-1 (MOI = 3) for 12 h (**G**). Source data are available online for this figure.

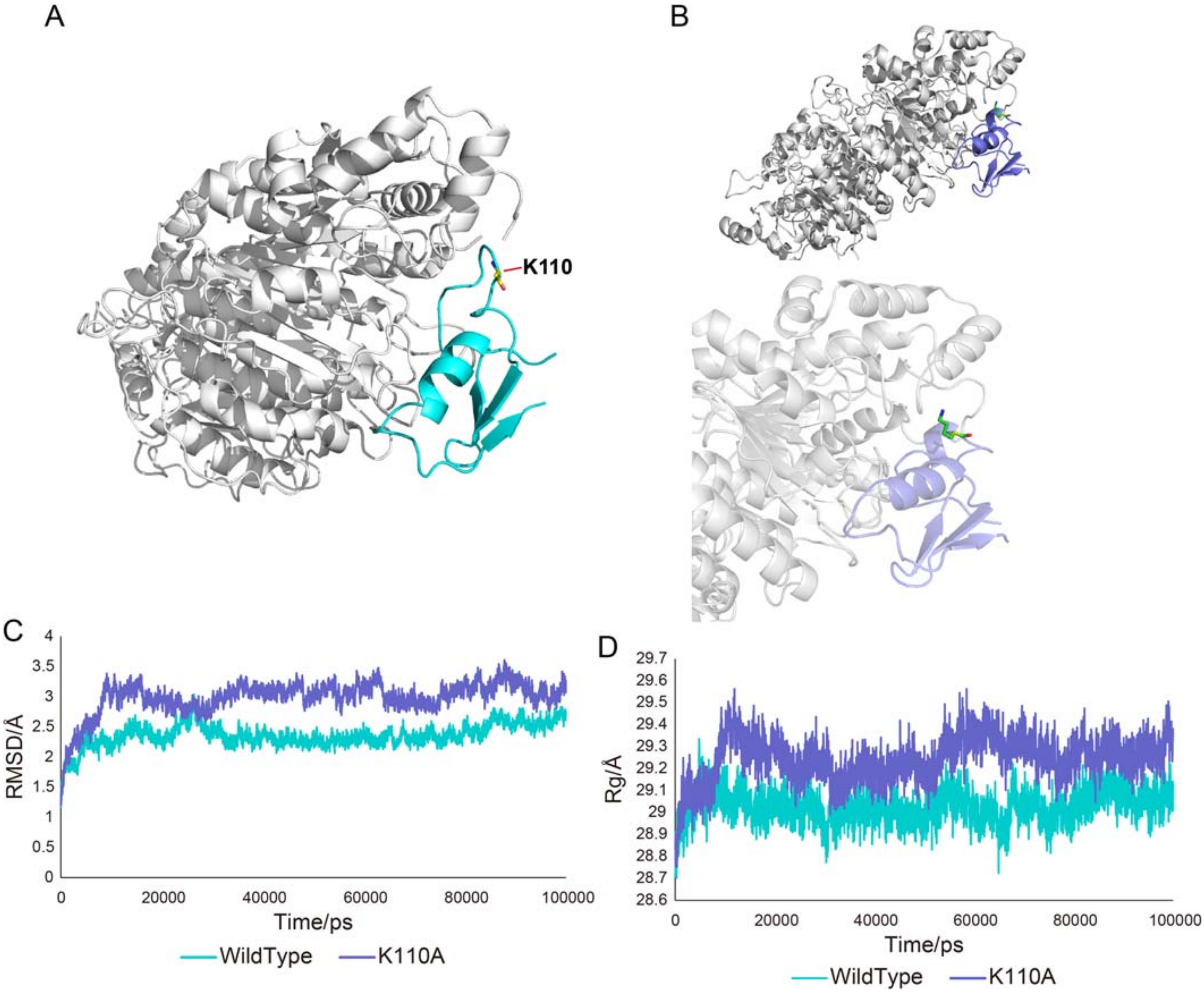

Figure EV4. TRIM56 positively regulates IFN production.

(**A**, **B**) Models depicting the predictable binding between HDAC6 CD1-CD2 (grey) and TRIM56 B box1 domain (cyan) (F) or TRIM56 K110A (G). (**C**, **D**) The plot comparing root mean square deviation (RMSD) (K) or Radius of gyration (Rg) (L) versus simulation time (ps) for WT and K110A mutant. Source data are available online for this figure.

 © The Author(s)

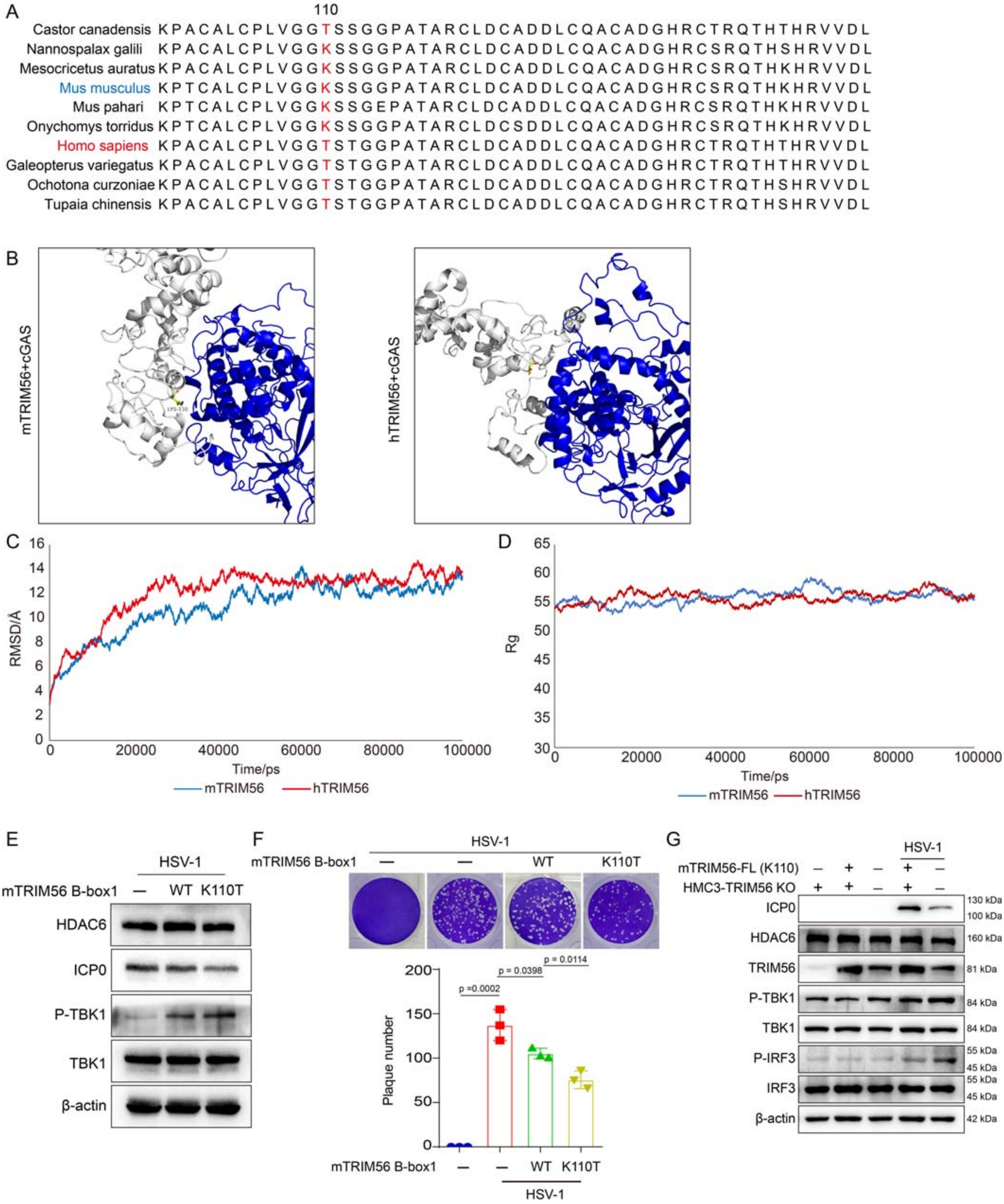
A
110
Castor canadensis KPACALCPLVGGTSSGGPATARCLDCADDLCQACADGHRCTRQTHTHRVVDL
Nannospalax galili KPACALCPLVGGKSSGGPATARCLDCADDLCQACADGHRCSRQTHSHRVVDL
Mesocricetus auratus KPACALCPLVGGKSSGGPATARCLDCADDLCQACADGHRCSRQTHKHRVVDL
Mus musculus KPTCALCPLVGGKSSGGPATARCLDCADDLCQACADGHRCSRQTHKHRVVDL
Mus pahari KPTCALCPLVGGKSSGEPATARCLDCADDLCQACADGHRCSRQTHKHRVVDL
Onychomys torridus KPTCALCPLVGGKSSGGPATARCLDCSDDLCQACADGHRCSRQTHKHRVVDL
Homo sapiens KPACALCPLVGGTSTGGPATARCLDCADDLCQACADGHRCTRQTHTHRVVDL
Galeopterus variegatus KPACALCPLVGGTSTGGPATARCLDCADDLCQACADGHRCTRQTHTHRVVDL
Ochotona curzoniae KPACALCPLVGGTSTGGPATARCLDCADDLCQACADGHRCTRQTHSHRVVDL
Tupaia chinensis KPACALCPLVGGTSTGGPATARCLDCADDLCQACADGHRCTRQTHSHRVVDL
B
mTRIM56+cGAS
hTRIM56+cGAS
C
RMSD/Å
Time/ps
mTRIM56
hTRIM56
D
Rg
Time/ps
mTRIM56
hTRIM56
E
HSV-1
mTRIM56 B-box1 — WT K110T
HDAC6
ICP0
P-TBK1
TBK1
β-actin
F
HSV-1
mTRIM56 B-box1 — — WT K110T
p =0.0002
p = 0.0398
p = 0.0114
Plaque number
mTRIM56 B-box1 — — WT K110T
HSV-1
G
HSV-1
mTRIM56-FL (K110) — + — + —
HMC3-TRIM56 KO + + — + —
ICP0
HDAC6
TRIM56
P-TBK1
TBK1
P-IRF3
IRF3
β-actin
130 kDa
100 kDa
160 kDa
81 kDa
84 kDa
84 kDa
55 kDa
45 kDa
55 kDa
45 kDa
42 kDa

◀ **Figure EV5. Species-specific amino acids at position 110 of TRIM56 K110 lead to different IFN responses.**

(**A**) Alignment of amino acids 98–149 of TRIM56 B-box1 from different species. (**B**) Molecular docking conformation of murine full-length TRIM56 K110 (mTRIM56) or K110T human full-length TRIM56 T110 (hTRIM56) (grey) and cGAS crystals (blue). (**C**) Plot of root mean square deviation (RMSD) versus simulation time (ps) for mTRIM56 and hTRIM56. (**D**) Rg comparison across the 100 ns molecular dynamic simulation of mTRIM56 and hTRIM56. (**E**) N2a cells transfected with mTRIM56 B-box1 or B-box1 K110T for 72 h followed by infection with HSV-1 (MOI = 3) for 12 h, and then western blot analysis of the cGAS-STING signaling pathway. (**F**) Viral plaque assays of BV2 cells transfected with mTRIM56 or mTRIM56 K110T followed by infection with HSV-1 (MOI = 3) for 12 h. The number of plaques was calculated (right). Data were analyzed using the unpaired *t* test, which are shown as mean ± SD ($n = 3$ biological replicates). (**G**) Western blot analysis of TBK1 and IRF3 activation in HMC3 TRIM56-KO12 cells transfected with mTRIM56 and then infected with HSV-1 (MOI = 3) for 12 h. Source data are available online for this figure.

 © The Author(s)
