# Supplementary material for: HDAC6 deacetylates TRIM56 to negatively regulate cGAS-STING-mediated type I interferon responses
Source: EMBO Rep. 2025 Jan 2;26(3):720–47. doi: 10.1038/s44319-024-00358-5 (PMC11811133; doi:10.1038/s44319-024-00358-5)
Supplement: Supplementary file 14 — EV and Appendix Figures Source Data [file 44319_2024_358_MOESM14_ESM.zip › Soure data Figure EV and Appendix/Source data Appendix Figure S1.zip!/Source data Appendix Figure S1/Figure S1A,B.docx]

**Source Figure S1A *Trigeminus***

β-actin

**130**

**170**

HDAC6

**40**

**55**

**Source Figure 3A** ***Brain***

HDAC6

**100**

**170**

**130**

**70**

**55**

**40**

Ac-α-tubulin

**40**

**70**

**55**

α-tubulin

**40**

**70**

**55**

β-actin

**Source Figure S1B**

Acetyllysine

**170**

**130**

**100**

**70**

**55**

**40**

**35**

**170**

**70**

**25**

**35**

**40**

**55**

**100**

**130**
