# Supplementary material for: HDAC6 deacetylates TRIM56 to negatively regulate cGAS-STING-mediated type I interferon responses
Source: EMBO Rep. 2025 Jan 2;26(3):720–47. doi: 10.1038/s44319-024-00358-5 (PMC11811133; doi:10.1038/s44319-024-00358-5)
Supplement: Supplementary file 14 — EV and Appendix Figures Source Data [file 44319_2024_358_MOESM14_ESM.zip › Soure data Figure EV and Appendix/Source data Appendix Figure S5.zip!/Source data Appendix Figure S5/Figure S5A,B,E.docx]

**Source Figure S5A**

Acetyllysine

**15**

**25**

**35**

**40**

**55**

**70**

**100**

**130**

**170**

**Source Figure S5B**

**130**

**170**

HDAC6

β-actin

**40**

**55**

**Source Figure S5E**

HDAC6

**130**

**170**

β-actin

**55**

**40**
