# Supplementary material for: HDAC6 deacetylates TRIM56 to negatively regulate cGAS-STING-mediated type I interferon responses
Source: EMBO Rep. 2025 Jan 2;26(3):720–47. doi: 10.1038/s44319-024-00358-5 (PMC11811133; doi:10.1038/s44319-024-00358-5)
Supplement: Supplementary file 14 — EV and Appendix Figures Source Data [file 44319_2024_358_MOESM14_ESM.zip › Soure data Figure EV and Appendix/Source data Appendix Figure S6.zip!/Source data Appendix Figure S6/Figure S6C.docx]

**Source Figure S6C**

**70**

**100**

P-TBK1

**70**

**100**

TBK1

P-IRF3

**55**

**40**

**40**

**55**

IRF3

**40**

**55**

β-actin
