# Supplementary material for: HDAC6 deacetylates TRIM56 to negatively regulate cGAS-STING-mediated type I interferon responses
Source: EMBO Rep. 2025 Jan 2;26(3):720–47. doi: 10.1038/s44319-024-00358-5 (PMC11811133; doi:10.1038/s44319-024-00358-5)
Supplement: Supplementary file 14 — EV and Appendix Figures Source Data [file 44319_2024_358_MOESM14_ESM.zip › Soure data Figure EV and Appendix/Source data Figure EV1.zip!/Source data Figure EV1/Figure EV1A,C,D,E,I.docx]

**Source Figure EV1A**

**170**

**130**

**100**

HDAC6

**35**

**55**

**40**

Ac-α-tubulin

α-tubulin

**55**

**40**

**40**

**55**

β-actin

**Source Figure EV1C**

**130**

**100**

P-TBK1

HDAC6

**70**

**100**

TBK1

**70**

**100**

**55**

**40**

P-IRF3

IRF3

**40**

**55**

**40**

**55**

β-actin

**Source Figure EV1D**

Ac-α-tubulin

**130**

**170**

HDAC6

**40**

**55**

**40**

**55**

α-tubulin

**55**

**40**

β-actin

**Source Figure EV1E**

**170**

**130**

Ac-α-tubulin

**55**

**40**

**40**

**55**

α-tubulin

**40**

**55**

β-actin

**Source Figure EV1I**

**170**

**130**

HDAC6

ICP0

**、**

**100**

**130**

**170**

**70**

**100**

P-TBK1

**70**

**100**

TBK1

**55**

**40**

β-actin
