# Supplementary material for: HDAC6 deacetylates TRIM56 to negatively regulate cGAS-STING-mediated type I interferon responses
Source: EMBO Rep. 2025 Jan 2;26(3):720–47. doi: 10.1038/s44319-024-00358-5 (PMC11811133; doi:10.1038/s44319-024-00358-5)
Supplement: Supplementary file 14 — EV and Appendix Figures Source Data [file 44319_2024_358_MOESM14_ESM.zip › Soure data Figure EV and Appendix/Source data Figure EV3.zip!/Source data Figure EV3/Figure EV3E,F,G.docx]

**Source Figure EV3E**

**55**

**70**

**100**

IB: Acetyllysine

**55**

**100**

**70**

IB: TRIM56

**170**

**130**

Input: HDAC6

**70**

**100**

Input: TRIM56

**55**

**40**

Input: β-actin

**Source Figure EV3F**

**55**

**100**

**70**

IB: Acetyllysine

**100**

**70**

**55**

IB: TRIM56

Input: HDAC6

**130**

**170**

**70**

**55**

Input: TRIM56

**40**

**55**

Input: β-actin

**Source Figure EV3G**

**40**

**55**

**70**

**100**

**130**

**170**

IB: HDAC6

IB: Acetyllysine

**35**

**40**

**55**

**70**

**100**

**130**

**170**

**35**

**40**

**55**

**70**

**100**

**130**

**170**

IB: TRIM56

**130**

**170**

Input: HDAC6

Input: TRIM56

**100**

**70**

**40**

**55**

Input: β-actin
